# Ta-induced electronic reconstruction in CaSiO_3_: wide-band-gap modulation, emergent metallic behavior, and multifunctional response from first-principles

**DOI:** 10.1039/d6ra06904e

**Published:** 2026-09-01

**Authors:** Qaiser Rafiq, Sikander Azam, Khalid M. Elhindi, Muhammad Tahir Khan, Sisi Wang, Umair Rashid

**Affiliations:** a Faculty of Engineering and Applied Sciences, Department of Physics, RIPHAH International University Islamabad Pakistan qrafique1@gmail.com physicst.sikander@gmail.com; b Plant Production Department, College of Food & Agriculture Sciences, King Saud University P.O. Box 2460 Riyadh 11451 Saudi Arabia; c Key Laboratory of Urban Rail Transit Intelligent Operation and Maintenance Technology & Equipment of Zhejiang Province, College of Engineering, Zhejiang Normal University Jinhua 321004 People's Republic of China sisi.wang@zjnu.edu.cn; d School of Physics and Optoelectronic Engineering, Beijing University of Technology Beijing 100124 China; e University of West Bohemia in Pilsen, New Technologies – Research Centre 8 Univerzitní Pilsen 301 00 Czech Republic

## Abstract

A comprehensive first-principles investigation was conducted to clarify the impact of Ta substitution at the Si site on the structural, electronic, optical, thermoelectric, mechanical, and magnetic properties of CaSiO_3_. The pristine cubic model has an optimized lattice parameter of 3.607 Å, whereas Ta incorporation at 12.5% and 25% preserves the host framework while inducing local B–O coordination rearrangement. The equilibrium volume increases from 2533.4554 Bohr^3^ for pristine CaSiO_3_ to 2755.1025 and 3155.7362 Bohr^3^ for the 12.5% and 25% Ta-substituted systems, respectively. The EOS-derived bulk modulus decreases from 215.3005 GPa to 167.1973 and 169.5482 GPa, indicating greater hydrostatic compressibility of both substituted compositions relative to pristine CaSiO_3_, with a slight recovery at 25% Ta. The negative formation energies of −4.11, −3.28, and −2.96 eV per atom support the energetic feasibility of the modeled compositions relative to the chosen elemental reference states. The absence of imaginary phonon branches further confirms the dynamical stability of both pristine and Ta-substituted structures. Electronically, pristine CaSiO_3_ exhibits an indirect band gap of 3.432 eV dominated by O-2p states at the valence band and Si-3p states at the conduction band, whereas Ta substitution introduces Ta-5d-dominated donor-derived states with O-2p participation near the Fermi level, producing a degenerate n-type/incipient metallic regime at 12.5% Ta and a metallic n-type regime at 25% Ta. This concentration-dependent electronic evolution is independently supported by the Fermi-surface topology. Within the stoichiometric defect-free model, the corresponding nominal donor-density estimates increase from the negligible-carrier parent limit to 2.45 × 10^21^ cm^−3^ and 4.28 × 10^21^ cm^−3^ for 12.5% and 25% Ta, respectively; these values represent formal donor-density estimates rather than independently calculated equilibrium free-carrier concentrations. The electronic restructuring is also reflected in the calculated interband optical response. Pristine CaSiO_3_ exhibits strong deep-ultraviolet absorption, whereas Ta incorporation reduces the intensity of interband transitions, redistributes the spectral weight, and smooths the profiles of *ε*_2_(*ω*), *α*(*ω*), *k*(*ω*), *σ*(*ω*), and *R*(*ω*). In addition, Ta substitution enhances the calculated plasmon-related loss features in the ∼8–13 eV energy range. The thermoelectric response is strongly composition dependent. Pristine CaSiO_3_ retains a positive Seebeck coefficient and weak band-derived charge transport, whereas 12.5% Ta produces negative electron-like thermopower and the most favorable calculated PF/τ balance over much of the investigated temperature range. At 25% Ta, σ/τ and the electronic heat-transport contribution increase markedly, but the small Seebeck magnitude suppresses the power factor and produces a near-zero *ZT* over most of the calculated range. Thus, increased Ta-induced conductivity does not translate monotonically into improved thermoelectric performance. Mechanical analysis shows a systematic increase in the elastic-constant-derived response with Ta content, quantified by *C*_11_ (248 → 296 GPa), *C*_12_ (115 → 144 GPa), *C*_44_ (90 → 117 GPa), *B* (160 → 194 GPa), *G* (84 → 106 GPa), *E* (212 → 261 GPa), *θD* (590 → 672 K), and *H* (7.5 → 11.2 GPa).These small-strain trends are treated separately from the EOS-derived bulk modulus and are interpreted together with Ta-induced local B–O coordination changes and complementary COHP/ELF evidence for electronic reorganization, without using the mechanical data as direct proof of monotonic strengthening or increasing covalency of individual Ta–O bonds. Magnetically, pristine CaSiO_3_ remains non-magnetic, whereas Ta incorporation induces finite spin polarization at 12.5% (0.43417 *µ*_B_), associated with Ta-5d-dominated near-Fermi-level states, O-2p participation, and a spatially distributed Ta/O-centered spin imbalance. At 25% Ta, the magnetic response is nearly quenched (−0.00132 *µ*_B_), with very small mixed-sign Ta/O local moments and a negligible interstitial contribution (0.00001 *µ*_B_), indicating strong spatial compensation of the induced spin polarization. Spin-resolved PDOS, site-resolved magnetic moments, and real-space spin-density distributions consistently show that the suppression at higher Ta content originates from the reduction and spatial compensation of the local spin polarization rather than from an assumed long-range magnetic-ordering mechanism. These results demonstrate that Ta substitution enables composition-dependent modulation of the electronic, optical, thermoelectric, mechanical, and magnetic responses of CaSiO_3_ while preserving the underlying perovskite framework. The calculated trends therefore support further experimental and application-specific evaluation of Ta-substituted CaSiO_3_ as a compositionally tunable functional oxide.

## Abstract 2

Ta substitution drives donor-derived states to *E*_F_, yielding degenerate n-type behavior at 12.5% Ta and a metallic n-type state at 25%; the accompanying optical, thermoelectric and spin responses evolve strongly with composition.

## Graphical abstract

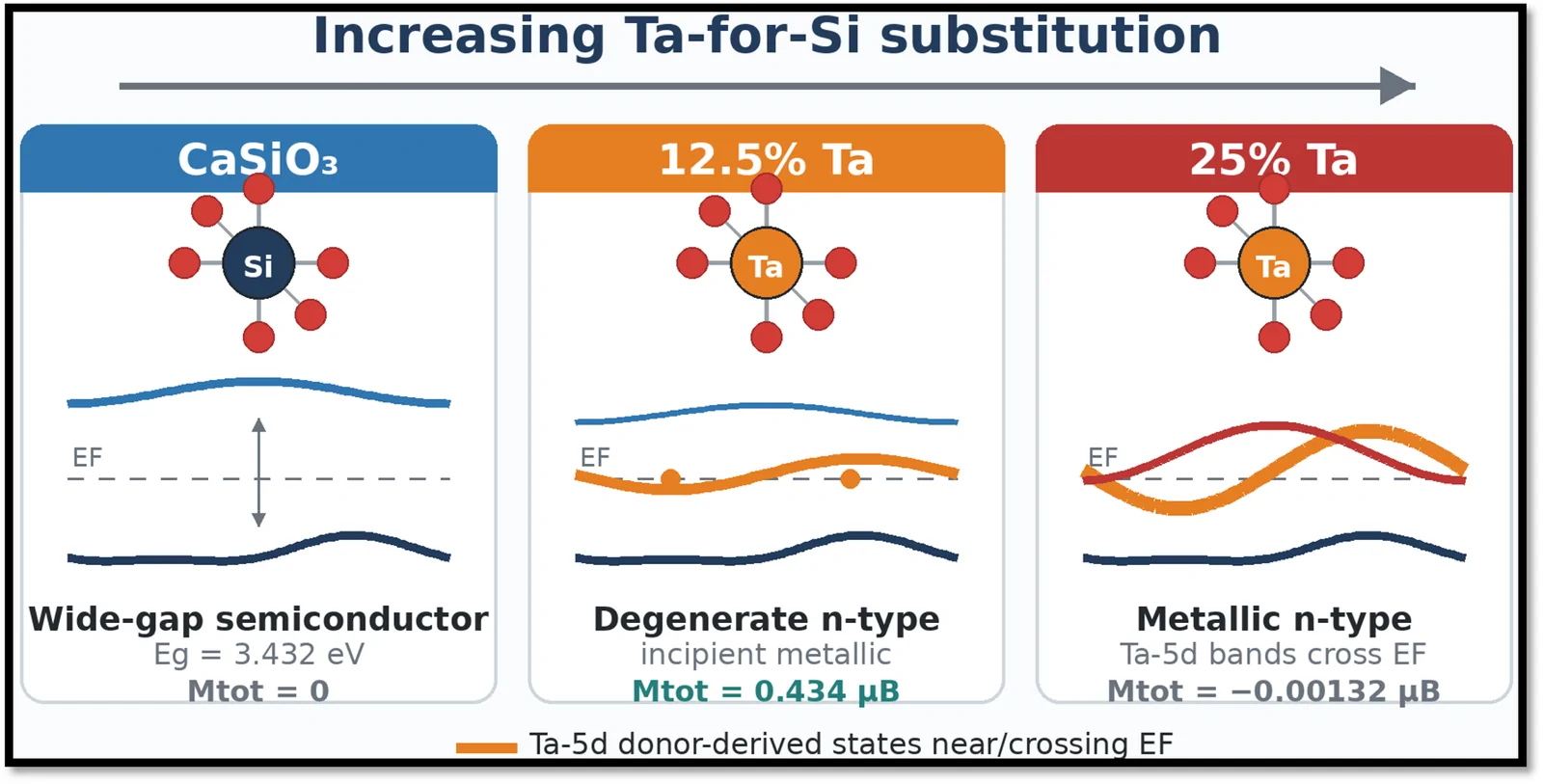

## 1. Introduction and research context

Calcium silicate (CaSiO_3_), commonly known as wollastonite, is an inorganic material widely used in ceramics, surface coatings, biocomposites, and eco-friendly glass-ceramic applications. Recent studies have shown that CaSiO_3_ can be synthesized in a controlled manner using inexpensive or waste-derived raw materials, where processing conditions and sintering temperature play critical roles in determining its crystal structure, density, and optical band gap.^1^ Sustainable synthesis strategies have also demonstrated that industrial by-products can be used to produce high-quality calcium silicate, making it suitable for large-scale and environmentally friendly applications.^2^ Moreover, solid-state preparation methods allow the formation of selected polymorphs, such as wollastonite-2M, with controllable microstructures for enhanced functional performance.^3^ A closely related development is the increasing use of CaSiO_3_-based materials for reinforcement and low-carbon cement systems, where wollastonite, either in fibrous or particulate form, improves mechanical strength, durability, and overall sustainability.^4^ Moreover, first principle studies demonstrate that density functional theory (DFT) provides a strong paradigm to understand how structural stability and electronic and transport properties are interrelating which can also serve as good guidance to the design of effective oxide materials.^5^ Calcium silicate (CaSiO_3_) is also important in glass and ceramic systems because of its high biocompatibility, which can be further modified through compositional tuning. For example, in B_2_O_3_-modified apatite–wollastonite glass-ceramics, key properties such as hardness, microstructure, and radiation-shielding performance can be controlled by adjusting the CaO–SiO_2_ network structure.^6^ In addition, composite layers containing wollastonite and ZnO exhibit strong antibacterial activity and improved anticorrosion behavior, highlighting the importance of CaSiO_3_-based materials in biomedical applications.^7^*In vivo* biocompatibility studies of wollastonite- and Fe_2_O_3_-containing ceramics have also shown promising results, thereby expanding the possible biological applications of CaSiO_3_-based materials.^8^ Laser-processed eutectic systems containing wollastonite have further been proposed as potential bone-repair materials, demonstrating the adaptability of CaSiO_3_ to advanced fabrication and processing routes in the biomedical field.^9^ From a theoretical perspective, full-potential DFT studies of perovskite-type oxides show that lattice symmetry and atomic coordination strongly influence electronic band behavior and thermodynamic stability. These insights support the use of first-principles methods to establish clear relationships between crystal chemistry and functional properties in ceramic oxide systems.^10^

Although CaSiO_3_-based materials have attracted increasing attention, the influence of tetravalent or aliovalent ion substitution on their electronic, optical, and transport properties remains insufficiently explored, both experimentally and theoretically. In contrast, several related oxide and perovskite systems have been systematically investigated using first-principles methods. For example, detailed DFT simulations of BaTiO_3_ have shown that its electronic band structure and optical absorption behavior are highly sensitive to structural and temperature-driven changes.^11^ These findings indicate that controlled cation substitution can strongly modify the electronic states, optical transitions, and charge-transport characteristics of oxide perovskites. Therefore, a focused first-principles investigation of Ta-substituted CaSiO_3_ is necessary to clarify how B-site substitution affects its multifunctional response. An example of this is that DFT calculations of BaTiO_3_ have shown that its optical absorption properties and band structure are very sensitive to temperature changes.^11^ Likewise, co-doped Ba–Sr–Ti–Zr perovskite systems have shown improved functional properties, as demonstrated through computational approaches such as WIEN2k and BoltzTraP. These systems exhibit tunable electronic band structures, enhanced optical response, and improved thermoelectric performance, with figure-of-merit (*ZT*) values reaching ranges relevant for practical applications.^12^ Similarly, B-site substitution in Sb-doped BaFeO_3_, particularly at 12.5% and 25% doping concentrations, has been reported to strongly influence the optical response, density of states, and thermoelectric behavior.^13^ Collectively, these findings suggest that controlled cation substitution is an effective strategy for tailoring the multifunctional properties of complex oxide materials.

This trend has been further strengthened by recent advances in first-principles methods, where electronic structure, optical response, and thermoelectric transport can be examined within a unified theoretical framework. For example, La–Sm–Sr–Mn oxide systems have been simulated using the WIEN2k package to evaluate their electronic, optical, magnetocaloric, and thermoelectric properties.^14^ Similarly, DFT studies have also shown that the substitution of Sr in LaCoO_3_ causes doping-induced metallization and enhanced charge carrier transport properties.^15^ Moreover, recent developments in entropy-engineered perovskite thermoelectrics have demonstrated the potential of multi-cation substitution strategies. These approaches enable the design of materials with substantially reduced lattice thermal conductivity and enhanced power factors, thereby improving overall thermoelectric performance.^16^ In advanced functional oxides, tantalum (Ta) incorporation has emerged as an effective route for regulating the electronic structure of wide-band-gap semiconductors. Specifically, Ta incorporation in oxide hosts is commonly described within a formal Ta^5+^ ionic picture, under which donor-like states, increased carrier concentration, and enhanced electrical conductivity may arise. For example, Ta-doped TiO_2_ has been reported to exhibit improved charge transport and enhanced photocatalytic activity, mainly due to the formation of shallow donor levels and reduced charge recombination.^17^ Similarly, Ta incorporation in ZnO systems increases electrical conductivity while maintaining high optical transparency, making these materials suitable for optoelectronic applications.^18^ In perovskite-type oxides, Ta substitution can modify lattice symmetry, band dispersion, and carrier transport, thereby enhancing overall functional performance.^19^ Ta-doped SrTiO_3_ systems also show improved thermoelectric behavior, where carrier concentration and lattice thermal conductivity are optimized, leading to enhanced figure-of-merit (*ZT*) values.^20^ Moreover, defect engineering associated with Ta incorporation, particularly through the control of oxygen-vacancy concentration, has been shown to enhance visible-light absorption and catalytic activity in oxide materials.^21^ Overall, these studies confirm that Ta substitution introduces donor-like electronic states that are effective for tailoring optical and transport properties. Therefore, aliovalent Ta substitution provides a useful route for examining how donor-type B-site substitution modifies the electronic structure and associated functional response of CaSiO_3_ and related oxide systems.^22^

Recent first-principles investigations have demonstrated that tantalum (Ta) can act as an effective electronic modifier in transition-metal oxides (TMOs). Specifically, Ta incorporation can introduce donor-like electronic states, increase carrier concentration, and facilitate charge transport. For instance, Ta-doped TiO_2_ systems have shown enhanced visible-light absorption and improved photocatalytic performance, mainly because of higher carrier mobility, shallow donor-state formation, and reduced charge recombination. More generally, DFT+*U* studies of oxygen-deficient ceria surfaces have shown that oxygen-vacancy formation can introduce localized electronic states within the gap and redistribute excess charge around the reduced cation environment.^23^ In addition to optical applications, aliovalent Ta substitution plays an important role in regulating defect chemistry and redox behavior in oxide materials, which is essential for energy-conversion and catalytic processes.^24^ Moreover, DFT experiments of ABO_3_-type perovskites indicate that there are high flexibility correlations between a substitution of cations and the resulting optoelectronic and thermoelectric characteristics, and therefore, compositional design is critical.^25^ Moreover, computational studies on oxide perovskites have shown that cation engineering strongly influences electronic structure and transport behavior, which can be exploited to enhance thermoelectric performance and multifunctional response.^26,27^ More broadly, recent findings confirm that selective doping provides a reliable route for controlling optical response and charge conduction in complex oxide materials.^28^ Therefore, Ta incorporation at controlled concentrations of 12.5% and 25% can be considered an effective strategy for modulating the electronic structure, carrier transport, and functional properties of CaSiO_3_-based materials. The choice of Ta as the dopant is driven by its many desirable electronic, chemical and structural properties over many traditional transition metal dopants. Unlike 3d dopants like Mn, Fe, Co, Ni or Cu, which can create a deeply localized d state, a deep defect level or an unwanted magnetic scattering, Ta can create non-magnetic, extended 5d states that hybridize well with O-2p states near the conduction-band edge. In many oxide environments, Ta is commonly described by the formal Ta^5+^ oxidation state. In the present work, however, this notation is used only for nominal electron counting and is not interpreted as a directly calculated +5 atomic charge. Within this formal Ta^5+^/Si^4+^ ionic picture, Ta-for-Si substitution corresponds to one additional donor electron per substituted site. The fact that Ta is larger than Si, but still has a stable Ta–O coordination is, together with the negative formation energies and the phonon stability, a reason for its choice as a suitable dopant for simultaneous tuning of the electronic structure, optical response, transport behavior and mechanical and magnetic responses of CaSiO_3_. Recent first-principles calculations on CaSiO_3_ perovskite stability and experimental/theoretical studies indicating that Ta doping can effectively modify the structural and optoelectronic response of SrTiO_3_-based perovskite oxides^29,30^ have supported this choice. Although CaSiO_3_-based materials have been widely studied, a comprehensive and systematic investigation of Ta-doped CaSiO_3_ remains limited.

To the best of our knowledge, no detailed DFT study has yet examined Ta substitution at the Si site of CaSiO_3_ at the investigated substitution levels of 12.5% and 25%. In particular, the influence of Ta incorporation on structural parameters, unit-cell volume, electronic band dispersion, optical transitions, and thermoelectric behavior has not been extensively explored. Since Ta doping in related oxide systems has been shown to induce band-gap modulation, carrier activation, and changes in carrier-phonon transport, the investigation of Ta-doped CaSiO_3_ is scientifically relevant for establishing concentration-dependent relationships between structural modification, electronic reconstruction, and transport response.

In this work, we present a first-principles investigation of CaSiO_3_ doped with 12.5% and 25% Ta (Ta-CS) to obtain a comprehensive understanding of its physical properties. Using a full-potential DFT framework combined with Boltzmann transport theory, we systematically evaluate structural relaxation, electronic structure, optical response, thermoelectric transport, mechanical properties, and magnetic response. Furthermore, this study examines how the intrinsic behavior of this wide-band-gap silicate is modified by Ta-for-Si substitution, with Ta^5+^/Si^4+^ used only as a nominal oxidation-state notation for formal electron counting. The present calculations therefore focus on establishing how Ta-for-Si substitution modifies the intrinsic structure–electronic–property relationships of CaSiO_3_, while assessment of specific technological applications requires additional application-oriented calculations and experimental validation.

### 1.1. Key contributions and scientific novelty

This work presents the first theoretical evidence of a doping-induced semiconductor-to-metal transition in Ta-substituted CaSiO_3_. First-principles calculations show that Ta incorporation strongly modifies the electronic structure through Ta-5d/O-2p hybridization, which introduces donor-like states near the Fermi level and drives the observed electronic transition while maintaining lattice stability. In addition, Ta-induced local coordination rearrangement and charge redistribution play key roles in tuning the main physical properties, including mechanical rigidity, spin polarization, and deep-ultraviolet optical absorption. The results also reveal a controllable transition between p-type and n-type transport behavior. The resulting picture links Ta-induced electronic reconstruction with the corresponding optical, thermoelectric, mechanical, and magnetic responses within a consistent first-principles framework.

## 2. First-principles modeling and computational strategy

First-principles calculations were carried out for pristine CaSiO_3_ and Ta-doped CaSi_1−*x*_Ta_*x*_O_3_ at doping concentrations of *x* = 12.5% and 25% using the WIEN2k simulation package.^31^ The calculations were performed within the framework of density functional theory (DFT) using the full-potential linearized augmented plane-wave (FP-LAPW) method, which provides high accuracy for electronic-structure calculations. The generalized gradient approximation (GGA) was employed to describe the exchange–correlation effects. A GGA+*U* correction was applied specifically to the Ta-5d states to properly describe the on-site Coulomb interactions associated with the dopant-derived d states. The use of Hubbard-corrected DFT provides an established framework for improving the description of localized or partially localized d states, while the effective interaction is understood to depend on the chemical and electronic environment.^32,33^ A comparable DFT+*U* treatment has also been employed for Ta-5d-containing perovskite systems, supporting its use for describing Ta-derived electronic states in oxide environments.^34^ This approach provides a more reliable treatment of electron-correlation effects in transition-metal-doped oxide systems and is commonly employed for materials containing partially occupied d- or f-electron states.^35^

The perfect crystal structure of CaSiO_3_ was modeled using a (1 × 1 × 1) primitive unit cell. For Ta substitution, a (2 × 2 × 2) supercell containing eight Si B-site positions was constructed and used consistently for both doped compositions. For the 12.5% Ta configuration, one of the eight Si atoms was replaced by Ta, yielding Ca_8_Si_7_TaO_24_, equivalent to CaSi_0_._875_Ta_0_._125_O_3_. Thus, the B-site sublattice contains 12.5% Ta and 87.5% Si. For the 25% Ta configuration, two of the eight Si atoms in the same (2 × 2 × 2) supercell were replaced by Ta, yielding Ca_8_Si_6_Ta_2_O_24_, equivalent to CaSi_0_._75_Ta_0_._25_O_3_, corresponding to 25% Ta and 75% Si on the B-site sublattice. Employing the same supercell size for both substituted compositions provides a consistent structural basis for examining the concentration-dependent effects of Ta incorporation. The corresponding lattice parameters, crystallographic symmetry, inequivalent atomic positions, and local structural modifications are discussed in Section 3.1. The energy–volume data were fitted using the third-order Birch–Murnaghan equation of state, while formation energies were evaluated relative to the corresponding elemental reference states. Phonon–dispersion calculations were additionally used to assess the dynamical stability of the optimized compositions from the presence or absence of imaginary-frequency modes.

In the present substitution model, no oxygen vacancies, cation vacancies, interstitials, or other explicit compensating point defects were introduced into the Ta-substituted supercells. No net supercell charge or artificial compensating background charge was imposed. The periodic DFT cells were treated as overall charge-neutral systems, with the total electronic population determined self-consistently from the atomic composition. In a formal ionic description, replacement of Si^4+^ by nominal Ta^5+^ corresponds to a donor-type aliovalent substitution and introduces one nominal donor electron per substituted Ta site in formal electron counting. However, these oxidation-state labels were not imposed as fixed ionic charges during the DFT calculations; rather, the redistribution and energetic accommodation of the additional electronic charge were determined self-consistently from the calculated electronic structure.

Accordingly, the present calculations describe the stoichiometric, defect-free electronic-compensation limit of Ta substitution. Within this model, the additional electronic population associated with Ta-for-Si substitution remains within the globally neutral electronic system and is redistributed self-consistently among the energetically available states of the substituted lattice. Its evolution is examined through the calculated band structure, DOS/PDOS, Fermi-surface topology, nominal donor-density analysis, and real-space charge-density distributions. Intrinsic defect-mediated compensation, particularly through native acceptor-type defects, may become relevant under particular experimental growth conditions; however, such competing defect configurations were not explicitly considered in the present work. Establishing their relative thermodynamic stability would require separate defect-formation-energy calculations as functions of chemical potential and Fermi level. Therefore, the present results should be interpreted as describing the intrinsic electronic response of the stoichiometric Ta-substitution models rather than a complete description of equilibrium defect chemistry under experimental growth conditions. In the calculations of the electronic structure, the valence configurations were considered to be Ca: 3s^2^, 3p^6^, 4s^2^, Si: 3s^2^, 3p^2^, O: 2s^2^, 2p^4^ and Ta: 5p^6^, 5d^3^, 6s^2^.These configurations include the principal orbital contributions required to describe the valence-band, conduction-band, and Ta-induced electronic states near the Fermi level. To obtain a consistent depiction of the wave functions in the muffin-tin spheres, the angular momentum expansion was initialized to *l*_ma*x*_ = 10, which incorporates the s, p, d, f, and higher-order terms and is computationally efficient.^36^ The plane-wave basis was regulated using the parameter *R*_MT_ × *K*_max_ = 7, which was a compromise that gave good convergence of both the valence states as well as the low-lying conduction bands.^37^

A cutoff parameter of *G*_max_ = 12 (Ry)^1/2^ was used to expand the charge density of pristine CaSiO_3_ and raised to 14 (Ry)^1/2^ to improve the description of the more pronounced charge redistribution due to Ta incorporation. Core electrons were considered within a fully relativistic treatment, whereas the valence electrons were treated through the scalar-relativistic approximation. A separation of −6 Ry was used to separate core and valence states, which is in line with established procedures in electronic structure computations.^38,39^ Monkhorst–Pack *k*-point grids of (8 × 8 × 8), (4 × 4 × 4), and (3 × 3 × 3) were employed for pristine CaSiO_3_, the 12.5% Ta configuration, and the 25% Ta configuration, respectively. Brillouin-zone sampling followed the Monkhorst–Pack scheme.^40^ The *k*-point density was increased where necessary to ensure an accurate description of the electronic and optical properties. The self-consistent field (SCF) calculations were continued until the total-energy difference between successive iterations was less than 10^−4^ Ry. The optical properties, including the dielectric function, refractive index, reflectivity, and absorption coefficient, were calculated using the OPTIC module, with a Gaussian broadening of 0.1 eV and dense *k*-point sampling to accurately describe interband transitions.

The corresponding extinction coefficient, optical conductivity, and energy-loss function were evaluated within the same optical-response framework to maintain consistency among the calculated spectra. Thermoelectric transport properties, including the Seebeck coefficient, were evaluated using the BoltzTraP code with dense transport meshes of 25 000–35 000 *k*-points over the temperature range of 50–800 K, which is relevant for silicate-based oxide materials.^41–43^ The transport quantities obtained from the electronic-band dispersion were treated separately from the nominal donor-density estimates discussed later in the manuscript. The latter are stoichiometric estimates based on formal one-electron donor counting for each Ta-for-Si substitution and should not be interpreted as independently calculated equilibrium free-carrier concentrations. In addition, the elastic and mechanical properties were obtained from the calculated elastic constants using the stress–strain method within the WIEN2k framework, allowing the evaluation of the bulk modulus, shear modulus, and mechanical stability criteria.^44^ The local bonding response was examined independently using energy-resolved crystal orbital Hamilton population (COHP) and real-space electron localization function (ELF) analyses. COHP was used to resolve bonding and antibonding contributions, whereas ELF was used to characterize the spatial redistribution of electron localization. Because integrated COHP values were not evaluated, these analyses were not used to quantitatively rank individual bond strengths. The optimized structural model corresponding to each composition was retained consistently throughout the associated electronic, optical, transport, elastic, magnetic, and charge-density analyses, providing a common basis for comparison among pristine, 12.5% Ta, and 25% Ta systems.

In addition, spin-polarized DFT calculations were performed to investigate magnetic behavior, local magnetic moments, and spin-dependent electronic-structure changes induced by Ta doping.^45^ For the magnetic analysis, the converged spin-polarized solutions were used to separate the calculated magnetic response into atom-centered and interstitial contributions. Site-resolved magnetic moments were obtained from the spin imbalance within the muffin-tin spheres of the inequivalent atomic sites, while the interstitial contribution accounts for the spin density distributed outside these atom-centered regions. Spin-resolved TDOS and PDOS were analyzed separately for the spin-up and spin-down channels to identify the orbital origin of the magnetic response, with particular attention to the Ta-5d and neighboring O-2p states. In parallel, the real-space spin-density difference, *ρs*(*r*) = *ρ*↑(*r*) – *ρ*↓(*r*), was examined to determine the spatial localization, redistribution, and compensation of the induced spin polarization with increasing Ta concentration. Together, these quantities provide complementary reciprocal- and real-space descriptions of the concentration-dependent magnetic response. Recent first-principles studies on perovskite and related functional materials have followed comparable computational routes, where structural optimization, elastic stability, electronic-state analysis, optical response, magnetic behavior, and thermoelectric transport are examined together to build a consistent picture of material performance.^46–51^

## 3. Results and discussion of electronic structure and multifunctional properties

### 3.1. Crystal structure and structural optimization

**Fig. 1:**
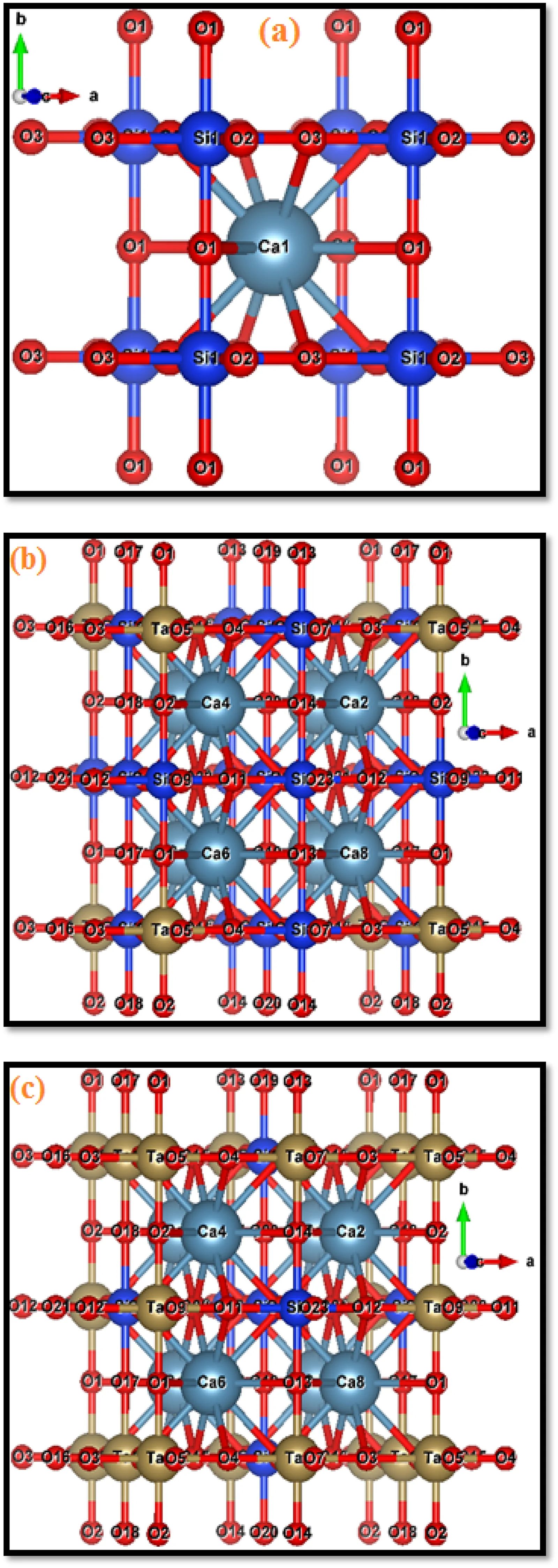
Optimized structural models of (a) pristine CaSiO_3_, (b) CaSi_0_._875_Ta_0_._125_O_3_ (12.5% Ta), and (c) CaSi_0_._75_Ta_0_._25_O_3_ (25% Ta). The pristine phase is derived from the 1 × 1 × 1 cubic *Pm*3̄*m* perovskite cell, whereas the Ta-substituted systems are represented using 2 × 2 × 2 supercells. One and two of the eight Si B-sites are replaced by Ta for the 12.5% and 25% compositions, respectively. Ta incorporation modifies the local B–O coordination environment while preserving the underlying corner-sharing perovskite framework.

The parent CaSiO_3_ phase was simulated in the high-symmetry cubic perovskite structure, corresponding to the *Pm*3̄*m* space group (No. 221), where the structure consists of a three-dimensional framework of corner-sharing SiO_6_ octahedra and Ca^2+^ cations in the cuboctahedral A-site environment.^52^ The lattice parameters obtained from the structure file the present work, in the primitive (1 × 1 × 1) cell, are *a* = *b* = *c* = 6.818064 Bohr (≈3.607 Å), and *α* = *β* = *γ* = 90°. The optimized lattice parameter is in agreement with reported experimental and first-principles values. In the case of cubic CaSiO_3_, and other ABO_3_ perovskites, it verifies the validity of the current structure optimization.^53^ The *Pm*3̄*m* parent structure provides the crystallographic reference from which the Ta-substituted supercells were constructed. The atomic structure follows the conventional ABO_3_ perovskite configuration, where Ca is at the body-centered position (0.5,0.5,0.5) with a multiplicity of 1, Si at the corner position (0,0,0) also with a multiplicity of 1 and O at three symmetry-equivalent positions, namely (0,0.5,0), (0,0,0.5) and (0.5,0,0) with a multiplicity of 3. The structure file also indicates that Ca, Si and O atoms are characterized by muffin-tin radii of 2.2800, 1.4900 and 1.7300 Bohr, respectively. Moreover, the oxygen sublattice is associated with a nontrivial local rotation matrix, reflecting the equivalent orientation of the anion environment within the cubic lattice. The structure file contains 48 symmetry operations, confirming the high crystallographic symmetry of the parent phase. This ideal cubic geometry makes the CaO_12_ and SiO_6_ polyhedra symmetry-equivalent throughout the unit cell and produces uniform Ca–O and Si–O bond lengths, as expected for an undistorted cubic perovskite, as shown in Fig. 1(a). The close agreement of these structural features with reported crystallographic data for cubic CaSiO_3_ supports the reliability of the adopted parent model.^52,53^ With the Ta-doped structures, a (2 × 2 × 2) supercell was applied to the CaSiO_3_ lattice to simulate partial replacement of Si by Ta at the B-site of the perovskite structure. The same (2 × 2 × 2) supercell, containing eight Ca atoms, eight B-site positions, and 24 O atoms, was used consistently for both the 12.5% and 25% Ta compositions. This common supercell construction permits a direct comparison of the concentration-dependent influence of Ta substitution without introducing a change in supercell size as an additional structural variable.

For the 12.5% Ta composition, the lattice parameters of the cubic-derived supercell are *a* = *b* = *c* = 13.636128 Bohr (≈7.214 Å), with interaxial angles of 90°, corresponding to *x* = 0.125 in CaSi_1−*x*_Ta_*x*_O_3_. In this supercell, eight Ca atoms occupy the A-site sublattice (MULT = 8), and one Ta atom replaces one of the eight Si atoms at the B-site (MULT = 1). The resulting composition is therefore Ca_8_Si_7_TaO_24_, equivalent to CaSi_0_._875_Ta_0_._125_O_3_, corresponding to 12.5% Ta and 87.5% Si occupancy of the B-site sublattice.

The remaining Si atoms are split into three inequivalent groups with multiplicities of 3, 3, and 1, reflecting the chemically inequivalent environments created by Ta incorporation. The oxygen sublattice is accordingly classified into three different groups, O1, O2, and O3, with multiplicities of 6, 12, and 6, respectively. These oxygen atoms exhibit different local rotation matrices, indicating variations in their local coordination environments while preserving the overall corner-sharing perovskite framework. Such chemically induced local rearrangements are commonly observed in substituted oxide perovskites, where B-site substitution modifies the local octahedral environment without necessarily requiring a global crystallographic phase transformation.^54,55^

Previous studies have shown that oxide perovskites can undergo localized distortions when Ta substitutes at the B-site octahedral position, leading to modification of the local coordination environment without destroying the overall perovskite framework.^22^ However, the BO_6_ octahedra remain corner-sharing, and the Ca atoms maintain their characteristic A-site coordination environment, indicating that Ta incorporation does not disrupt the fundamental perovskite topology. Thus, at 12.5% Ta substitution, the structural response is characterized primarily by the creation of chemically inequivalent B–O environments and local coordination rearrangement, rather than by evidence for a uniform strengthening of individual Ta–O bonds. Fig. 1(b) shows the 12.5% Ta-doped structure. For the 25% Ta composition, the same (2 × 2 × 2) supercell was retained and two of the eight Si B-site atoms were replaced by Ta. The resulting explicit supercell composition is Ca_8_Si_6_Ta_2_O_24_, equivalent to CaSi_0_._75_Ta_0_._25_O_3_; thus, 25% of the B-sites are occupied by Ta and the remaining 75% by Si. This structural model is directly related to the 12.5% configuration, with the number of Ta-for-Si substitutions increasing from one to two while the supercell size and total number of B-sites remain unchanged. A comparable concentration-dependent supercell approach has been employed in first-principles investigations of Ta-substituted SrTiO_3_, including 12.5% and 25% substitution levels.^54^

Accordingly, the 25% system is described here using the actual (2 × 2 × 2), two-Ta substitutional model rather than the previously stated smaller *P*4/*mmm* tetragonal cell. Because the precise number of symmetry operations and the multiplicities of inequivalent Si, Ta, and O positions depend on the relaxed two-Ta arrangement, these quantities should be taken directly from the corresponding optimized structure output rather than inferred from a different crystallographic model. Only symmetry information explicitly supported by the corresponding relaxed structural output is used in the present discussion.

At the higher Ta concentration, the presence of two Ta-centered coordination environments increases the chemical in equivalence of the B–O network and may permit additional local relaxation relative to the 12.5% structure. The resulting changes are therefore described conservatively as concentration-dependent local structural modifications; no global symmetry reduction is assigned unless it is explicitly supported by the relaxed crystallographic output. Within the formal ionic picture, Ta-for-Si substitution is treated as an aliovalent replacement of nominal Si^4+^ by nominal Ta^5+^, corresponding to a one-electron valence difference per substituted Ta site. These oxidation-state labels are used only for formal electron counting and are not imposed as fixed ionic charges in the DFT calculation; in particular, “Ta^5+^” should not be interpreted as a quantitatively determined +5 atomic charge.

In the present stoichiometric supercell calculations, no oxygen vacancies, cation vacancies, interstitials, or other compensating point defects were introduced, and no net supercell charge was imposed. The periodic DFT cells therefore remain globally charge neutral, while the additional electronic population associated with the changed atomic composition is redistributed self-consistently among the available electronic states. Possible compensation by native defects under experimental growth conditions is not assumed here; establishing such mechanisms would require explicit defect-formation-energy calculations as functions of chemical potential and Fermi level.^56^

This treatment defines the present structures as stoichiometric, defect-free Ta-for-Si substitution models and distinguishes the formal oxidation-state bookkeeping from a direct charge-partitioning determination of the Ta oxidation state. It also separates the modeled electronic-compensation limit from the more complex defect equilibria that may occur experimentally. These local structural modifications do not disrupt the overall perovskite framework, as the corner-sharing CaO_12_ and SiO_6_/TaO_6_ polyhedra are preserved throughout the modeled structures. Fig. 1(c) shows the 25% Ta-doped configuration. Overall, these crystallographic results show that Ta incorporation at 12.5% and 25% does not destroy the basic structural integrity of the CaSiO_3_ perovskite framework; instead, it modifies the local B-site coordination environment around the substituted atoms. The three compositions are therefore consistently represented throughout the present work as pristine CaSiO_3_, CaSi_0_._875_Ta_0_._125_O_3_ (12.5% Ta), and CaSi_0_._75_Ta_0_._25_O_3_ (25% Ta).

These structural results define the first component of the structure–bonding–property relationship considered in this work: Ta-for-Si substitution produces systematic lattice expansion, chemically inequivalent B–O coordination environments, and concentration-dependent local relaxation while preserving the corner-sharing framework. The corresponding bonding response is evaluated independently in Section 3.4.5 using COHP and ELF, whereas the mechanical response is quantified in Section 3.7 through the calculated elastic constants and derived moduli. Accordingly, the mechanical trends are correlated with the combined structural and electronic reorganization of the B–O network, rather than being used as indirect evidence for a monotonic increase in Ta–O covalent bond strength. In addition, the FP-LAPW method used in this work, in which the crystal is divided into atom-centered muffin-tin spheres and an interstitial region, allows an accurate description of both equivalent and inequivalent atomic environments as well as dopant-induced changes in all three compositions. This confirms that the adopted computational framework is suitable for investigating the electronic structure and functional behavior of Ta-doped CaSiO_3_ systems.^39,41^

**Fig. 2:**
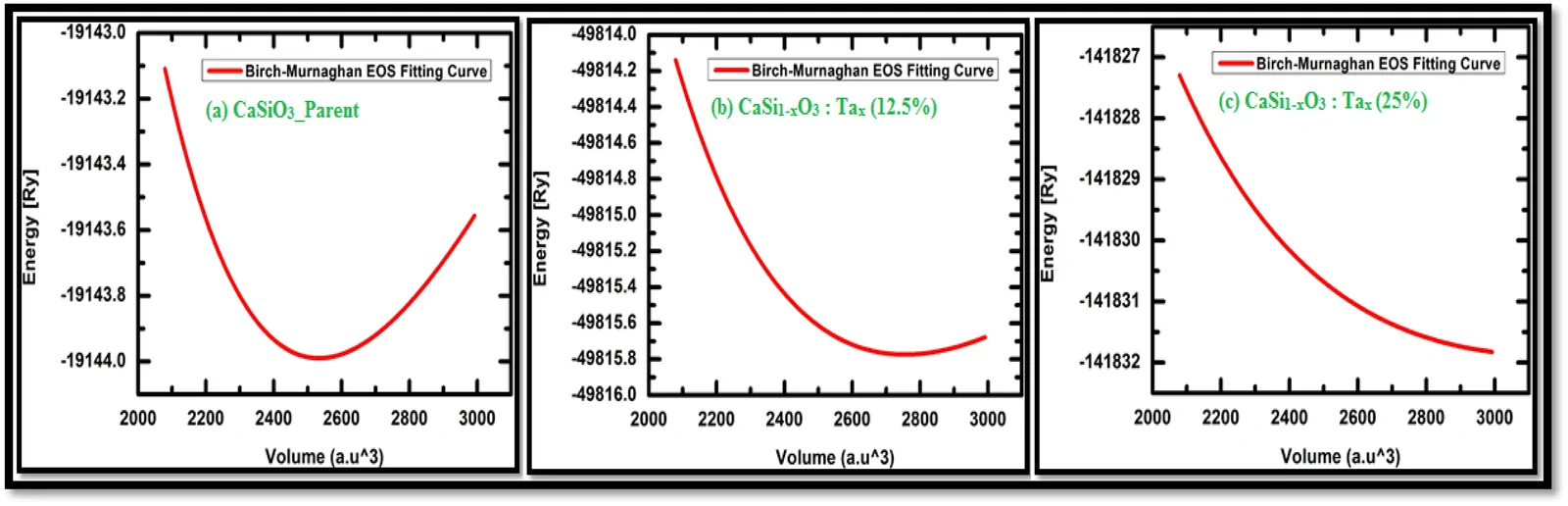
Birch–Murnaghan fitted energy–volume (*E*–*V*) curves for (a) pristine CaSiO_3_, (b) CaSi_1−*x*_Ta_*x*_O_3_ (*x* = 12.5%), and (c) CaSi_1−*x*_Ta_*x*_O_3_ (*x* = 25%). The well-defined energy minima confirm the structural stability of the optimized systems, while the Ta-induced changes in equilibrium volume and curve curvature indicate lattice expansion and lower EOS-derived stiffness relative to pristine CaSiO_3_, with a slight recovery in *B*_0_ from 12.5% to 25% Ta.

Fig. 2(a–c), illustrates the dependence of total energy on unit-cell volume for pristine CaSiO_3_ and Ta-doped CaSi_1−*x*_Ta_*x*_O_3_ systems at *x* = 12.5% and 25%. These energy-volume trends describe the response of each structure to systematic lattice compression and expansion, providing insight into their relative structural stability and equilibrium behavior. For each composition, a series of trial volumes was generated from the optimized structure, and the corresponding total energies were calculated to obtain the complete equation-of-state (EOS) profile. The resulting energy-volume data were fitted using the third-order Birch–Murnaghan equation of state to determine the equilibrium volume (*V*_0_), minimum total energy (*E*_0_), bulk modulus (*B*_0_), and its pressure derivative 
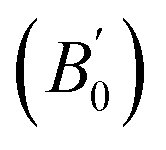
. This formalism provides a consistent thermodynamic description of the structural response of the system under hydrostatic deformation.^57^ The equilibrium parameters obtained for pristine CaSiO_3_ are in good agreement with reported experimental and theoretical values for cubic perovskite oxides, confirming the reliability of the present EOS fitting procedure.^58^ The well-defined minima in the energy–volume curves indicate that pristine CaSiO_3_ and both Ta-substituted compositions possess stable equilibrium configurations within the investigated volume range.

A systematic increase in the equilibrium volume, accompanied by a change in EOS-curve curvature, is observed with increasing Ta concentration, which can be attributed to the differences in ionic size and local chemical environment between the Si- and Ta-containing B-sites. This mismatch produces local structural relaxation and coordination rearrangement; its consequences for chemical bonding and mechanical behavior are evaluated separately through the COHP/ELF and elastic analyses rather than being inferred from the volume expansion itself. However, the absence of sharp changes or anomalous features in the energy–volume profiles indicates that Ta incorporation does not produce an evident structural instability within the investigated EOS volume range. Overall, the combined EOS analysis and FP-LAPW calculations provide a detailed evaluation of the structural, energetic, and elastic behavior of CaSiO_3_ and its Ta-substituted analogs, offering clear insight into the influence of Ta doping on lattice stability and mechanical response.1
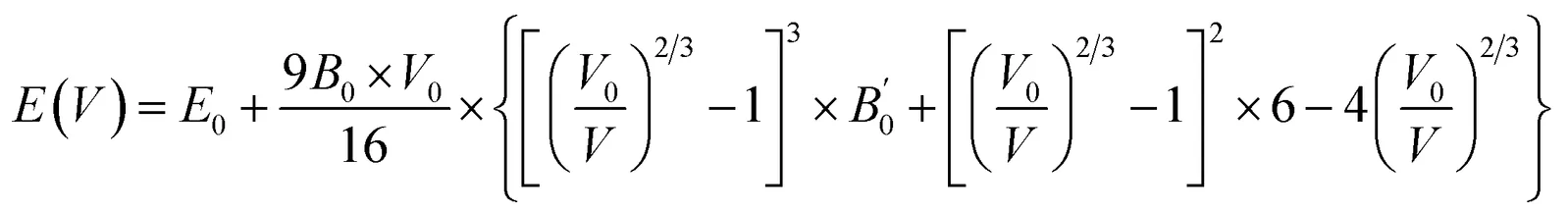
2
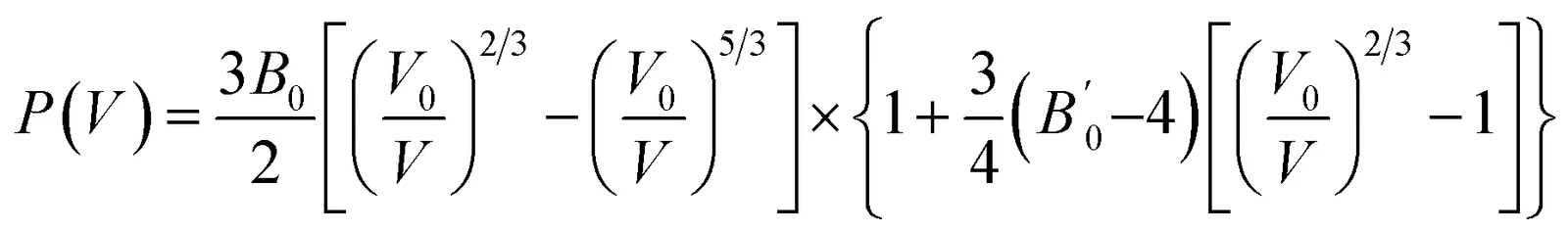


Fig. 2(a–c), shows the energy–volume curves of pristine CaSiO_3_ and its Ta-doped analogs CaSi_1−*x*_Ta_*x*_O_3_ (*x* = 12.5% and 25%). The minimum point of every of the (*E*–*V*) curves is the equilibrium volume (*V*_0_), which is the most energetically stable state following complete structural relaxation. Any compression or expansion away from the equilibrium volume increases the total energy, reflecting the energetic cost required to deform the lattice. Under compression, the interatomic distances decrease, leading to greater overlap of electronic charge densities and stronger Pauli repulsion between atomic cores. In contrast, lattice expansion increases the interatomic separation and modifies the balance of interatomic interactions within the perovskite framework. These competing effects produce the smooth *E*–*V* profiles observed for pristine CaSiO_3_ and the 12.5% and 25% Ta-doped systems. The smooth and continuous nature of these curves together with their well-defined minima indicates stable equilibrium configurations within the investigated volume range, without evidence of an abrupt structural instability during the EOS optimization.

For crystalline CaSiO_3_ [Fig. 2(a)], the equilibrium volume corresponds to the lowest point of the energy–volume (*E*–*V*) curve, indicating a highly ordered cubic perovskite structure composed of corner-sharing SiO_6_ octahedra. When Si is partially substituted by Ta at 12.5% concentration [Fig. 2(b)], the equilibrium volume shifts to a higher value, indicating Ta-induced lattice expansion. This expansion is associated with the different local size and chemical environment introduced by Ta substitution and the consequent relaxation of the surrounding oxygen framework. With a further increase in Ta concentration to 25% [Fig. 2(c)], the equilibrium volume increases further, while the *E*–*V* curve shows a noticeable change in curvature. This behavior reflects enhanced compressibility and a corresponding decrease in bulk modulus compared with the undoped structure. Such trends are typical of aliovalent B-site substitution in oxide perovskites, where dopant incorporation modifies the local coordination environment without necessarily disrupting the basic perovskite topology.^59^ The equilibrium-volume trend therefore increases from pristine CaSiO_3_ to the 12.5% and 25% Ta-doped systems, whereas the corresponding change in stiffness must be interpreted directly from the fitted *B*_0_ values rather than assumed to decrease monotonically with Ta concentration.

The direct information that the curvature of the Birch Murnaghan equation-of-state (EOS) fitting would give would be the mechanical response of the system; a stiffer lattice would have a steeper curvature and a more compressible one would have a broader minimum. With the (*E*–*V*) data computed, the important equilibrium parameters such as the equilibrium volume (*V*_0_), bulk modulus (*B*_0_), its derivative with pressure 
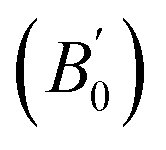
 and the minimum total energy (*E*_0_) have been obtained at all compositions by fitting the data to the third-order Birch Murnaghan EOS. The values presented in Table 1 show a systematic variation in the structural and elastic parameters with increasing Ta concentration. Pristine CaSiO_3_ exhibits the highest bulk modulus, which is characteristic of a rigid and highly symmetric perovskite framework formed by well-connected SiO_6_ octahedra. Upon Ta substitution, *B*_0_ decreases markedly from 215.3005 GPa for pristine CaSiO_3_ to 167.1973 GPa at 12.5% Ta and then increases slightly to 169.5482 GPa at 25% Ta. Therefore, both Ta-doped compositions are considerably more compressible than pristine CaSiO_3_, but the stiffness change between 12.5% and 25% Ta is small and is not strictly monotonic.

**Table 1:** Parameters derived with Birch Murnaghan EOS (*V*_0_, *E*_0_, *B*_0_ and 
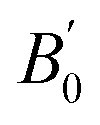
) of pristine and Ta-doped CaSiO_3_, showing systematic lattice expansion and a non-monotonic EOS-derived stiffness response with Ta concentration

Materials	*V*_0_ (Bohr^3^)	*E*_0_ (Ry)	*B*_0_ (GPa)	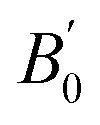
CaSiO_3__(parent)	2533.4554	−19143.990180	215.3005	5.000
CaSi_1−*x*_Ta_*x*_O_3_ (*x* = 12.5%)	2755.1025	−49815.775160	167.1973	5.000
CaSi_1−*x*_Ta_*x*_O_3_ (*x* = 25%)	3155.7362	−141831.872873	169.5482	5.000

The combined analysis of Fig. 2 and Table 1 therefore confirms that Ta doping preserves the structural stability of CaSiO_3_ while enabling controlled tuning of its mechanical response. The EOS-derived stiffness follows the order pristine CaSiO_3_ > 25% Ta > 12.5% Ta. The EOS-derived bulk modulus discussed here is obtained from the curvature of the hydrostatic energy–volume relation and should be distinguished from the bulk modulus derived later from the elastic constants, because the two quantities originate from different computational analyses. Because the two approaches yield different numerical trends, they are discussed separately and are not combined into a single monotonic stiffness claim. This distinction is also essential to the structure–bonding–property interpretation: neither the EOS curvature nor the elastic constants are treated as direct measures of Ta–O covalency; instead, the mechanical response is interpreted jointly with the independently calculated structural and COHP/ELF descriptors.

### 3.2. Dynamical stability, phonon dispersion

**Fig. 3:**
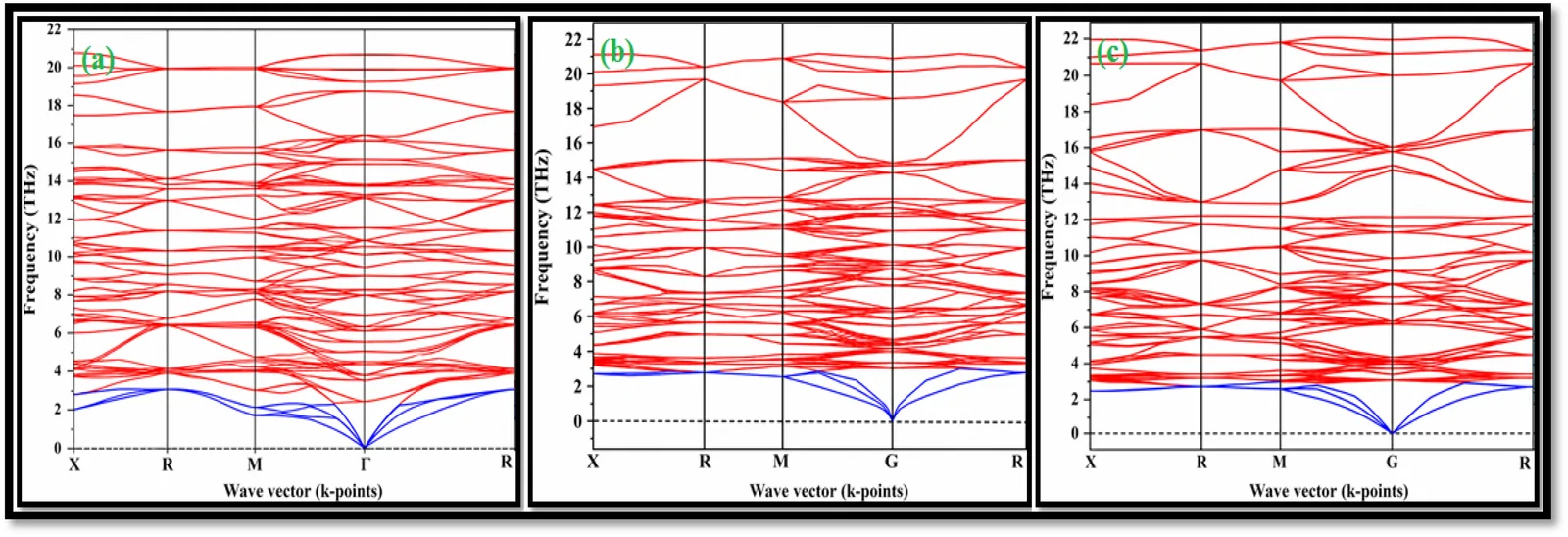
Phonon dispersion spectra of (a) pristine CaSiO_3_, (b) CaSi_1−*x*_ Ta_*x*_O_3_ at *x* = 12.5%, and (c) CaSi_1−*x*_Ta_*x*_O_3_ at *x* = 25%. The absence of imaginary phonon branches confirms the dynamical stability of all optimized structures. The progressive modification of the low-frequency branches after Ta substitution reflects the heavier Ta mass and local Ta–O-induced lattice distortion.

The dynamical stability of the pristine CaSiO_3_ and the Ta-substituted CaSi_1−*x*_ Ta_*x*_O_3_ (*x* = 12.5% and 25%) was thus evaluated through the calculated phonon dispersion spectra which are illustrated in Fig. 3. This check is crucial because the substitution of Si by the heavier and higher-valent Ta atom would lead to local octahedral distortion and symmetry lowering, especially at the higher substitution level. The phonon branches of the parent CaSiO_3_ system [Fig. 3(a)] are all positive over the range of high-symmetry points considered, and the modes have low frequencies at *Γ*. This validates that there are no imaginary vibrational modes, thus supporting the dynamical stability of the optimized CaSiO_3_ structure. This result is in good agreement with recent first-principles lattice-dynamics calculations of CaSiO_3_ phases using phonon-based methods to assess the stability and phonon dynamics of the phases.^60^ Even after 12.5% Ta substitution [Fig. 3(b)], the phonon spectrum does not exhibit any imaginary frequencies, indicating that substitution of isolated Ta at the Si site does not destabilize the perovskite network. The redistribution of low-frequency branches is physically reasonable and can be related to the bigger mass of Ta–O coordination and local relaxation around it. Recent studies on Ta-substituted oxide perovskites also indicate that the local structural and electronic environment can be altered with the incorporation of Ta without destroying the perovskite-type framework.^61^ The phonon branches at [Fig. 3(c)] of 25% Ta concentration are also positive, indicating the stronger local distortion at the higher Ta concentration is also dynamically tolerable. The low frequency regime is slightly more modified than the parent system, corresponding to higher mass contrast and greater lattice perturbation caused by Ta–O interactions. Based on the usual phonon stability criterion, the lack of imaginary branches of the phonon spectra confirms that the optimized structures are dynamically stable.^62^ The phonon results, combined with the well-defined EOS minima and the negative formation energies, thus give a consistent picture of structural stability for all compositions investigated.

### 3.3. Formation energy analysis

Formation energy is a key thermodynamic parameter used to evaluate the stability of crystalline materials and their likelihood of formation under equilibrium conditions. In first-principles calculations, it represents the energy change associated with forming a compound from its constituent elements in their reference states. A negative formation energy indicates that the compound is thermodynamically more stable than the separated elemental phases. Moreover, a more negative value generally reflects a stronger driving force for compound formation and greater structural stability.^57,58^

In the case of the pure CaSiO_3_ compound and its Ta-substituted counterparts in CaSi_1−*x*_Ta_*x*_O_3_ (*x* = 0.125 and 0.25), where Ta replaces Si in concentrations of 12.5% and 25%, the value of the formation energy per atom was determined by the ordinary thermodynamic relation.3
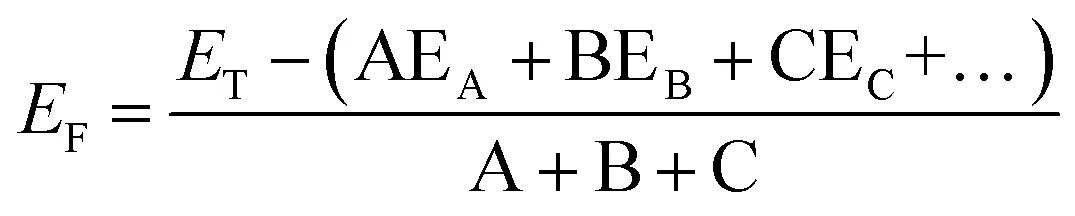


Oxide perovskites are commonly examined using first-principles calculations, where negative formation energies indicate stable parent lattices. A slight reduction in the magnitude of formation energy after aliovalent B-site substitution generally suggests that the doped systems remain thermodynamically feasible and structurally stable.^63^ Formation energy in the current research of pristine CaSiO_3_ and Ta-substituted compositions, CaSi_1−*x*_Ta_*x*_O_3_ (*x* = 0.125 and 0.25) was compared with Ta substituting Si at concentrations of 12.5% and 25%. In this case, *E*_F_ is the energy of formation per atom, *E*_T_ is the total energy of the optimized structure and *E*_A_, *E*_B_, *E*_C_, *etc.*, are the energies of the respective elements (Ca, Si, O, and Ta) in their respective ground-state reference states. A, B, C *etc.* are the coefficients representing the number of atoms of each species in the considered formula unit or supercell. The formulation provides a thermodynamic comparison of the undoped and Ta-doped structures that is consistent.

Table 2 shows that the formation energies of all three compositions are negative, indicating that pristine and Ta-substituted CaSiO_3_ systems are thermodynamically stable. The parent CaSiO_3_ phase exhibits the most negative formation energy, reflecting the higher intrinsic stability of the undoped lattice. With Ta substitution at the Si site, the magnitude of the formation energy gradually decreases, suggesting that dopant incorporation introduces a certain degree of energetic perturbation into the host framework. This behavior can be associated with local structural relaxation arising from the ionic-size, bonding-character, and valence-state differences between Ta and Si. Nevertheless, the formation energies of both Ta-doped compositions remain significantly negative, confirming that Ta incorporation does not eliminate the thermodynamic stability of the perovskite framework. In interpreting this trend, the Ta oxidation state is treated only within a formal ionic picture; the formation-energy data do not constitute a quantitative determination of the atomic charge on Ta.

**Table 2:** Formation energies (eV per atom) of pure CaSiO_3_ and Ta-substituted CaSi_1−*x*_Ta_*x*_O_3_ (*x* = 12.5% and 25%), which show the impact of Ta incorporation on the thermodynamic stability and energetic properties of the perovskite framework

Materials	Formation energy (eV per atom)
CaSiO_3__(parental)	−4.11
CaSi_1−*x*_Ta_*x*_O_3_ (*x* = 12.5%)	−3.28
CaSi_1−*x*_Ta_*x*_O_3_ (*x* = 25%)	−2.96

These trends are well consistent with previous reports on lanthanide- and transition-metal-modified perovskite systems, such as lanthanide-doped CsPbCl_3_,^64,65^ Eu ^3+^/K^+^ co-doped Ca_3_WO_6_,^66^ and Eu^2+^-doped CsPbBr_3_.^67^ These systems typically form dopants which, when formed, cause a slight reduction in the strength of the formation energy, although has no effect on the overall thermodynamic stability of the host lattice.

The present results indicate that Ta incorporation in CaSiO_3_ does not disrupt the stability of the perovskite framework. Although the magnitude of the formation energy slightly decreases with increasing Ta content, all calculated formation energies remain negative, confirming the thermodynamic feasibility of the investigated compositions. Within the present elemental-reference formation-energy framework, this result supports the energetic viability of the modeled Ta-substituted structures, but it is not by itself a quantitative measure of Ta oxidation state or equilibrium defect chemistry. Similar trends have also been reported in transition-metal-doped oxide perovskites, where dopant incorporation causes a moderate reduction in formation-energy magnitude without destabilizing the perovskite lattice.^68^ The order of energy of formation is as follows: CaSiO_3_ → CaSi_1−*x*_Ta_*x*_O_3_ (12.5%) → CaSi_1−*x*_Ta_*x*_O_3_ (25%), gradual decrease in thermodynamic stability as the concentration of Ta increases. In a formal ionic description, Ta-for-Si substitution may be represented as nominal Si^4+^ → nominal Ta^5+^ substitution. These oxidation-state labels are used only for formal electron counting and should not be interpreted as Bader-derived or otherwise quantitatively calculated atomic charges. The observed formation-energy trend is therefore discussed conservatively in terms of the altered local chemical environment and accompanying lattice relaxation introduced by Ta substitution. Despite these systematic changes, the formation energy remains negative at all doping levels, showing that the modeled Ta-substituted phases remain energetically stable relative to the chosen elemental reference states; this does not, on its own, establish the thermodynamic preference of Ta incorporation under all experimental growth conditions. This behavior is consistent with oxide perovskites, where the introduction of transition-metal dopants often leads to a moderate decrease in formation-energy magnitude without destabilizing the host lattice. Accordingly, the formation-energy results support further investigation of Ta-substituted CaSiO_3_ while avoiding application-level claims that are not established by formation energy alone.

### 3.4. Electronic structure and emergent band features

#### 3.4.1. Band structure evolution and electronic dispersion

**Fig. 4:**
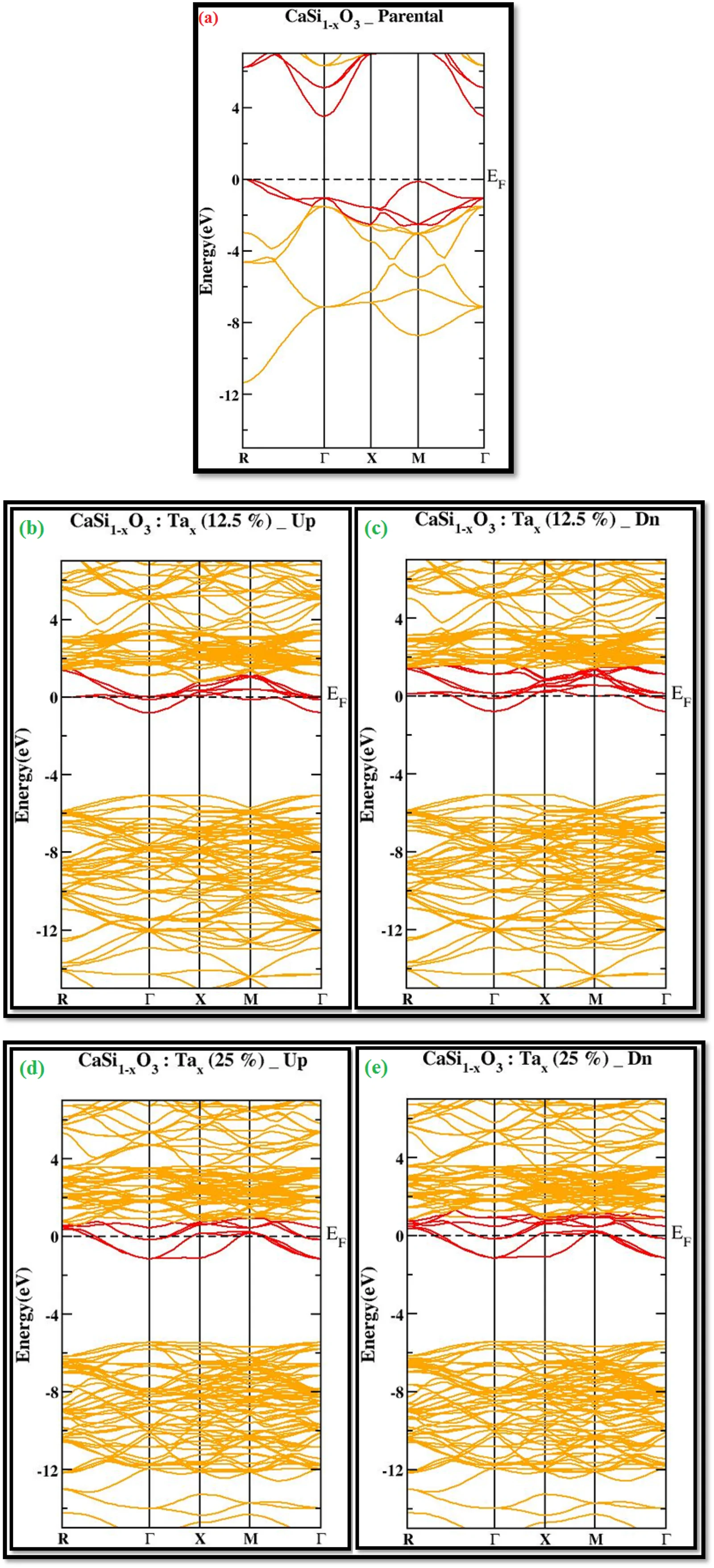
Electronic structures of pristine CaSiO_3_ (a) and Ta-doped CaSi_1−*x*_Ta_*x*_O_3_ at 12.5% (b and c) and 25% (d and e) substitution, displayed for the two spin channels. The yellow curves show the calculated electronic bands, whereas the red curves are used solely to visually identify the bands that intersect the Fermi level (*E*_F_); they do not represent atom- or orbital-projected states. Pristine CaSiO_3_ retains a clear band gap, while Ta-induced states appear near *E*_F_ at 12.5% substitution and cross *E*_F_ at 25%, indicating a gradual evolution from wide-gap semiconducting behavior to a near-metallic and ultimately metallic state.

The electronic band structure provides a fundamental basis for understanding the intrinsic electronic behavior of crystalline materials, because it governs charge transport, carrier excitation, and optical transitions in functional oxides.^69,70^Fig. 4(a–e), shows the band structures of pristine CaSiO_3_ and Ta-substituted CaSi_1−*x*_Ta_*x*_O_3_ at *x* = 0.125 and 0.25, calculated using the GGA+*U* method. The Hubbard *U* correction is important in these systems because it helps describe the localized Ta-derived 5d states and improves the separation between occupied and unoccupied electronic states. As a result, the GGA+*U* approach provides a more reliable description of the band dispersion, band-gap evolution, and dopant-induced electronic reconstruction. In pristine CaSiO_3_, the Fermi level lies within a clear band gap, confirming its wide-band-gap semiconducting nature. After Ta substitution, additional Ta-5d-derived bands appear near the Fermi level and hybridize with O-2p states, producing donor-like electronic states close to the conduction-band edge. At 12.5% Ta, the donor-derived states lie at or very near *E*_F_ and, together with the small Fermi-surface pockets discussed in Section 3.4.3, identify a degenerate n-type/near-metallic regime; at 25% Ta, the Ta-derived bands cross *E*_F_, establishing a metallic state. The spin-up and spin-down band structures of the doped systems show broadly similar features, suggesting that the electronic reconstruction is mainly governed by Ta-5d/O-2p hybridization rather than strong spin splitting. Similar behavior has been reported in related doped perovskites, such as Nb-doped SrTiO_3_, where dopant-derived d states appear near the conduction-band minimum, reduce the band gap, and modify carrier transport.^53,71^ Therefore, the present band structures, interpreted together with the DOS and Fermi-surface results, establish a concentration-driven evolution from a wide-gap semiconductor (pristine CaSiO_3_) to a degenerate donor-doped/near-metallic state (12.5% Ta) and finally to a metallic state (25% Ta). The electronic reconstruction produced by Ta substitution should be interpreted within the defect-free substitution model defined in Section 2. In the formal ionic picture, replacement of Si^4+^ by nominal Ta^5+^ represents a donor-type aliovalent substitution corresponding to one additional electron per substituted Ta site. No oxygen vacancy, cation vacancy, interstitial, or other explicit compensating point defect was introduced in the calculated structures. The periodic DFT supercells were treated as overall charge-neutral systems, and the nominal Ta^5+^ and Si^4+^ labels were not imposed as fixed atomic charges. Instead, the electronic density was allowed to redistribute self-consistently according to the available states of the substituted lattice.

Within this model, the donor-associated electronic charge is accommodated in the electronic states that emerge close to the conduction-band edge and the Fermi level. Their predominantly Ta-5d character, together with contributions from neighboring O-2p states, is examined further by the orbital-resolved DOS analysis in Section 3.4.2. Thus, the present calculations represent the defect-free electronic-compensation limit of Ta substitution rather than an explicit defect-compensated configuration. Intrinsic defect-mediated compensation may occur under particular experimental growth conditions; however, identifying the thermodynamically preferred compensating defects would require dedicated defect-formation-energy calculations as functions of chemical potential and Fermi level and is therefore not inferred from the present stoichiometric substitution model. Fig. 4(a), shows the electronic band structure of pristine CaSiO_3_, which exhibits a wide-band-gap semiconducting character. The calculated band gap is indirect, with the valence-band maximum (VBM) located near the *R* point and the conduction-band minimum (CBM) located near the *Γ* point. This indirect transition is typical of silicate-based perovskites and originates from the electronic separation between O-2p-dominated valence states and Si-derived antibonding conduction states. The parent compound is therefore classified as a wide-band-gap semiconductor with an intrinsically insulating carrier limit, as confirmed by the calculated band gap of 3.432 eV. The noticeable curvature of the valence and conduction bands near the band edges suggests relatively dispersive states, which can support carrier mobility once carriers are thermally or optically excited. Minor differences between the present band gap and previously reported theoretical values may arise from variations in structural relaxation, octahedral distortion, exchange–correlation treatment, and electron-correlation parameters.^72^ Overall, the electronic structure of pristine CaSiO_3_ shows a clear separation between valence and conduction bands, reflecting an intact SiO_6_ octahedral network and the absence of dopant-induced states near the Fermi level.

The band structures of CaSi_1−*x*_Ta_*x*_O_3_ at 12.5% substitution of Ta, shown in Fig. 4(b) (spin-up) and Fig. 4(c) (spin-down) show significant changes in the electronic structure of the pure compound. Introduction of Ta will cause Ta-derived d states to appear around the conduction band region which will interact with the O-2p and other nearby states, thus creating other electronic states near the Fermi level. Importantly, the electronic character of this composition should not be described as that of an ordinary narrow-gap semiconductor. The appearance of partially occupied Ta-derived states at (*E*_F_), together with the small Fermi-surface pockets identified in Section 3.4.3, indicates a degenerate donor-doped n-type regime with incipient metallic character. Thus, although the underlying host valence–conduction separation is reduced relative to pristine CaSiO_3_, the relevant electronic state at the Fermi level is characterized by finite carrier occupation rather than purely thermally activated excitation across a conventional semiconductor gap. This behavior is consistent with previous reports of transition-metal-modified oxide perovskites, in which donor-derived states modify band dispersion and generate electronically active states near the conduction-band edge.^73,74^ For the 12.5% Ta composition, the appearance of Ta-derived states close to *E*_F_ provides direct electronic-structure evidence for accommodation of the nominal donor electron within the calculated electronic system rather than through an explicitly introduced structural defect. The present band structure therefore reflects electronic compensation within the defect-free substitution model. When interpreted together with the small Fermi-surface pockets reported for this composition, these near-*E*_F_ states are more consistently described as a degenerate n-type/near-metallic electronic regime rather than as a conventional narrow-gap semiconductor. A comparison of the spin-up and spin-down band structures shows that the electronic states have nearly identical distributions, indicating the absence of significant spin splitting and confirming the weakly magnetic or nearly non-magnetic nature of the Ta-substituted system. Moreover, the reduced band dispersion near the conduction-band edge suggests an increase in carrier effective mass and partial localization of electronic states within the Ta-centered octahedral units. The calculated reduction in the host-band separation and the emergence of Ta-derived states near *E*_F_ demonstrate a concentration-dependent reconstruction of the electronic structure. These results define the intrinsic electronic response of the substituted system, whereas assessment of specific optoelectronic or energy-related performance requires additional application-oriented calculations and experimental validation.

Multiphasic change in the electronic structure is very apparent in the band structures of CaSi_1−*x*_Ta_*x*_O_3_ at 25% Ta substitution as in Fig. 4(d), (spin-up) and Fig. 4(e), (spin-down) when compared to lower doping concentrations. With increasing Ta content, the contribution of Ta-derived 5d states becomes more pronounced, strongly overlapping with the conduction band and extending toward the Fermi level Consequently, the band gap is substantially reduced, and the system evolves toward a metallic state because Ta-derived bands cross *E*_F_ and the transport-relevant electronic gap at the Fermi level closes. The increased density of electronic states near the Fermi level indicates the formation of extended conduction pathways, which originate from the stronger participation of Ta-5d orbitals in the electronic structure. This behavior reflects a transition toward more delocalized carrier transport and is consistent with the emergence of itinerant d-electron character. At 25% Ta substitution, the increased number and dispersion of Ta-derived states near and across *E*_F_ indicate stronger delocalization of the donor-associated electronic charge. This concentration-dependent evolution is consistent with the electronic-compensation picture adopted for the present defect-free supercells and with the increased carrier population examined later through the Fermi-surface and nominal carrier-concentration analyses. The spin-up and spin-down band structures show nearly similar features, indicating weak spin splitting and limited spin polarization even at higher Ta concentration. Despite these electronic changes, the overall band dispersion remains continuous, suggesting that the basic CaSiO_3_ lattice framework is preserved, while Ta-derived states dominate the electronic behavior around the Fermi level. This evolution from wide-band-gap semiconducting behavior to a metallic state with increasing dopant concentration is consistent with dopant-induced electronic reconstruction commonly observed in transition-metal-doped oxide perovskites.^75,76^ Density-functional studies of related Ta-substituted perovskites, such as Ta-doped Sr-based systems, also report gradual band-structure modification and enhanced carrier delocalization with increasing Ta concentration.

This trend agrees well with the semiconductor-to-metal transition observed in CaSi_1−*x*_Ta_*x*_O_3_.^77^

The electronic transitions can be observed to have originated due to the following factors:

➢ The accumulation of Ta-5d electronic contributions in the region near the Fermi level (*E*_F_)

➢ Change in conduction-band dispersion giving much flatter dispersion bands and higher carrier effective masses.

➢ More continuous and delocalized electronic conduction pathways were formed as Ta concentration increased.

➢ Local structural changes caused by Ta replacement of the Si site, which leads to distortion of the SiO_6_/TaO_6_ octahedral structure and changed bonding properties.

The calculated electronic structures instead reveal two concentration-dependent regimes: the 12.5% Ta composition exhibits degenerate donor-doped/near-metallic behavior, whereas the 25% Ta composition shows clear Fermi-level band crossing and metallic electronic character. These classifications are based directly on the calculated electronic structure and are not used to assign conductive-oxide, thermoelectric, or other device-level functionality that has not been explicitly evaluated in the present study. The observed electronic evolution of CaSi_1−*x*_Ta_*x*_O_3_ is highly consistent with the trends reported for transition-metal-substituted perovskites. In moderately doped material including SrTi_1−*x*_Nb_*x*_O_3_, a semiconductor -metal transition may be driven by a moderate level of doping which is validated by experiments as well as first-principles calculations at *x* ≈ 0.20.^78–80^ A comparable semiconductor-to-metal evolution is obtained in the present system at 25% Ta, where the increased Ta-derived d-state contribution produces Fermi-level band crossing and metallic carrier transport. Likewise, Nb-doped BaSnO_3_ has been shown to have donor-induced narrowing observed in the band gap and higher conduction-band carrier density that lead to degenerate semiconducting properties and higher carrier mobility.^81,82^ The contributions of these d orbitals to the formation of conduction pathways due to hybridization with oxygen states are overall as observed. In CaSi_1−*x*_Ta_*x*_O_3_, Ta–O bond and the energetic location of Ta-5d states are similar in adjusting the electronic structure and charge transport. Moreover, the alkali-niobate perovskites, including KNbO_3_ and NaNbO_3_, have a wide band gap between 3.1 and 3.5 eV ^83,84^ this behavior is similar to the semiconducting nature of pristine CaSiO_3_. Comparable electronic changes have also been observed in doped NaNbO_3_ systems, where transition-metal-driven modifications can alter optical transitions and electronic states.^85^ These findings are consistent with the behavior of Ta-substituted CaSiO_3_, particularly at the lower doping concentration of 12.5%, where band-gap tuning evolves into a degenerate donor-doped/near-metallic electronic state.

As summarized in Table 3, the findings demonstrate that dopant-induced electronic-structure reconstruction occurs in CaSi_1−*x*_Ta_*x*_O_3_, where Ta substitution drives a consistent sequence from the wide-gap semiconducting parent phase to a degenerate n-type/near-metallic state at 12.5% Ta and a metallic state at 25% Ta through progressive occupation and eventual crossing of Ta-5d-derived states at *E*_F_. This classification should be applied consistently in the subsequent DOS, Fermi-surface, optical, and thermoelectric interpretations.

**Table 3:** Evolution of electronic structure and carrier transport in CaSi_1−*x*_Ta_*x*_O_3_, demonstrating systematic band-gap modulation and a concentration-driven progression from wide-gap semiconducting behavior to a degenerate donor-doped/near-metallic regime and finally to a metallic state

System	Band gap type	Band gap (eV)	VBM/CBM location	Spin character	Electronic nature	Dominant states	Carrier behavior	Key physical features
CaSiO_3_ _(parent)	Indirect (*R* → *Γ*)	3.432	VBM: *R*/CBM: *Γ*	Non-spin-polarized	Wide-gap semiconductor	O-2p (VB), Si-3p/3s (CB)	High dispersion → light carriers	Clear band separation; intact SiO_6_ octahedral network
CaSi_1−*x*_Ta_*x*_O_3_ (*x* = 12.5%)	Indirect (modified)	Reduced	Shift toward *Γ*	Spin-up ≈ spin-down	Degenerate n-type/near-metallic	Ta-5d^+^ O-2p	Reduced dispersion → partial localization	Ta-induced states at/near *E*_F_; onset of Fermi-level occupation
CaSi_1−*x*_Ta_*x*_O_3_ (*x* = 25%)	Gap closure/Fermi-level crossing	≈0	Ta-derived bands cross *E*_F_	Non-spin-polarized	Metallic	Strong Ta-5d at *E*_F_	Delocalized transport	Band overlap; continuous conduction channels

#### 3.4.2. Density of states and orbital hybridization characteristics

**Fig. 5:**
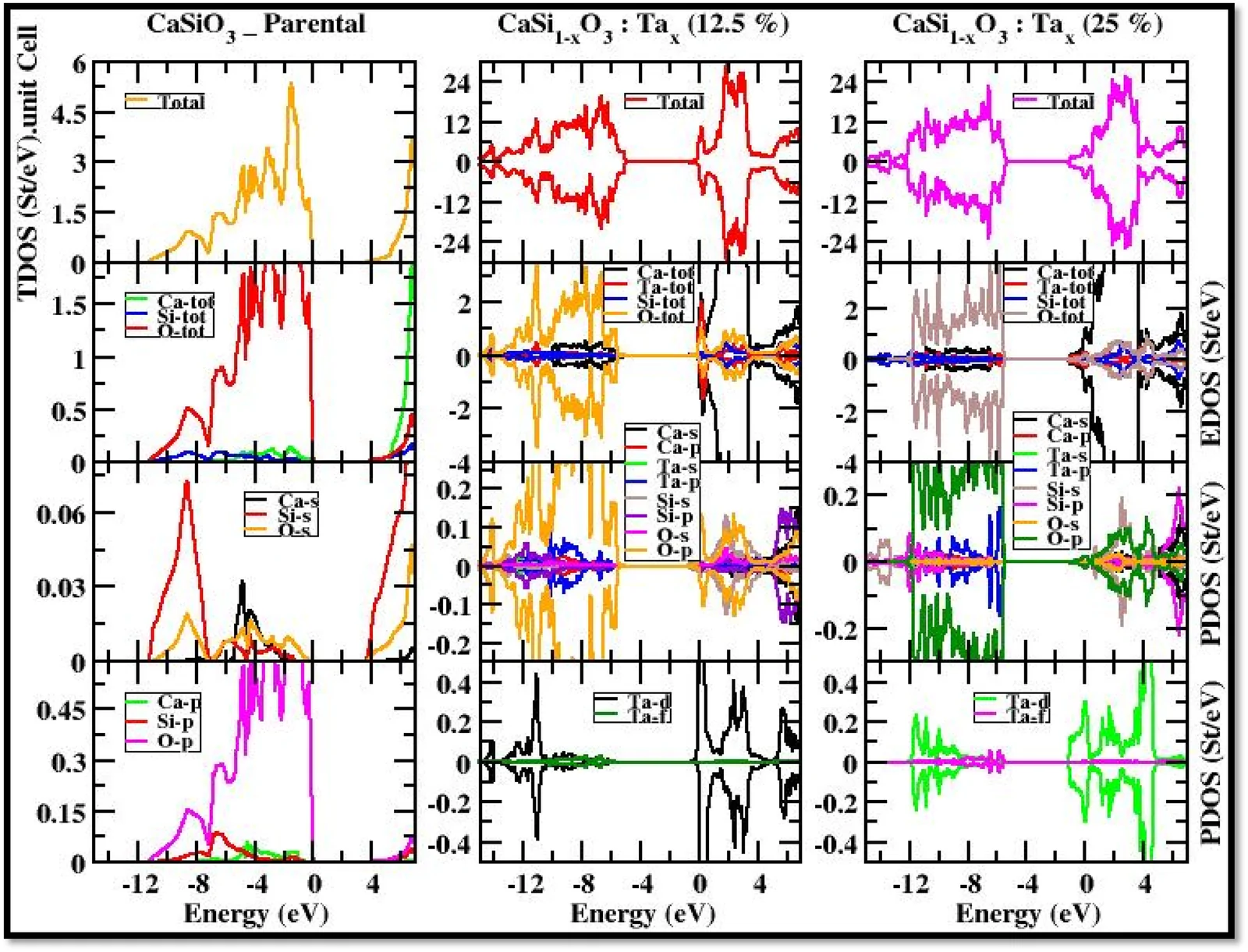
TDOS, EDOS, and spin-resolved PDOS of pristine CaSiO_3_ and Ta-substituted CaSi_1−*x*_Ta_*x*_O_3_ (*x* = 12.5% and 25%). For the Ta-substituted systems, the two spin channels are displayed on opposite sides of the zero-DOS axis. Ta-5d-derived states extend to the Fermi level at 12.5% Ta and cross *E*_F_ at 25% Ta. The finite spin-channel asymmetry associated predominantly with the Ta-5d/O-2p near-*E*_F_ states at 12.5% is strongly reduced at 25%, consistent with the concentration-dependent suppression of the calculated net magnetic moment. The DOS simultaneously shows the evolution from the wide-gap parent phase to a degenerate n-type/incipient metallic state at 12.5% Ta and a metallic state at 25% Ta.

Fig. 5, shows the total density of states (TDOS) and partial density of states (PDOS) of pristine CaSiO_3_ and Ta-substituted CaSi_1−*x*_Ta_*x*_O_3_ at *x* = 12.5% and 25%. The DOS analysis provides detailed insight into the orbital contributions, electronic hybridization, bonding character, band-gap nature, and dopant-induced states that control electrical conductivity and charge-transport behavior.^76,78^ The PDOS overlap is used here to identify the orbital character and possible mixing of the states; it is not treated by itself as a quantitative measure of bond strength, covalency, or charge transfer. For the Ta-substituted compositions, the DOS was resolved into the two spin channels, which are displayed on opposite sides of the zero-DOS axis in Fig. 5. This representation permits the spin asymmetry of the Ta-5d and O-2p contributions near the Fermi level to be compared directly as a function of Ta concentration.

For pristine CaSiO_3_, the DOS shows a clear electronic gap around the Fermi level, which is consistent with the indirect band gap obtained from the band-structure analysis. The valence-band region is mainly dominated by O-2p states, with minor contributions from Si-3p and Ca-derived states. In contrast, the conduction-band edge is primarily associated with Si-derived antibonding states, together with a small contribution from Ca states, which is typical of oxide perovskites. The absence of electronic states at the Fermi level confirms the insulating character of pristine CaSiO_3_ and indicates that the SiO_6_ octahedral framework remains electronically undistorted. The dominant O-2p contribution in the valence band and Si-derived contribution near the conduction-band edge suggest that the main electronic excitations arise from O-2p → Si-derived antibonding transitions. This behavior is consistent with reported DOS features of silicate and oxide perovskites, where the band-gap magnitude and band-edge positions are strongly influenced by structural symmetry, octahedral distortion, and electron-correlation effects.^53,80^

Substitution of Ta at the Si site in CaSiO_3_ produces a pronounced modification of the electronic structure, particularly near the band edges. The DOS profile shows the emergence of additional electronic states near the conduction-band minimum and close to the Fermi level, with dominant Ta-5d character and overlapping contributions from neighboring O-2p states. This spectral overlap is consistent with Ta-5d/O-2p orbital mixing, although the PDOS alone does not establish the magnitude of Ta–O bond covalency. These donor-derived states progressively extend into the Fermi-level region; therefore, the 12.5% Ta composition is interpreted as a degenerate donor-doped n-type system with incipient metallic character rather than as a conventional narrow-gap semiconductor, whereas the 25% Ta composition exhibits metallic behavior. With increasing Ta concentration, the contribution of Ta-5d states becomes more dominant, and their spectral weight at and across the Fermi level increases, consistent with progressively stronger carrier delocalization. This behavior is characteristic of transition-metal-doped oxide perovskites, where dopant-induced d–p orbital contributions near the conduction edge can generate donor-like states, modify charge-transfer pathways, and enhance the electronic response of the host perovskite lattice.^86^ Importantly, the spin-resolved DOS at 12.5% Ta exhibits a finite imbalance between the two spin channels within the Ta-derived near-*E*_F_ manifold, with accompanying O-2p weight. This identifies the Ta-5d-dominated donor states and the surrounding oxygen electronic environment as the principal electronic components associated with the finite spin polarization. At 25% Ta, the spin-resolved spectral weight becomes substantially more balanced between the two channels while the Ta-5d states broaden across *E*_F_. The reduced spin asymmetry is therefore consistent with the nearly quenched net magnetic moment at the higher Ta concentration.

The emergence of these states also provides the electronic-structure basis for the charge-compensation model introduced in Section 2. In the formal ionic picture, replacement of Si^4+^ by nominal Ta^5+^ corresponds to one additional donor electron per substituted Ta site. The present calculations contain no explicitly introduced oxygen vacancies, cation vacancies, interstitials, or other compensating point defects. Because the all-electron periodic cells are calculated as globally neutral systems, the nuclear and electronic charges are balanced self-consistently, and the nominal oxidation-state labels are not imposed as fixed atomic charges. Within this defect-free model, the donor-associated electronic charge therefore remains within the electronic system and is redistributed among the available states of the substituted lattice.

The increased Ta-5d spectral weight near the conduction-band edge and *E*_F_ is consistent with accommodation of this donor-associated charge in Ta-derived conduction states, with additional O-2p contributions reflecting the surrounding oxygen environment. This interpretation does not imply that the PDOS directly measures the amount of transferred charge; rather, it identifies the orbital character of the states involved. Quantitative determination of atomic charge transfer or oxidation state would require a separate charge-partitioning analysis, while determination of competing defect-compensation mechanisms would require explicit defect-formation-energy calculations. Accordingly, the present DOS/PDOS results support the electronically compensated, defect-free substitution limit but do not establish the equilibrium defect chemistry of experimentally synthesized Ta-substituted CaSiO_3_. Likewise, the spin-resolved PDOS is used here to identify the orbital character of the spin imbalance rather than to assign the magnetic moment to a single isolated atom; the corresponding site-resolved magnetic moments and real-space spin-density distributions are examined independently in Sections S1 and S2.

In the PDOS analysis, it is evident that:

➢ Ta-5d orbitals are the main electronic states that feature around the Fermi level, which implies that they have a significant impact on the shape of the near-*E*_F_ electronic behavior.

➢ The introduction of Ta causes broadening of the O-2p and Si-3p features, which is consistent with changes in orbital mixing and the local electronic environment accompanying Ta-induced lattice distortion.

➢ The influence of Ca-derived states is not very significant at the Fermi level implying that Ca plays a very small role in the electronic activity of the area around *E*_F_.

➢ The spin-resolved Ta-5d/O-2p states show the clearest finite spin imbalance at 12.5% Ta, whereas the two spin channels become much more closely compensated at 25% Ta.

This kind of spectral density redistribution is typical of oxides doped systems, in which the aliovalent dopant provides donor-derived states that modify the band-edge electronic structure and, at sufficiently high occupation, can evolve into extended states at the Fermi level.^81,82^

For the present structures, the near-EF Ta-5d contributions provide an important link between the aliovalent substitution and electronic compensation. At 12.5% Ta, the donor-associated states remain comparatively less delocalized but extend into the Fermi-level region, consistent with the onset of a degenerate donor-doped regime, whereas their increased spectral weight and dispersion at 25% indicate greater participation in extended metallic states. This concentration-dependent redistribution is consistent with the nominal increase in donor-electron population introduced by increasing Ta content. The accompanying evolution of the two spin channels indicates that increasing Ta content does not simply increase the spin polarization in proportion to the Ta-derived DOS. Instead, the higher Ta concentration produces a broader and more delocalized Ta-derived electronic manifold with substantially reduced net spin asymmetry, providing an electronic-structure basis for the concentration-dependent suppression of magnetism.

Upon Ta incorporation, the conduction-band DOS shows reduced dispersion and partial flattening near the Fermi level, suggesting an increase in carrier effective mass and partial localization of electrons around the TaO_6_ octahedral units. At 12.5% Ta concentration, Ta-derived donor states extend to the Fermi-level region; when interpreted together with the band crossing and small Fermi-surface pockets reported in Sections 3.4.1 and 3.4.3, this composition is therefore classified as a degenerate n-type system with incipient metallic character rather than as an ordinary narrow-gap semiconductor. When the Ta concentration is increased to 25%, the Ta-5d states become more intense and broaden significantly, eventually crossing the Fermi level. This indicates the formation of more delocalized conduction channels and establishes the evolution from the degenerate donor-doped regime at 12.5% Ta to a metallic state at 25% Ta. The resulting nonzero density of states at EF for the 25% composition is a defining electronic signature of metallic behavior. With increasing doping, Ta-5d orbitals dominate the dopant contribution to the conduction-band edge, and their spectral overlap with O-2p states also becomes more evident. This overlap is consistent with increased Ta-5d/O-2p orbital mixing but should not, by itself, be interpreted as quantitative proof of stronger Ta–O covalent bonding. The increasing continuity of the Ta-derived states, together with the O-2p contribution, is consistent with greater electronic delocalization and the development of extended conduction states. The evolution from isolated donor levels at lower Ta concentration to a more continuous Ta-5d-derived band at higher concentration indicates the emergence of itinerant carriers and conducting pathways. From the charge-compensation perspective, this evolution provides a physically consistent pathway for accommodation of the additional donor-associated electronic charge: at lower Ta content the additional electronic states remain closer to the conduction-band edge, whereas at higher Ta content the Ta-derived states broaden and intersect *E*_F_, allowing the added electronic density to become increasingly delocalized. No separate compensating defect is required within the particular stoichiometric supercell model used in these calculations. Although the electronic structure is strongly modified near the Fermi level, the Si-3p and O-2p states remain largely preserved, suggesting that the basic CaSiO_3_ lattice framework is not significantly disrupted. Instead, the electronic activity near the Fermi level is mainly dominated by Ta-derived states. Accordingly, the DOS results support the same concentration-dependent sequence established from the band structure: wide-gap semiconductor/intrinsic insulating limit (pristine) → degenerate n-type/incipient metallic regime (12.5% Ta) → metallic regime (25% Ta). This concentration-dependent evolution is consistent with dopant-induced electronic transitions commonly observed in oxide perovskites.^83,84^

Systematic evolution:

➢ The Ta-5d states move gradually to and finally reach the Fermi level.

➢ The Ta-5d/O-2p spectral overlap becomes more pronounced with Ta incorporation, consistent with increased orbital mixing; quantitative changes in bond strength require an independent bonding analysis.

➢ The electronic regime evolves from a wide-gap semiconductor/intrinsic insulating limit to a degenerate n-type state with incipient metallic character and finally to a metallic state.

➢ Lattice distortions are important in tuning the bandwidth and determine the localization of the electrons.

➢ The finite spin-channel asymmetry at 12.5% Ta is strongly reduced at 25% Ta, consistent with the concentration-dependent quenching of the net magnetic response.

The calculated DOS trends further support the concentration-dependent electronic evolution summarized in Table 3 and identify the near-Fermi-level states that underpin the optical and transport responses discussed in the subsequent sections. Within the present analysis, these DOS features are used to establish electronic-state reconstruction rather than to demonstrate application-level performance. A clear and systematic evolution of the electronic configuration is observed across the three compositions. Pristine CaSiO_3_ exhibits a wide band gap, with its electronic behavior mainly governed by the Si–O bonding network.

At 12.5% Ta concentration, Ta-5d donor-derived states extend into the Fermi-level region, producing a degenerate n-type electronic state with incipient metallic character. When the Ta concentration increases to 25%, these states broaden further and merge into a more continuous Ta-derived electronic band crossing the Fermi level, thereby establishing a metallic electronic state. Within the present substitution model, this concentration-dependent increase in Ta-derived states is also consistent with accommodation of the nominal additional electron introduced by each Ta-for-Si substitution. The calculated DOS therefore connects the formal donor picture with the observed increase in near-*E*_F_ electronic states, while avoiding an assumption that a particular intrinsic defect is responsible for compensation. This gradual evolution from an intrinsic wide-gap semiconductor to a degenerate donor-doped regime and finally to a metallic state reflects a d-electron-based tuning mechanism in oxide perovskites. The two Ta concentrations therefore represent distinct calculated electronic regimes rather than established device categories: the 12.5% Ta composition exhibits degenerate donor-doped/incipient-metallic behavior, whereas the 25% Ta composition develops a continuous metallic Ta-derived manifold at *E*_F_. Their relevance to electronic, thermoelectric, electrode, contact-layer, or other oxide-device functions would require application-specific transport parameters, interface-level assessment, and experimental validation beyond the DOS analysis presented here. As summarized in Table 4, Ta substitution produces a systematic evolution from a wide-band-gap insulating parent phase to a degenerate donor-doped n-type regime with incipient metallic character and finally to a metallic state at higher Ta concentration.

**Table 4:** Summary of electronic structure evolution in CaSiO_3_ with Ta substitution. The DOS-derived classification is made consistent with the band-structure and Fermi-surface results, showing evolution from the wide-gap parent phase to a degenerate donor-doped regime at 12.5% Ta and a metallic state at 25% Ta

Composition	Bandgap character	States near *E*_F_	Orbital hybridization	Electronic regime	Physical interpretation
CaSiO_3_ _(parent)	Wide bandgap	No DOS at *E*_F_; VB: O-2p, CB: Si-3p/3s	O-2p–Si-3p	Wide-gap semiconductor/intrinsic insulating limit	Robust SiO_6_ octahedral network
CaSi_1−*x*_Ta_*x*_O_3_ (*x* = 12.5%)	Reduced host-band separation with Fermi-level occupation	Ta-5d spectral weight extends to *E*_F_	Ta-5d–O-2p	Degenerate n-type/incipient metallic	Donor-induced Fermi-level occupation; onset of itinerant conduction
CaSi_1−*x*_Ta_*x*_O_3_ (*x* = 25%)	Gap closure	Ta-5d states cross *E*_F_	Strong Ta-5d–O-2p	Metallic	Continuous Ta-5d/O-2p-derived conduction states

#### 3.4.3. Fermi-surface topology and quantitative carrier-concentration analysis

The Fermi-surface topology and nominal carrier concentration of pristine and Ta-substituted CaSiO_3_ was investigated to understand the Ta-driven semiconductor-to-metal transition. However the pristine compound exhibits no Fermi surface as no band intersects the Fermi level, consistent with its broad band gap semiconducting properties. Near the Fermi level, Ta substitution is responsible for the appearance of Ta-5d/O-2p hybridized states, resulting in small Fermi pockets at 12.5% Ta and larger, more connected Fermi-surface sheets at 25% Ta. The finite Fermi pockets at 12.5% Ta establish a degenerate n-type, incipient metallic regime rather than a conventional non-degenerate semiconductor, whereas the connected Fermi-surface sheets at 25% Ta establish a metallic state. This progressive evolution indicates greater carrier delocalization, and the concentration-driven progression from the wide-gap semiconducting parent to a degenerate donor-doped regime and finally to a metallic state.

It is important to distinguish the directly calculated Fermi-surface topology from the nominal carrier-density estimate discussed below. The Fermi surfaces are obtained from the self-consistent electronic bands intersecting *E*_F_, whereas the carrier-density estimate assumes one nominal donor electron for each Ta-for-Si substitution. Thus, the Fermi-surface calculation provides independent reciprocal-space evidence that the additional electronic states associated with Ta substitution are accommodated within the electronic system rather than being removed through an explicitly imposed compensating defect.

##### 3.4.3.1 Fermi-surface analysis

**Fig. 6:**
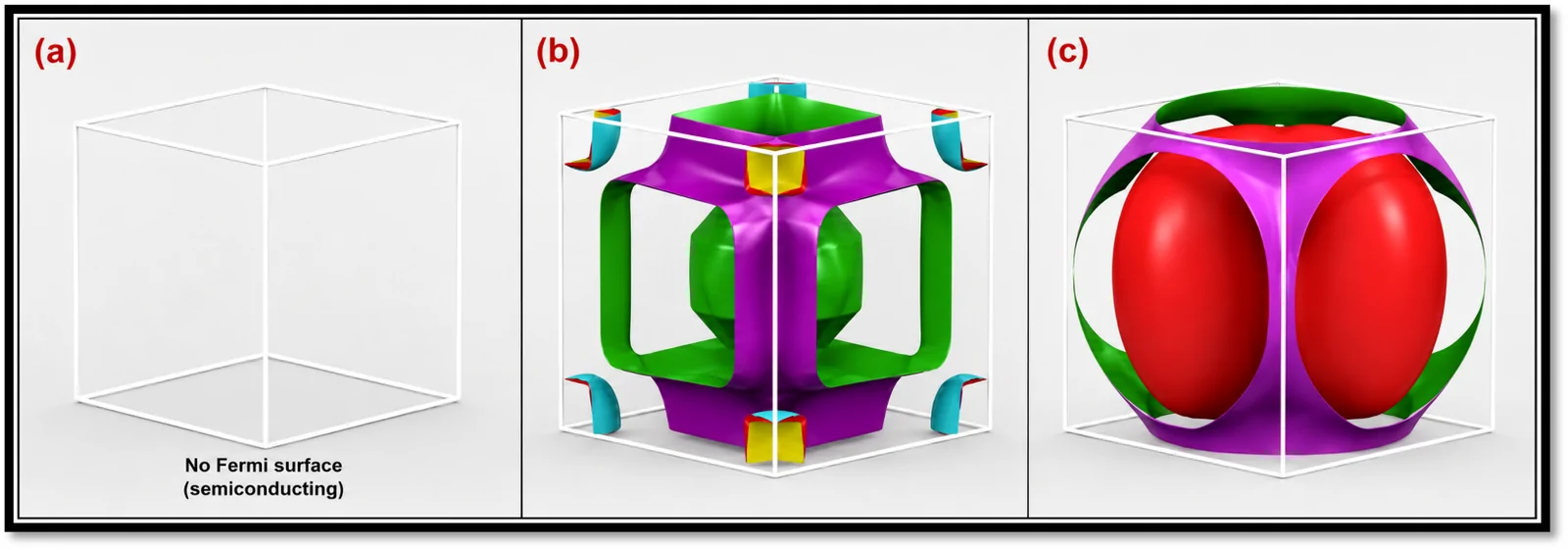
Comparative Fermi-surface visualization of (a) pristine CaSiO_3_, (b) 12.5% Ta-doped CaSi_1−*x*_Ta_*x*_O_3_, and (c) 25% Ta-doped CaSi_1−*x*_Ta_*x*_O_3_. The parent compound shows no Fermi surface because no band crosses *E*_F_, while Ta substitution generates small Fermi pockets at 12.5% and larger connected Fermi sheets at 25%, identifying the 12.5% composition as a degenerate n-type/incipient metallic regime and the 25% composition as a metallic state. Within the defect-free substitution model, the emergence and growth of these Fermi surfaces are consistent with electronic accommodation and increasing delocalization of the donor-associated charge introduced by Ta substitution.

The reciprocal-space evidence for the semiconductor-to-metal transition induced by Ta in CaSiO_3_ was obtained by performing Fermi-surface calculations. The electronic band structure has been computed by the FP-LAPW method as implemented in the program WIEN2k^87^ and the resulting Fermi surface sheets were displayed from the bands that cross the Fermi level by XCrySDen and FermiSurfer.^88,89^ The Fermi surface exists only when electronic states are present at (*E*_F_), and pristine CaSiO_3_ is a wide-band-gap semiconducting material [Fig. 6(a)], also in line with its electronic structure. The small multi-sheet Fermi pockets near *E*_F_ observed after 12.5% Ta substitution at the Si site [Fig. 6(b)] demonstrate finite occupation at the Fermi level and therefore identify this composition as a degenerate n-type donor-doped system with incipient metallic character, rather than as a conventional narrow-gap semiconductor. The DOS and band-structure results indicate that these pockets are primarily created by Ta-5d/O-2p hybridized states very close to the conduction-band edge. Within the defect-free substitution model used here, these Fermi pockets provide reciprocal-space evidence for electronic accommodation of the nominal donor charge introduced by Ta-for-Si substitution. The calculations do not contain an explicitly introduced vacancy, interstitial, or other compensating point defect; instead, the additional electronic population associated with the formal Ta^5+^/Si^4+^ valence difference contributes to the self-consistently obtained near-*E*_F_ states. The Ta-5d/O-2p description refers to the orbital character of these states inferred from the PDOS and should not, by itself, be interpreted as a quantitative measure of Ta–O bond strength. Increasing the Ta concentration to 25% [Fig. 6(c)], the Fermi-surface topology grows in size and connectivity, in line with the increased band crossing at *E*_F_ and thus the emergence of delocalized n-type transport channels. At this concentration, the connected Fermi-surface sheets and stronger band crossings provide direct reciprocal-space evidence of a metallic electronic state. The evolution of the Fermi surface from no Fermi surface to isolated pockets and finally to connected sheets is a clear indication of the doping-driven semiconductor to metal transition.^88,89^

More specifically, the sequence is consistently classified here as wide-gap semiconductor/intrinsic insulating limit (pristine) → degenerate n-type/incipient metallic regime (12.5% Ta) → metallic n-type regime (25% Ta). The increase in Fermi-surface volume and connectivity with Ta content is consistent with a larger population of electronically active donor-associated states. In the present stoichiometric supercells, this behavior is therefore interpreted as electronic compensation of the aliovalent substitution. This interpretation applies specifically to the defect-free computational limit investigated here; experimentally, competing acceptor-type intrinsic defects may modify the free-carrier population depending on chemical potentials and synthesis conditions. Their relative stability cannot be established without explicit defect-formation-energy calculations and is not assumed in the present model. The main Fermi-surface features and the corresponding electronic transition are summarized in Table 5.

**Table 5:** Fermi-surface interpretation and electronic-transition summary for pristine and Ta-doped CaSiO_3_ systems

Materials	Band/Fermi-level behavior	Fermi-surface observation	Carrier character	Physical implication
CaSiO_3__(parent)	Wide indirect band gap; no electronic band crosses *E*_F_	No Fermi surface	Negligible intrinsic carriers	Wide-gap semiconducting reference; intrinsic insulating limit
CaSi_1−*x*_Ta_*x*_O_3_ (*x* = 12.5%)	Ta-5d/O-2p donor-derived states intersect *E*_F_	Small multi-sheet Fermi pockets	Degenerate n-type donor carriers	Degenerate n-type/incipient metallic regime
CaSi_1−*x*_Ta_*x*_O_3_ (*x* = 25%)	Ta-derived bands broaden and cross *E*_F_ more strongly	Large and connected Fermi-surface sheets	n-type metallic carriers	Metallic state with delocalized transport

The carrier character listed in Table 5 refers to the defect-free electronically compensated substitution model. No explicit compensating point defects were included in the Fermi-surface calculations.

Overall, the Fermi-surface topology independently corroborates the concentration-dependent electronic classification established from the band structure and DOS: pristine CaSiO_3_ is a wide-gap semiconductor in the intrinsic insulating limit, 12.5% Ta is a degenerate n-type system with incipient metallic character, and 25% Ta is metallic with delocalized n-type carriers.

##### 3.4.3.2 Quantitative carrier-concentration analysis

The nominal donor-electron density was estimated from the aliovalent Ta introduced at the Si site. This model assumes that each nominal Ta^5+^ substitution for Si^4+^ contributes one additional donor electron in formal electron counting, and the electron density due to Ta substitution can be estimated from the number of substituted Ta atoms and the relaxed supercell volume. Accordingly, the values obtained here represent nominal stoichiometric donor-density estimates under the assumptions of one donor electron per Ta, complete electronic activation, and the absence of explicit compensating defects. They should not be interpreted as independently calculated equilibrium free-carrier concentrations. This carrier-density estimate is complementary to the BoltzTraP/BoltzTraP2 transport analysis, which is designed to assess the transport response at different temperatures and chemical potentials.^90,91^ The nominal carrier concentration was estimated from:4
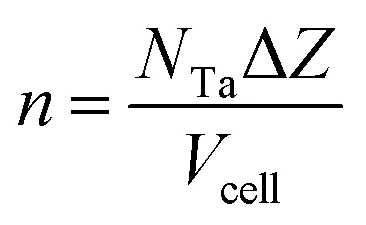


Since the relaxed supercell volumes are expressed in Bohr^3^, eqn (4) was rewritten in (cm^−3^) by applying the Bohr^3^-to-cm^3^ volume conversion, as given in eqn (5). The converted expression was then used to calculate the nominal carrier concentrations reported in Table 6.5
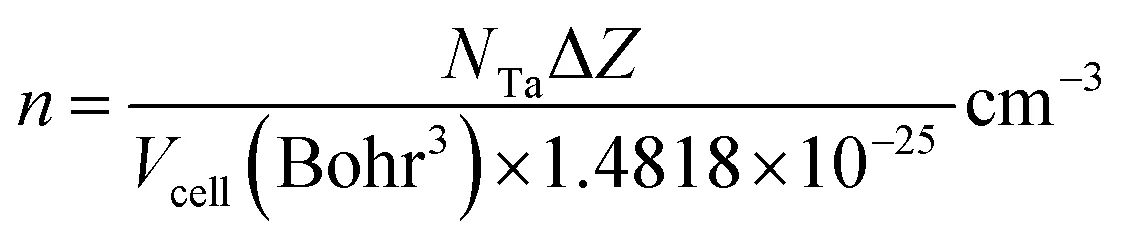
In this case, (*n*) denotes the nominal donor-electron density, (*N*_Ta_) is the number of Ta atoms in the relaxed supercell, (Δ*Z* = 1) represents the formal one-electron donor difference associated with nominal Ta^5+^ substitution for Si^4+^, and (*V*_cell_) is the relaxed supercell volume. The conversion factor (1 Bohr^3^ = 1.4818 × 10^−25^ cm^3^) was applied because the optimized volumes are reported in Bohr^3^. This formulation follows the standard carrier-density definition as the number of nominal donor electrons per unit volume in the present stoichiometric model, where the donor contribution is assigned from aliovalent Ta substitution at the Si site.^92,93^ The formal oxidation-state notation is used only for electron counting and does not imply that fixed +5 and +4 atomic charges were imposed in the self-consistent DFT calculations.

**Table 6:** Nominal donor-electron density estimated from Ta content and relaxed supercell volume for pristine and Ta-substituted CaSiO_3_ systems

Materials	Ta content	Relaxed volume *V* (Bohr^3^)	*N*_Ta_	Δ*Z*	Nominal donor density, *n* (cm^−3^)	Transport regime
CaSiO_3__(parent)	0%	2533.4554	0	—	≈0 (negligible)	Wide-gap semiconductor; intrinsic insulating limit
CaSi_1−*x*_Ta_*x*_O_3_ (*x* = 12.5%)	12.5%	2755.1025	1	1	2.45 × 10^21^	Degenerate n-type/incipient metallic regime
CaSi_1−*x*_Ta_*x*_O_3_ (*x* = 25%)	25%	3155.7362	2	1	4.28 × 10^21^	Metallic n-type regime

**Fig. 7:**
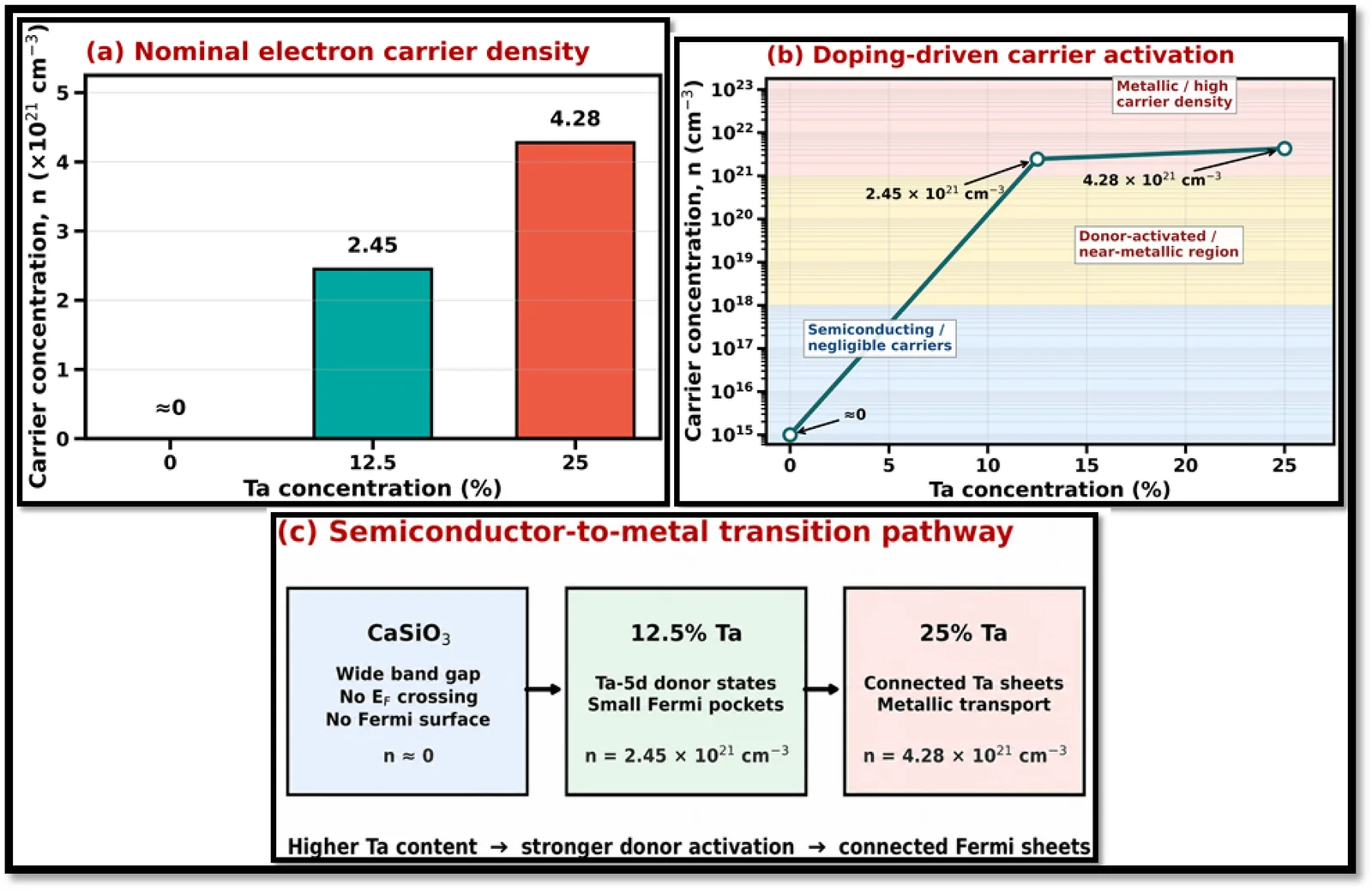
Nominal donor-density analysis of pristine and Ta-substituted CaSiO_3_ systems: (a) nominal donor-electron density, (b) logarithmic variation of nominal donor density with Ta content, and (c) schematic comparison with the corresponding electronic-structure evolution. The values of 2.45 × 10^21^ and 4.28 × 10^21^ cm^−3^ for 12.5% and 25% Ta, respectively, are stoichiometric donor-density estimates based on formal one-electron donor counting and are not independently calculated equilibrium free-carrier concentrations. Their increasing trend is consistent with the development of Ta-derived states near *E*_F_ and the calculated Fermi-surface evolution. In conjunction with the band, DOS, and Fermi-surface results, this evolution is consistent with a degenerate n-type/incipient metallic state at 12.5% Ta and a metallic state at 25% Ta.

Using the optimized supercell volumes, the nominal donor-electron density increases from approximately zero for pristine CaSiO_3_ to (2.45 × 10^21^ cm^−3^) for 12.5% Ta and (4.28 × 10^21^ cm^−3^) for 25% Ta (Table 6 and Fig. 7(a)). These values represent nominal stoichiometric donor-density estimates based on one donor electron per substituted Ta atom and the absence of explicit compensating defects; they should not be interpreted as independently calculated equilibrium free-carrier concentrations. The actual equilibrium free-carrier concentration could be lower if compensating intrinsic defects, incomplete ionization, carrier localization, or other many-body effects occur experimentally. Accordingly, the numerical donor-density estimates are not used by themselves to define the electronic regime; the classification is based primarily on the calculated band crossings, finite DOS at *E*_F_, and Fermi-surface topology. The logarithmic plot in Fig. 7(b) highlights the increase in nominal donor density from the pristine composition toward the higher-Ta substitution levels. The schematic in Fig. 7(c) compares this nominal increase with the independently calculated Fermi-surface evolution. The progressive appearance and connectivity of Fermi-surface sheets provide electronic-structure evidence that Ta-derived states become increasingly occupied and delocalized with substitution, whereas the numerical values of *n* themselves arise from stoichiometric donor counting rather than direct integration of the Fermi-surface volume. Therefore, agreement between the increasing nominal donor density and the band-structure, DOS, and Fermi-surface trends supports a Ta-induced increase in electronically active states near *E*_F_, while avoiding the stronger claim that every nominal donor electron necessarily contributes one mobile free carrier under experimental conditions. Taken together with the electronic-structure results, these trends support the concentration-dependent sequence from a wide-band-gap semiconductor in pristine CaSiO_3_ to a degenerate n-type/incipient metallic regime at 12.5% Ta and a metallic regime at 25% Ta.^91–93^

#### 3.4.4. Real-space charge density and orbital-resolved bonding topology

**Fig. 8:**
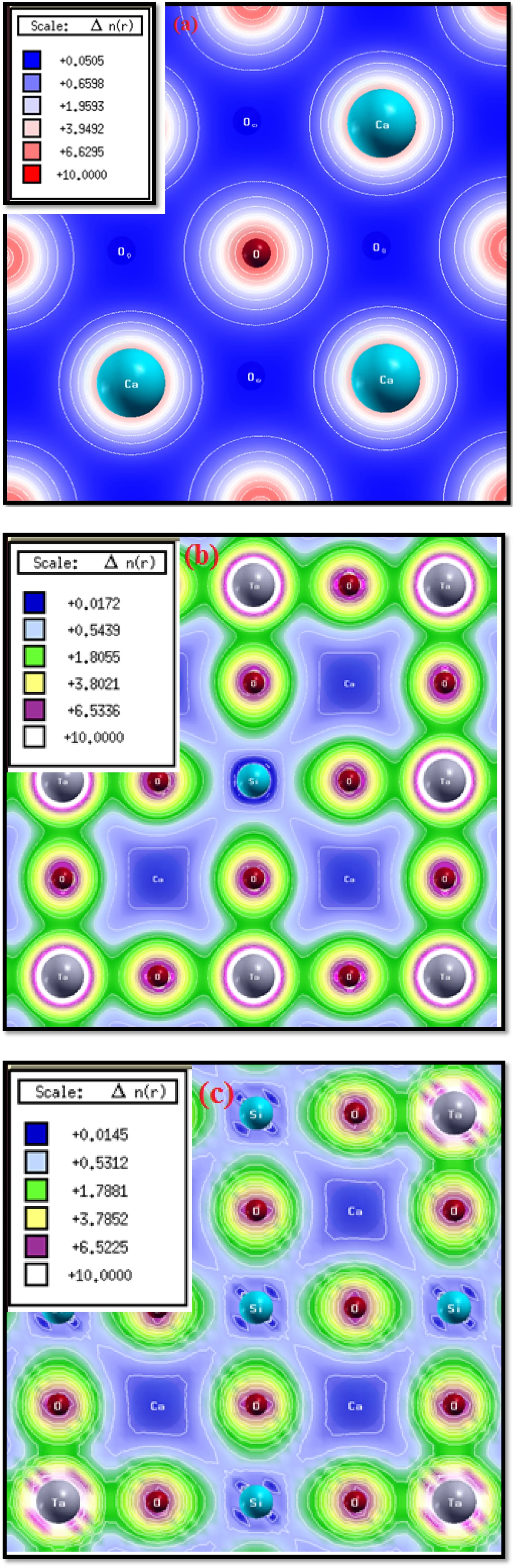
Real-space charge-density maps of (a) pristine CaSiO_3_, (b) CaSi_1−*x*_Ta_*x*_O_3_ (*x* = 12.5%), and (c) CaSi_1−*x*_Ta_*x*_O_3_ (*x* = 25%). Ta substitution produces progressive charge redistribution and increasing spatial delocalization around the Ta/O environments. These real-space trends are consistent with, but do not independently establish, the degenerate n-type/incipient metallic state at 12.5% Ta and the metallic state at 25% Ta identified from the band-structure, DOS, and Fermi-surface analyses.

The electron-density distributions of CaSiO_3_ and Ta-substituted CaSi_1−*x*_Ta_*x*_O_3_ at *x* = 12.5% and 25%, shown in Fig. 8(a–c), illustrate the evolution of atomic bonding and charge redistribution with increasing Ta substitution. In pristine CaSiO_3_, the electron density is mainly localized around oxygen atoms, with directional charge accumulation along the Si–O bonds, which is consistent with a substantial covalent contribution to the Si–O framework, whereas the Ca–O interaction shows a more ionic character. This charge distribution reflects a mixed bonding nature, in which the covalent Si–O framework dominates the electronic structure while Ca contributes mainly through ionic interaction. Such bonding is consistent with the wide-band-gap semiconducting behavior of pristine CaSiO_3_.^85^ This interpretation is also supported by the ionicity values of Ca–O (∼77%) and Si–O (∼45%), suggesting dominant ionic and covalent contributions, respectively. In interpreting the charge-density redistribution after Ta substitution, the aliovalent nature of the substitution must be considered explicitly. In the formal ionic description, replacement of nominal Si^4+^ by nominal Ta^5+^ corresponds to a donor-type substitution and introduces one nominal donor electron per substituted Ta site. However, these formal oxidation states are not imposed as fixed atomic charges in the DFT calculation. The periodic supercells are treated as overall charge-neutral systems, and the electronic density is obtained self-consistently from the atomic composition and electronic states of each structure. In the Ta-substituted systems, the charge-density maps show stronger charge redistribution around the TaO_6_ octahedra, which is consistent with the Ta-5d/O-2p orbital mixing identified independently by the PDOS analysis rather than serving, by itself, as a quantitative measure of hybridization strength. This redistribution promotes charge delocalization, weakens the purely localized bonding picture, and supports the formation of donor-derived electronic states at and around the Fermi level. With increasing Ta content from 12.5% to 25%, the charge redistribution becomes more pronounced, indicating a progressively more spatially extended donor-associated electronic distribution around the Ta/O environment. Such behavior is consistent with Ta-doped oxide perovskites, where Ta substitution introduces donor-derived states and promotes n-type carrier transport.^53^ No oxygen vacancy, cation vacancy, interstitial, or other explicit charge-compensating point defect was introduced in the present Ta-substituted structures. Consequently, the model considered here represents the defect-free electronic-compensation limit of aliovalent Ta substitution. In this limit, the donor-associated electronic charge remains within the calculated electronic system and is redistributed among the available Ta- and O-associated states rather than being compensated by an explicitly constructed defect. The real-space charge-density maps therefore provide complementary evidence for the redistribution of this additional electronic density around the substituted region. Importantly, these maps are used here as complementary real-space evidence and not as an independent criterion for assigning metallicity; the electronic classification is established primarily from the band crossings, DOS at *E*_F_, and Fermi-surface topology.

When 12.5% Ta is introduced, localized charge accumulation is observed around the Ta sites, which is consistent with partially occupied Ta-5d/O-2p-derived states identified by the electronic-structure calculations. This interaction perturbs the surrounding SiO_6_ framework and introduces partially occupied donor-derived states at the band edge and *E*_F_, consistent with the degenerate n-type/incipient metallic regime established in Sections 3.4.1–3.4.3. At this concentration, the Ta–O bonds exhibit intermediate ionicity (∼55–58%), indicating a mixed ionic–covalent bonding character that is distinct from the Si–O bonding environment of the pristine host. At this lower substitution level, the localized redistribution around the Ta-centered environment is consistent with accommodation of the nominal donor electron in states located close to the conduction-band edge and *E*_F_, as independently indicated by the band-structure and DOS/PDOS analyses. Thus, global charge neutrality is maintained without removing the donor-associated electron or introducing an artificial compensating background; the neutral supercell retains the full self-consistent electronic population defined by its atomic composition.

At the higher Ta concentration of 25%, the charge associated with Ta–O bonds becomes less localized and extends through longer d–p conduction pathways within the lattice. This enhanced delocalization is consistent with the emergence of metallic behavior, as also supported by the finite density of states at the Fermi level. The Ta–O bonds retain an intermediate mixed ionic–covalent character (∼55–58% ionicity), but the increased spatial continuity of the electronic density is consistent with stronger electronic delocalization and broader Ta/O-derived participation near *E*_F_; it should not be interpreted by itself as quantitative proof of an increase in covalent bond strength. Similar behavior has been reported for Ta-substituted oxide perovskites, where increasing Ta content promotes conduction-band broadening and carrier delocalization. These trends are consistent with the metallic state obtained here at 25% Ta and with the development of extended Ta/O-derived electronic pathways identified by the band-structure, DOS, and Fermi-surface analyses.^86^

The greater spatial extension of charge at 25% Ta is therefore consistent with increased delocalization of the donor-associated electronic population as the concentration of Ta-derived states near *E*_F_ increases. This interpretation is also consistent with the larger and more connected Fermi-surface features obtained at the higher Ta concentration. Accordingly, the band structure, DOS/PDOS, Fermi-surface topology, and real-space charge density provide mutually consistent descriptions of electronic compensation within the particular defect-free supercell model adopted in this work. The empirical ionicity method suggested by Pauling may be applied to further estimate the ionic-covalent balance of the predominant bonds with the aim of quantifying it (eqn (6)).

6 *I*_b_ = 1 − exp[−0.25 (Δ*X*)^2^]

Ionicity approach of Pauling was also used to analyze the nature of bonding through the difference in electronegativity of Ca–O, Si–O and Ta–O pairs. The values obtained (about 77% Ca–O, about 45% Si–O and about 55–58% Ta–O) imply that Ca–O bonds are predominantly ionic and Si–O bonds are predominantly covalent whereas Ta–O bonds are of intermediate nature with both ionic and covalent bonding. These Pauling ionicity percentages are empirical descriptors based on electronegativity differences and are not equivalent to atomic charges obtained from a charge-partitioning analysis. Therefore, the nominal Ta^5+^ notation used in discussing aliovalent substitution should be regarded as a formal oxidation-state description rather than a directly calculated +5 charge on the Ta atom. Likewise, the charge-density and PDOS results provide qualitative evidence of charge redistribution and orbital participation but do not, by themselves, constitute a quantitative determination of bond order or charge transfer. The charge-density maps further support this behavior. In pristine CaSiO_3_, the electron density is mainly localized around oxygen atoms, while Ca shows a more ionic charge distribution, which is consistent with dominant Ca–O ionic interaction and a substantial covalent contribution within the Si–O framework. At 12.5% Ta substitution, the charge density between Ta and neighboring oxygen atoms becomes more pronounced, indicating Ta–O bond formation and partial charge redistribution. At the higher Ta concentration of 25%, the charge distribution becomes more continuous and extends between adjacent Ta–O units, consistent with stronger electronic delocalization and more extended Ta/O-derived orbital participation. This extended charge distribution is consistent with the electronic-structure results, where Ta-derived states become partially occupied at *E*_F_ at 12.5% Ta and cross *E*_F_ more strongly at 25% Ta, yielding the degenerate n-type/incipient metallic and metallic regimes, respectively. The shift from localized to extended charge distribution with increasing Ta content indicates that Ta substitution effectively tunes the electronic behavior of CaSiO_3_.

The present results therefore establish the physical meaning and limitation of the substitution model. For the stoichiometric Ta-substituted supercells investigated here, aliovalent substitution is treated through self-consistent electronic compensation, with the donor-associated charge accommodated in the evolving near-*E*_F_ electronic states. This does not imply that intrinsic defects are absent in experimentally synthesized CaSi_1−*x*_Ta_*x*_O_3_. Depending on oxygen chemical potential, synthesis conditions, and Fermi-level position, native acceptor-type defects or other compensating mechanisms may alter the equilibrium carrier population. Determining which defect is thermodynamically preferred would require explicit charged-defect formation energies as functions of chemical potential and Fermi level, which were not calculated in the present work. The current results should therefore be interpreted specifically as the intrinsic electronic response of the defect-free Ta-for-Si substitution model.

#### 3.4.5. COHP and ELF analysis of Ta–O bonding and electron localization

Projected density of states is used to determine the nature of the orbital makeup of the electronic structure, but the overlap of two components is not a bond metric by itself. To establish a microscopic connection between the Ta-induced structural changes and the resulting mechanical response, the electronic reconstruction was therefore examined using two complementary bonding descriptors: crystal orbital Hamilton population (COHP) in energy space and electron localization function (ELF) in real space. In COHP, bonding and antibonding contributions are separated for a given pair of atoms as a function of energy, whereas ELF describes the degree and spatial distribution of electron localization in real space and is not an energy-resolved bond-strength measure. The descriptors may be used in conjunction to tie in the Ta-5d/O-2p states identified by PDOS to the local Ta–O electronic environment rather than as a necessity for spectral coincidence to be an indicator of covalency.

The combined analysis therefore provides an independent bonding-level description that complements the structural information in Section 3.1 and the elastic response in Section 3.7. In this framework, the structural analysis identifies the Ta-induced modification of the local B–O coordination environment, COHP and ELF characterize the associated electronic reorganization, and the elastic calculations quantify the corresponding mechanical response. Neither PDOS overlap nor the mechanical parameters are used independently as measures of Ta–O bond strength.

##### 3.4.5.1 COHP analysis of Si–O and Ta–O bonding interactions

**Fig. 9:**
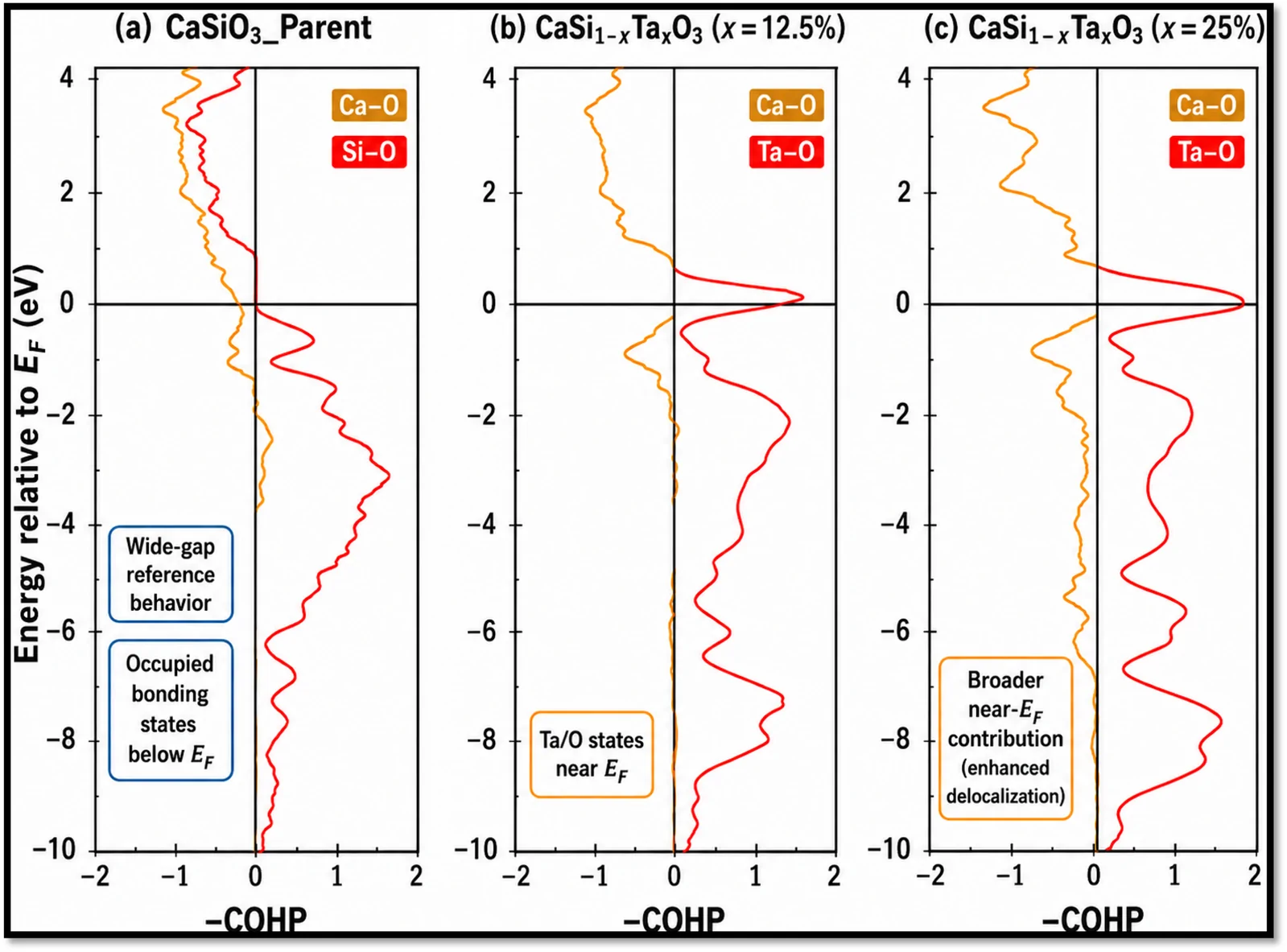
Energy-resolved –COHP profiles of (a) pristine CaSiO_3_, (b) 12.5% Ta-substituted CaSi_1−*x*_Ta_*x*_O_3_, and (c) 25% Ta-substituted CaSi_1−*x*_Ta_*x*_O_3_. *E*_F_ is set to 0 eV; positive and negative -COHP values denote bonding and antibonding contributions, respectively. The Ta–O response emerging near *E*_F_ provides bond-resolved evidence for the Ta/O interaction associated with the Ta-derived states identified by PDOS. The concentration-dependent broadening of the Ta–O response is interpreted as an evolution of the bonding manifold and not, in the absence of ICOHP, as quantitative evidence for monotonically increasing individual Ta–O bond strength.

The result is shown in Fig. 9(a), which serves as a reference for the bond-resolved pristine CaSiO_3_. Fermi energy is the reference energy and following the convention of -COHP, positive values indicate bonding contributions and negative values antibonding contributions. While the Si–O contribution is mostly bonding, the Ca–O contribution is weaker and more mixed in character, as it occurs below *E*_F_ on the occupied Si–O manifold. The lack of significant weight in the near-*E*_F_ region is indicative of the wide-gap electronic structure of the parent phase and can be used as a chemically resolved reference for the undoped SiO_6_ framework. This interpretation is consistent with the convention used for periodic solids to separate stabilizing and destabilizing contributions using COHP.^94^

The spectrum undergoes significant changes when 12.5% Ta is substituted [Fig. 9(b)]. There is a small Ta–O contribution throughout the occupied valence region and into the immediate vicinity of *E*_F_, whereas the Ca–O contribution is comparatively weak. This is the same energy window where the PDOS is dominated by Ta-5d character and contains neighboring O-2p weight. The two analyses provide different information: PDOS identifies the orbital contributions to the near-*E*_F_ states, whereas COHP resolves whether the corresponding pairwise interactions contain bonding or antibonding contributions as a function of energy. The complementary use of orbital- and bond-resolved descriptions is well established in COHP analyses of complex solids and perovskite-related systems.^94,95^ The coexistence of Ta/O spectral weight in the PDOS and a finite Ta–O COHP response within the same near-*E*_F_ interval provides bond-resolved support for an interacting Ta–O manifold; the Ta-5d/O-2p hybridization assignment is therefore no longer inferred from coincident PDOS peaks alone.

As the Ta content is raised to 25%, the Ta–O response widens and remains finite near *E*_F_ [Fig. 9(c)]. This broader energy distribution is consistent with the greater participation of Ta-derived states near and across *E*_F_, the Fermi-level crossings, and the more spatially connected electronic states identified from the band-structure and Fermi-surface analyses. The COHP result is thus understood to represent the evolution of the Ta–O bonding manifold with increasing concentration of the dopant. Because integrated COHP values (ICOHP) are not available here, the energy-resolved spectra are not used to quantitatively rank individual Ta–O bond strengths or to claim that each Ta–O bond becomes progressively more covalent with increasing Ta content. In recent work on perovskites, energy-resolved COHP features have likewise been distinguished from integrated metrics when discussing changes in bond strength.^96^

Instead, the COHP results provide the bond-resolved component of the structure–bonding–property relationship: the local B–O coordination changes established in Section 3.1 are accompanied by a modified Ta–O interaction manifold, while the resulting macroscopic response is independently quantified through the elastic constants and derived mechanical parameters in Section 3.7. This correlation does not require the mechanical data themselves to be interpreted as evidence of stronger Ta–O covalency.

##### 3.4.5.2 ELF analysis of electron localization and Ta–O charge redistribution

The energy-resolved COHP analysis is complemented by ELF, which characterizes electron localization in real space on a dimensionless scale from 0 to 1. Values approaching unity indicate strongly localized electronic regions, intermediate values indicate more delocalized or shared-electron environments, and low values correspond to weak localization. ELF is therefore useful for determining how Ta substitution redistributes the electronic density around the local B–O coordination environment and whether this redistribution remains localized or becomes spatially more continuous.^97^

**Fig. 10:**
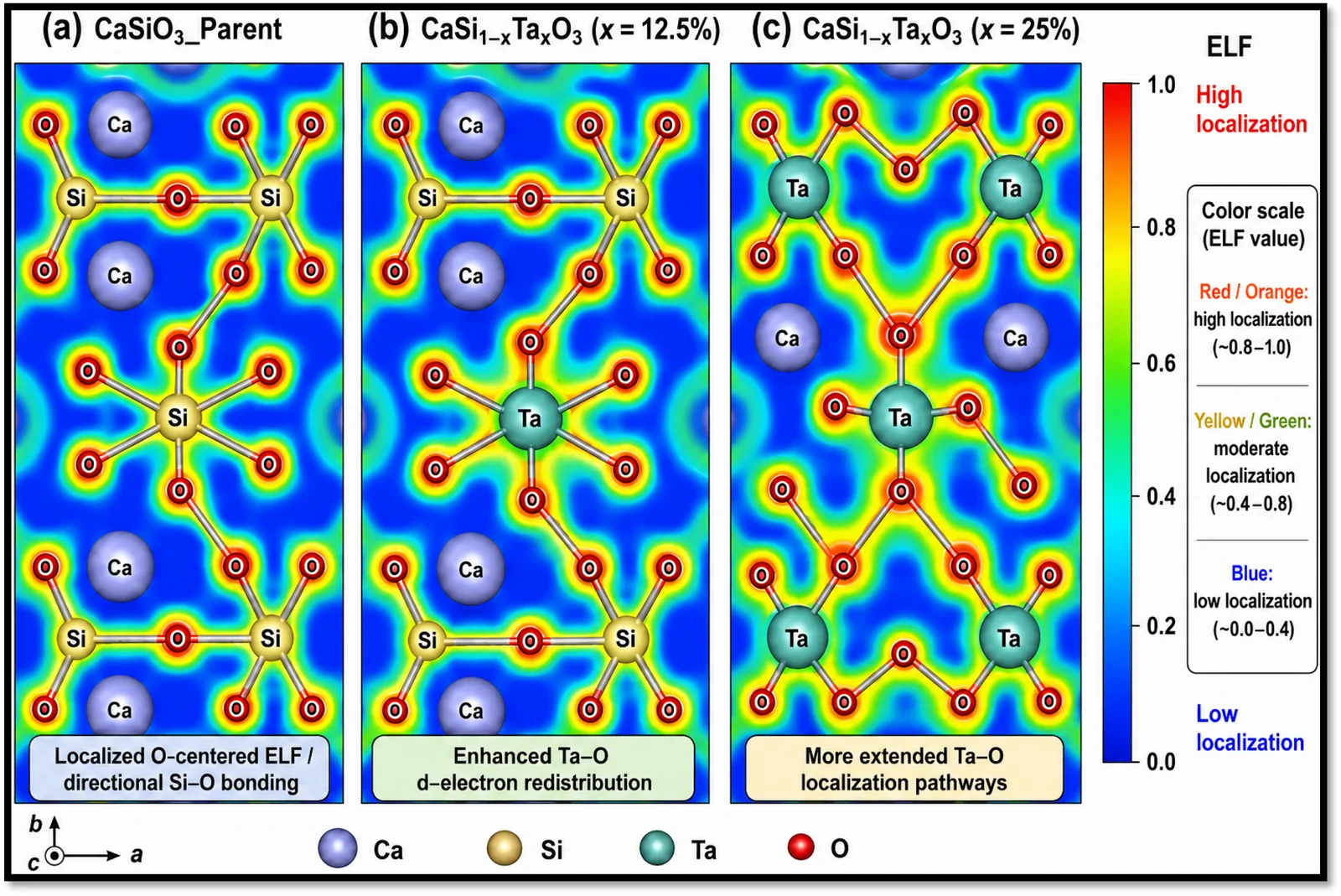
Electron localization function (ELF) maps of (a) pristine CaSiO_3_, (b) 12.5% Ta-substituted CaSi_1−*x*_Ta_*x*_O_3_, and (c) 25% Ta-substituted CaSi_1−*x*_Ta_*x*_O_3_. The 0–1 scale denotes low-to-high electron localization. Ta substitution produces a redistribution of the O-centered localization pattern around the Ta–O coordination environment, whereas the 25% composition exhibits greater spatial continuity of intermediate-ELF regions along Ta/O-containing pathways. This increased spatial continuity is interpreted as electronic redistribution and delocalization rather than as direct evidence for stronger individual Ta–O bonds.

The ELF map in pristine CaSiO_3_ shown in Fig. 10(a), exhibits the highest ELF values around O, with the Si–O regions showing the next most significant localization and the remaining regions exhibiting comparatively lower ELF. This distribution is consistent with a predominantly ionic Ca–O environment and a more directional shared-electron contribution within the Si–O subnetwork, and it provides the parent-state reference for assessing Ta-induced redistribution.^97^

For 12.5% Ta substitution [Fig. 10(b)], the O-centered high-localization regions remain, but the electronic topology around the TaO_6_ environment is redistributed relative to the parent SiO_6_ environment. Intermediate-ELF regions appear between Ta and neighboring O atoms, demonstrating a local redistribution of electronic density within the Ta-centered coordination environment. This local but still spatial ordering is consistent with the incipient-metallic regime where donor states originating from Ta are present in the Fermi-level region while retaining a recognizable local coordination environment. Examples of real-space localization maps for identifying ionic, shared-electron, and dopant-associated regions in substituted perovskites have also been reported.^98^

The ELF map thus supplies real-space evidence for Ta–O electronic redistribution that is independent of PDOS peak positions and complementary to the bond-selective COHP response.

At 25% Ta substitution [Fig. 10(c)], the intermediate-ELF regions become more spatially continuous through the Ta–O-containing network, while the highest localization remains concentrated mainly around O. The important change is therefore not a uniform increase in localization at each Ta–O contact, but the development of more extended Ta/O-derived electronic pathways across the lattice. This interpretation is consistent with the finite DOS at *E*_F_ and the larger, more connected Fermi-surface sheets of the metallic composition, while avoiding the incorrect equivalence of electronic delocalization with universally stronger local covalency. Comparable ELF-based bonding studies use the same real-space logic to distinguish local polarization from extended electronic connectivity.^99^

Taken together, the structural, PDOS, COHP, ELF, and mechanical results establish a sequential structure–bonding–property picture. Ta-for-Si substitution first modifies the local B–O coordination environment and produces concentration-dependent structural relaxation (Section 3.1); COHP then demonstrates a finite and composition-dependent Ta–O interaction manifold, while ELF reveals the accompanying real-space redistribution from a more localized Ta-centered environment at 12.5% Ta toward more spatially continuous Ta/O-derived pathways at 25% Ta. The elastic tensor and derived mechanical quantities in Section 3.7 independently quantify the associated changes in small-strain stiffness, anisotropy, and hardness. The resulting relationship therefore rests on complementary structural, bonding, and mechanical descriptors rather than on an assumption that increasing elastic constants directly demonstrate progressively stronger Ta–O covalency.

### 3.5. Frequency-dependent optical response and spectral optical properties

The optical response of pristine CaSiO_3_ and Ta-doped CaSi_1−*x*_Ta_*x*_O_3_ at *x* = 0.125 and 0.25 was investigated to understand how Ta incorporation modifies the interaction of these materials with electromagnetic radiation. Key optical processes, including absorption, reflection, refraction, transmission, and electronic excitation, were analyzed to obtain a complete picture of light–matter interaction in the modified electronic environment. To quantify these effects, the complex dielectric function *ε*(*ω*) = *ε*_1_(*ω*) + *iε*_2_(*ω*) was evaluated as a function of photon energy. The real part, *ε*_1_(*ω*), describes the polarization response and dispersion behavior of the material, whereas the imaginary part, *ε*_2_(*ω*), is directly related to interband electronic transitions and optical absorption. The electronic labels used in this optical discussion follow Section 3.4: pristine CaSiO_3_ is a wide-band-gap semiconductor in the intrinsic insulating limit, 12.5% Ta is a degenerate n-type system with incipient metallic character, and 25% Ta is metallic.

**Fig. 11:**
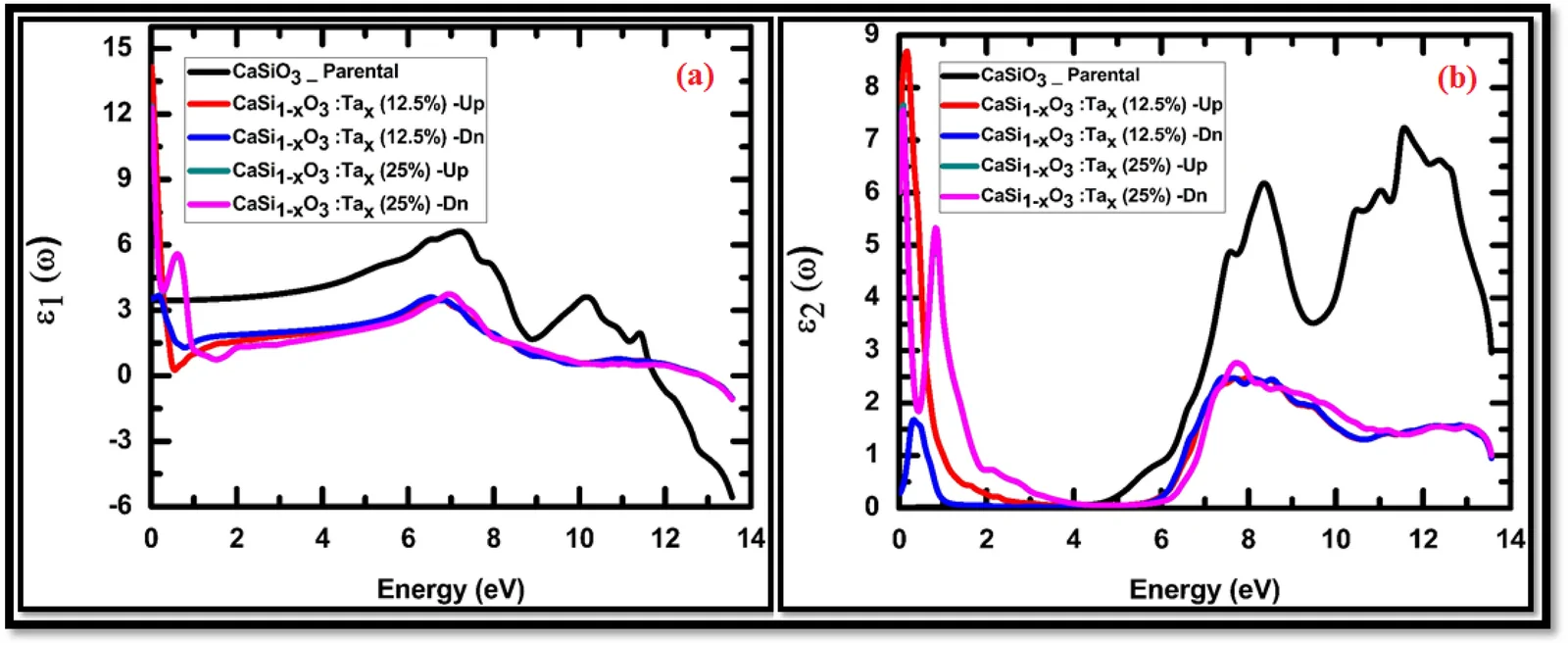
Real *ε*_1_(*ω*) (a) and imaginary *ε*_2_(*ω*) (b) dielectric functions of pristine CaSiO_3_ and Ta-substituted CaSi_1−*x*_Ta_*x*_O_3_ (*x* = 12.5% and 25%) as a function of photon energy. Ta substitution smooths the dielectric spectra and modifies the dispersive and absorptive responses relative to the pristine phase.

As shown in Fig. 11(a and b), pristine CaSiO_3_ exhibits a stronger dielectric response in the higher-energy region, which is consistent with its wide-band-gap semiconducting nature and dominant O-2p to Si-derived conduction-band transitions. After Ta substitution, the dielectric spectra are noticeably modified because Ta introduces 5d-derived donor states near the Fermi level, which alter the available interband transition pathways and the screening response near the Fermi level. The reduction in *ε*_1_(*ω*) for the doped systems indicates a change in electronic polarizability caused by Ta–O hybridization and charge redistribution, while the modified *ε*_2_(*ω*) spectra reveal changes in the interband transition strength. At higher Ta concentration, the dielectric response becomes smoother and more broadened, suggesting enhanced carrier delocalization, increased electronic screening, and stronger Ta-5d/O-2p orbital interaction. These spectra describe the finite-frequency interband response and do not redefine the ground-state electronic character established in Section 3.4. Because the present OPTIC treatment evaluates transitions between occupied and unoccupied states and does not include an explicit intraband Drude term, weak low-energy interband intensity in the Ta-substituted systems is not evidence of conventional semiconducting behavior.

The real and imaginary parts of *ε*(*ω*) were further used to derive other optical parameters, including the refractive index *n*(*ω*), extinction coefficient *k*(*ω*), reflectivity *R*(*ω*), absorption coefficient *α*(*ω*), and energy-loss function *L*(*ω*). A comparison between pristine and Ta-doped structures shows that increasing Ta concentration strongly affects charge excitation, interband transitions, dielectric screening, and optical absorption behavior. These results demonstrate that Ta-induced electronic states can tune the optical response of CaSi_1−*x*_Ta_*x*_O_3_, linking the spectral changes to the composition-dependent electronic reconstruction.^100,101^

7 *ε*(*ω*) = *ε*_1_(*ω*) + *iε*_2_(*ω*)

The imaginary part of the dielectric function was calculated by considering the dipole transition matrix elements throughout the first Brillouin zone, based on direct electronic transitions between the occupied valence bands and unoccupied conduction bands [eqn (8)]. The optical properties were then evaluated over the photon-energy range of 0–14 eV, allowing a detailed assessment of the material response to photons with different energies. This approach provides insight into how the electronic band structure, orbital transitions, and joint density of states govern the calculated optical response.^102,103^ In the present study, these spectra are used to characterize composition-dependent optical behavior rather than to establish suitability for a specific optoelectronic application.8
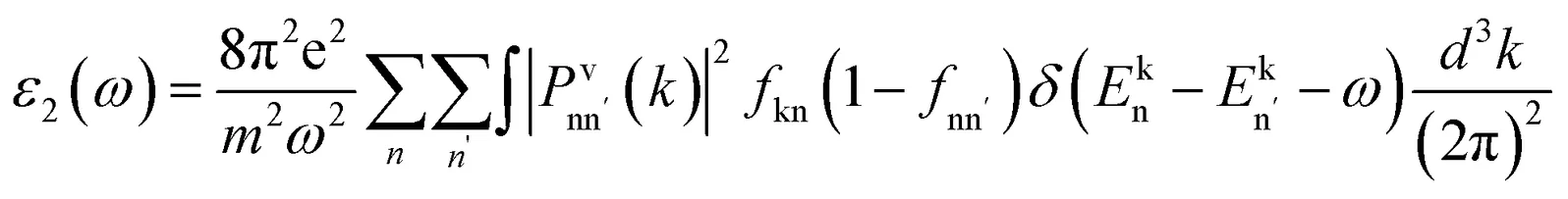


The dipole transition matrix elements, calculated using eqn (8), were used to evaluate interband electronic transitions from occupied valence states to unoccupied conduction states.

9 *M*_cv_ = *µ*_ck_|*δ*.∇*µ*_vk_

The real part of the dielectric function was obtained from the imaginary component using the Kramers–Kronig relations, as expressed in eqn (10). The combined analysis of *ε*_1_(*ω*) and *ε*_2_(*ω*) provides a complete description of the dielectric response of the material, including both electronic polarization and interband absorption processes. These components were further used to calculate key frequency-dependent optical parameters, including the electron energy-loss function *L*(*ω*), refractive index *n*(*ω*), reflectivity *R*(*ω*), extinction coefficient *k*(*ω*), optical conductivity *σ*(*ω*), and absorption coefficient *α*(*ω*). These quantities were evaluated using the standard expressions given in eqn (7)–(18), providing a comprehensive description of the linear optical behavior of the investigated systems over the selected energy range.10
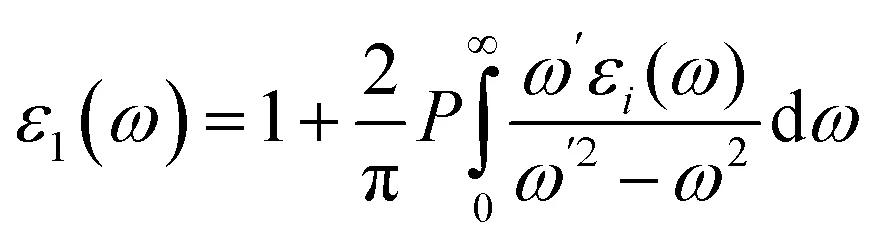

11 *η̃* = *η*(*ω*) + *ik*(*ω*)

12
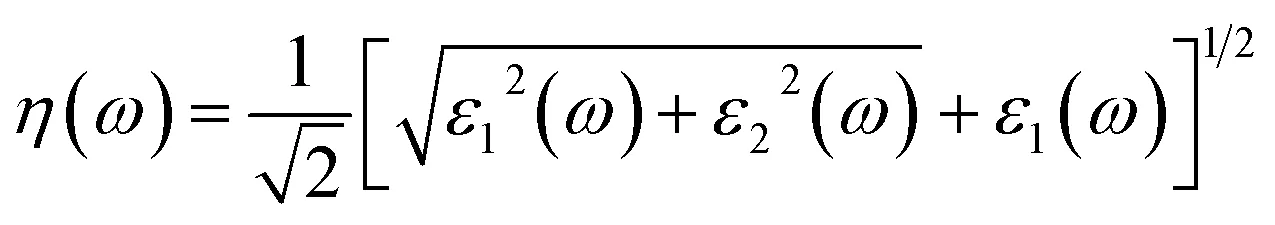

13 *ε*_2_(*ω*) = *η*^2^(0)

14
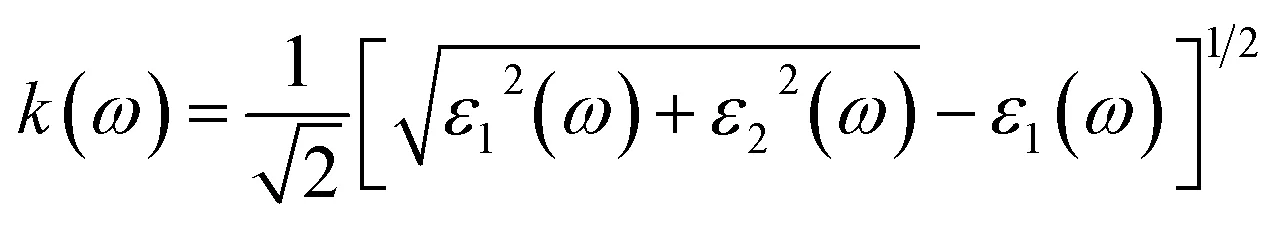
15
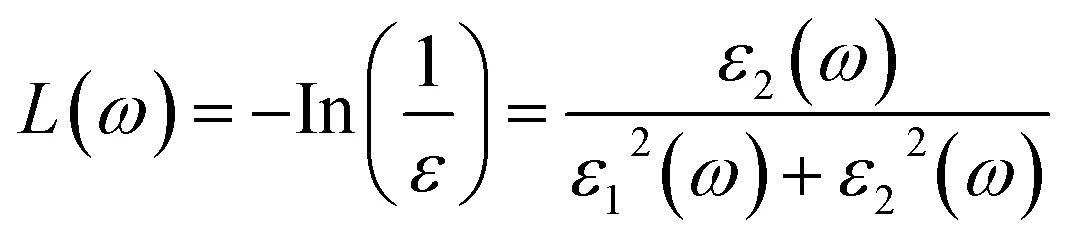
16
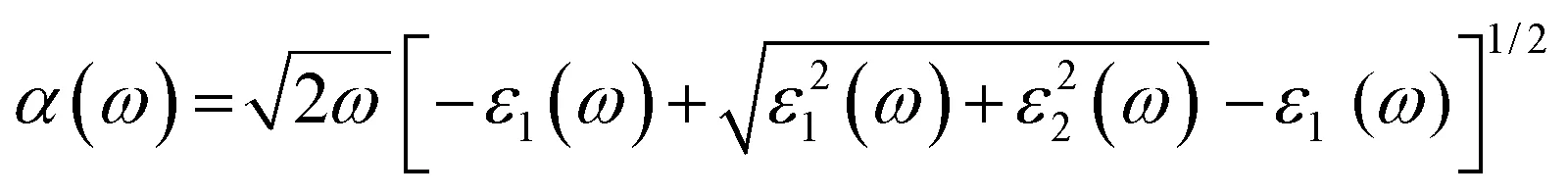
17
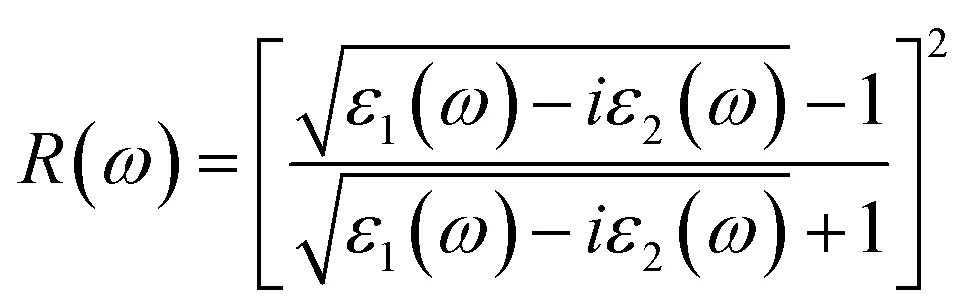
18
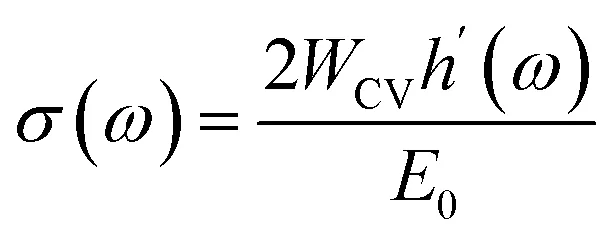


Optical response in the pure CaSiO_3_ system is isotropic with the overlap of three components of *ε*^*xx*^, *ε*^*yy*^, and *ε*^*zz*^, between the energy ranges of 0 to 14 eV. The same type of isotropic behavior is maintained in CaSi_1−*x*_Ta_*x*_O_3_ at 12.5% Ta replacement at higher Ta concentration (25%), the deviation becomes more pronounced, where the *ε*^*zz*^ component differs from *ε*^*xx*^ and *ε*^*yy*^, indicating the emergence of optical anisotropy at higher doping levels. Here, *ε*_1_(*ω*) is interpreted as an optical-response quantity; its sign over the calculated energy range is not used to assign semiconductor or metallic character, which is determined from the Fermi-level band crossings, DOS(*E*_F_), and Fermi-surface topology in Section 3.4. The imaginary part, *ε*_2_(*ω*), shows an enhanced peak intensity with increasing Ta content, suggesting stronger interband electronic transitions. The energy-loss function, *L*(*ω*), increases at higher photon energies, while the reflectivity, *R*(*ω*), decreases, reflecting plasmon-related excitation and reduced reflective response in the high-energy region. In addition, the refractive index, *n*(*ω*), and extinction coefficient, *k*(*ω*), increase in the low-energy region, indicating stronger light–matter interaction with increasing Ta concentration. The reported optical conductivity, *σ*(*ω*), is the interband contribution obtained from the present optical calculation and is distinct from the dc/intraband free-carrier response of the Ta-substituted compositions. The spectra show that pristine and lightly doped CaSiO_3_ largely preserve isotropic optical behavior, whereas higher Ta incorporation induces optical anisotropy, enhanced dielectric activity, and stronger photon-induced electronic response. Taken together, these optical quantities track changes in screening, spectral weight, and interband transitions across the three compositions without altering the ground-state electronic classification.

#### 3.5.1. Complex dielectric function (real and imaginary parts)

The real part of the dielectric function, *ε*_1_(*ω*), shown in Fig. 11(a), provides information about the electronic polarization response of the material under an external electromagnetic field and reflects the influence of band structure on dielectric dispersion and plasmon-related behavior.^104,105^ In pristine CaSiO_3_ and Ta-substituted CaSi_1−*x*_Ta_*x*_O_3_, the low-energy optical response is mainly governed by O-2p-derived valence states and Si/Ta-derived conduction states. For pristine CaSiO_3_, the relatively smooth and higher *ε*_1_(*ω*) response is consistent with its wide-band-gap semiconducting nature and stronger bound-electron polarization. After Ta substitution, the *ε*_1_(*ω*) values become slightly lower at low photon energies, indicating that Ta incorporation modifies the band-edge electronic structure and reduces the overall polarization strength. This reduction can be associated with Ta-5d donor states, enhanced electronic screening, and increased carrier delocalization, which weaken the purely bound-electron dielectric response. The weak oscillatory features observed in the doped systems further suggest that Ta-5d/O-2p hybridized states actively contribute to the dielectric response. This trend agrees with reports on Ta-doped perovskites, where Ta incorporation introduces additional charge carriers, enhances screening, and alters the low-energy dielectric behavior.^106^

In the visible-energy range (1.5–3.0 eV), the real dielectric function of the Ta-substituted systems at both 12.5% and 25% concentrations and in both spin channels shows only minor deviations from pristine CaSiO_3_. This indicates that Ta incorporation has limited influence on low-energy optical polarization and near-edge electronic transitions. However, above approximately 3 eV, the doped curves begin to overlap with each other and gradually deviate from pristine CaSiO_3_, indicating the increasing contribution of Ta-induced electronic states to the dielectric response. At higher photon energies (∼5–10 eV), the Ta-doped systems show a smoother and less pronounced *ε*_1_(*ω*) profile, which can be attributed to Ta-5d/O-2p hybridization, modified interband transitions, and redistribution of spectral weight. Across this spectral range, Ta acts as a donor dopant that mainly modifies the conduction-band electronic structure, producing clear changes in *ε*_1_(*ω*) at higher photon energies while leaving the near-edge visible response relatively less affected. This behavior is consistent with transition-metal-doped perovskites, where dopant-derived d states mainly influence the high-energy dielectric response while producing only limited changes in the visible region.^107^

The *ε*_1_(*ω*) spectra of all compositions gradually decrease with increasing photon energy. The Ta-doped systems enter the negative *ε*_1_(*ω*) region earlier, around 12–13 eV, compared with pristine CaSiO_3_. This earlier zero-crossing indicates an enhanced plasmon-like response, arising from collective oscillations of valence electrons influenced by Ta-induced conduction-band states. Pristine CaSiO_3_ shows a slower and weaker negative shift, reflecting a less pronounced high-energy collective electronic response rather than a directly inferred free-carrier contribution from these interband spectra. At higher energies, the *ε*_1_(*ω*) spectra of all systems begin to overlap, suggesting that the dielectric response becomes dominated by deeper electronic states, where the specific influence of Ta doping becomes less pronounced.

The effective band gap (*E*_g_) is related to the static dielectric constant, *ε*_1_(0), within the Penn model, as represented by eqn (19).^108,109^19
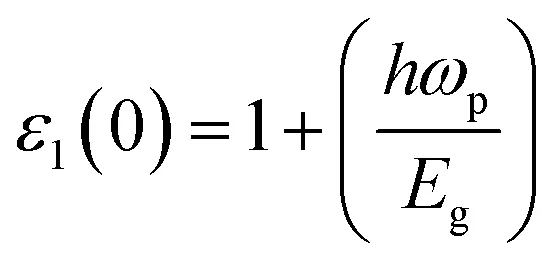


Within the Penn-model framework, the static dielectric constant *ε*_1_(0) is generally related to the effective optical band gap, where a larger band gap usually corresponds to a lower electronic polarizability, while stronger electronic polarizability can enhance *ε*_1_(0). In the present case, pristine CaSiO_3_ shows a relatively stronger low-energy dielectric response, consistent with its stable wide-gap semiconducting nature and well-defined bound-electron polarization. Ta incorporation produces only a slight reduction in *ε*_1_(*ω*) across the visible region, showing that the calculated low-energy interband dielectric response changes only modestly despite the Fermi-level reconstruction identified in the electronic-structure analysis. However, the presence of Ta-derived 5d states modifies the electronic screening and produces nearly overlapping dielectric behavior among the doped compositions above 2–3 eV. At higher photon energies, the 25% Ta-doped system approaches the negative *ε*_1_(*ω*) regime more strongly, indicating an enhanced contribution from collective electronic excitations. Across the calculated spectrum, pristine CaSiO_3_ retains a stronger intrinsic dielectric response in the low-energy region, whereas Ta doping enables progressive tuning of polarization, screening, and plasmon-related optical behavior at higher energies without strongly disturbing the near-edge optical response.

The imaginary part of the dielectric function, *ε*_2_(*ω*), represents the ability of a material to absorb incident electromagnetic radiation and convert it into electronic excitations.^104,110^ As shown in Fig. 11(b), pristine CaSiO_3_ exhibits very low *ε*_2_ (*ω*) below approximately 4 eV, indicating weak absorption in the low-energy region and confirming its wide-band-gap character. The absorption onset appears around 5–6 eV, followed by strong high-energy peaks that can be attributed mainly to interband transitions from O-2p valence states to Si/Ca-derived conduction states. In contrast, the Ta-doped systems at 12.5% and 25%, in both spin channels, show weaker and broader absorption features, with strong spectral overlap. This indicates that Ta incorporation modifies the conduction-band states through Ta-5d orbital contributions. For the Ta-doped compositions, *ε*_2_(*ω*) remains lower than that of pristine CaSiO_3_ over most of the investigated energy range, suggesting a reduction in optical absorption strength. In the low-energy range (0–3 eV), the doped curves remain close to zero, indicating that Ta incorporation does not introduce strong additional low-energy interband transitions. Within the interband optical framework defined in Section 3.5, this weak *ε*_2_ response does not contradict the Fermi-level occupation found for the Ta-substituted structures and should not be used to reclassify them as conventional semiconductors. Compared with the intense high-energy peaks of the parent phase, the doped systems show weakened and broadened spectral features between 5 and 10 eV, reflecting a redistribution of oscillator strength caused by Ta-induced modification of the conduction band. The reduced peak intensity suggests that Ta-derived 5d states modify the available transition channels without producing strong localized absorption peaks. Similar behavior has been reported in transition-metal-doped perovskites, where dopant-induced electronic states can weaken absorption intensity and modify the dielectric response.^111,112^ The strong overlap between the 12.5% and 25% Ta-doped spectra further suggests that both concentrations produce comparable optical behavior governed mainly by Ta-related electronic states. In the high-energy region, pristine CaSiO_3_ shows larger *ε*_2_(*ω*) peaks at higher photon energies, indicating stronger allowed interband transitions and a more intense optical response. By comparison, the Ta-doped systems exhibit lower, smoother, and less fluctuating absorption profiles, which indicate reduced transition probability and redistribution of oscillator strength due to Ta-induced changes in the conduction-band region. The nearly identical *ε*_2_(*ω*) profiles of the doped compositions suggest that increasing Ta concentration does not create separate high-intensity absorption channels but instead stabilizes a similar dielectric response. These trends establish a clear contrast between the stronger high-energy interband response of pristine CaSiO_3_ and the weaker, smoother response of the Ta-substituted compositions. The *ε*_2_(*ω*) spectra are therefore interpreted as composition-dependent optical descriptors and are not used independently to assign suitability for a particular absorption or optoelectronic application.

#### 3.5.2. Optical absorption pathways and electron energy dissipation function

**Fig. 12:**
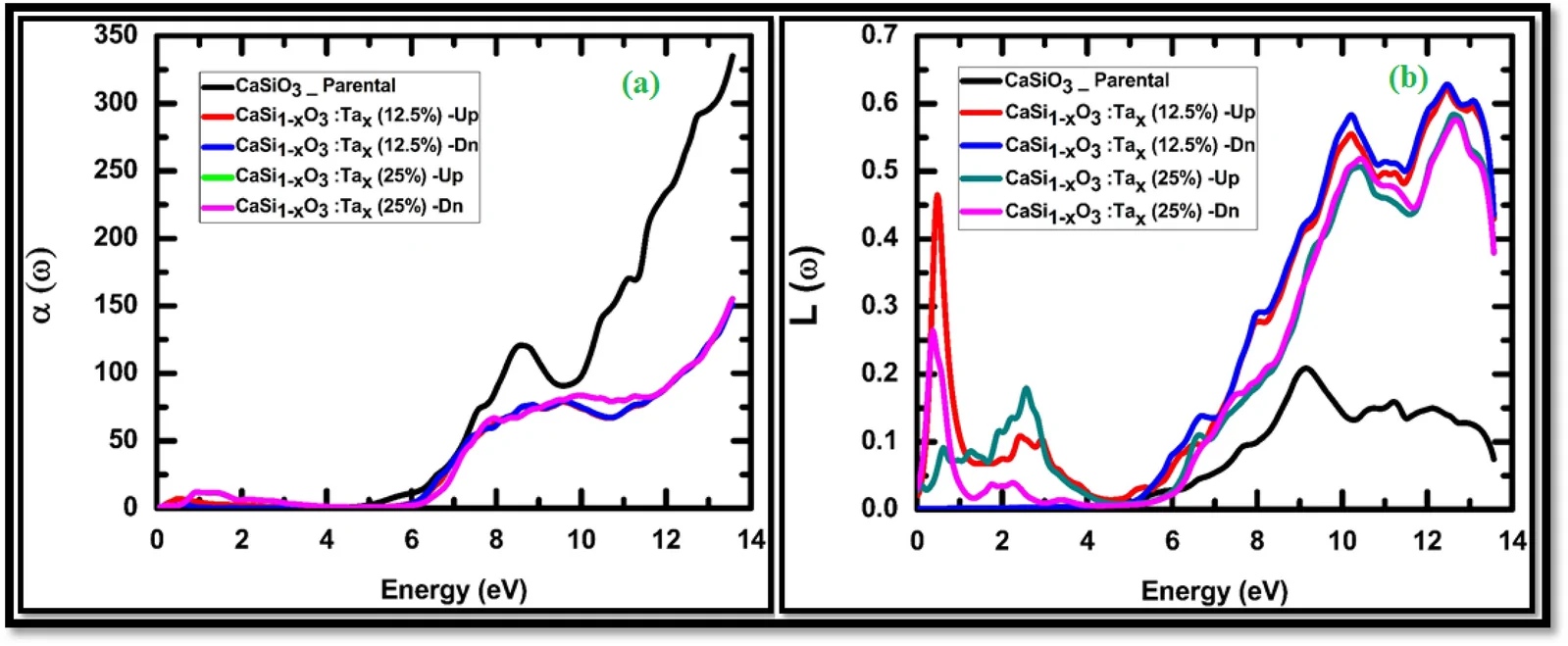
(a) Absorption coefficient *α*(*ω*) and (b) energy-loss function *L*(*ω*) of pristine CaSiO_3_ and Ta-substituted CaSi_1−*x*_Ta_*x*_O_3_ (*x* = 12.5% and 25%) as a function of photon energy. Ta substitution reduces and smooths the high-energy interband absorption while broadening and enhancing the energy-loss features mainly in the ∼8–13 eV range.

The absorption coefficient, *α*(*ω*), measures how effectively a material absorbs incident electromagnetic radiation and converts photon energy into interband electronic excitations.^99,106^ As shown in Fig. 12(a), pristine CaSiO_3_ exhibits a pronounced absorption response, with *α*(ω) rising sharply around 6 eV and becoming highly intense in the deep-ultraviolet region. This behavior is typical of wide-band-gap oxides and is mainly associated with interband transitions from O-2p valence states to higher-lying Si/Ca-derived conduction states.^113^ In contrast, the Ta-doped compositions at 12.5% and 25% show a similar absorption onset but display significantly lower and smoother *α*(*ω*) values over most of the investigated energy range. In the low-energy region (0–4 eV), the calculated interband absorption remains negligible for all systems; for the Ta-substituted compositions, this spectral minimum describes the interband channel and does not supersede the Fermi-level electronic classification established in Section 3.4. Between 6 and 10 eV, the doped curves increase more gradually and appear smoother than the parent phase, suggesting that Ta incorporation redistributes the conduction-band states and reduces the probability of strong interband transitions. At higher photon energies (>10 eV), pristine CaSiO_3_ shows a rapid increase in absorption intensity, whereas the Ta-doped systems exhibit a more moderate increase. This indicates a partial suppression of high-energy transition channels, likely due to Ta-5d/O-2p hybridization, enhanced electronic screening, and redistribution of oscillator strength. The close overlap between the 12.5% and 25% Ta-doped curves suggests that moderate Ta incorporation produces a similar modification of the optical-transition landscape. Similar smoothing and reduction of high-energy absorption features have been reported in doped oxide perovskites, where dopant-induced changes in band structure and carrier screening modify the absorption response.^114^

Pristine CaSiO_3_ shows the strongest ultraviolet absorption, indicating a higher probability of allowed interband transitions. By comparison, the Ta-substituted systems exhibit weaker and more stable absorption profiles, reflecting a reduced transition probability and a more controlled optical response. Accordingly, the calculated *α*(*ω*) spectra demonstrate a composition-dependent change in ultraviolet and high-energy interband absorption. These spectral differences are not, by themselves, used to rank the compositions for UV-absorption, dielectric, or other optical applications, which would require application-specific evaluation and experimental validation.

Fig. 12(b), presents the electron energy-loss function, *L*(*ω*), which describes the energy lost by fast electrons while passing through the material and provides insight into dielectric screening, collective electronic oscillations, and plasmon-related excitations.^96,115^ For pristine CaSiO_3_, *L*(*ω*) shows a relatively weak and broad response, with only limited features in the high-energy region. This indicates weak collective electronic oscillations and low energy-dissipation behavior. In contrast, Ta incorporation significantly enhances *L*(*ω*) and redistributes the loss spectrum over different energy ranges. In the low-energy region, especially for the 12.5% Ta-doped system, sharp loss features appear, showing additional dielectric-loss channels associated with Ta-derived states. Because *L*(*ω*) is derived from the calculated dielectric response, these peaks are not used here as an independent measure of free-carrier density or metallicity. In the infrared-visible region (∼1–3 eV), pristine CaSiO_3_ remains nearly lossless, whereas the Ta-doped compositions show finite loss features. This indicates the opening of additional dissipation channels, caused by Ta-induced interband coupling and enhanced electronic polarizability. At higher photon energies (>6 eV), the Ta-doped systems exhibit broader and stronger maxima over the ∼8–13 eV range, which can be assigned to plasmon-like excitations enhanced by electronic screening and conduction-band reconstruction. The increased peak intensity reflects stronger collective electron oscillations, while the broader peak shape suggests greater damping and overlap of multiple excitation pathways. The close similarity between the 12.5% and 25% Ta-doped spectra indicates that moderate Ta incorporation produces a comparable excitation landscape.

These results agree with reports on donor-doped oxide perovskites, where dopant-induced carriers modify the dielectric response and enhance high-energy loss features.^111^ They are also consistent with electron energy-loss spectroscopy studies, where donor states generate stronger and more structured plasmonic peaks.^116^ The calculated loss spectra therefore identify composition-dependent differences in dielectric-loss and plasmon-related response. Possible relevance to dielectric, plasmonic, radiation-interaction, or high-energy electronic technologies requires application-specific analysis and experimental validation and is not established from *L*(*ω*) alone.

#### 3.5.3. Reflectivity dispersion and optical conductivity scaling features

**Fig. 13:**
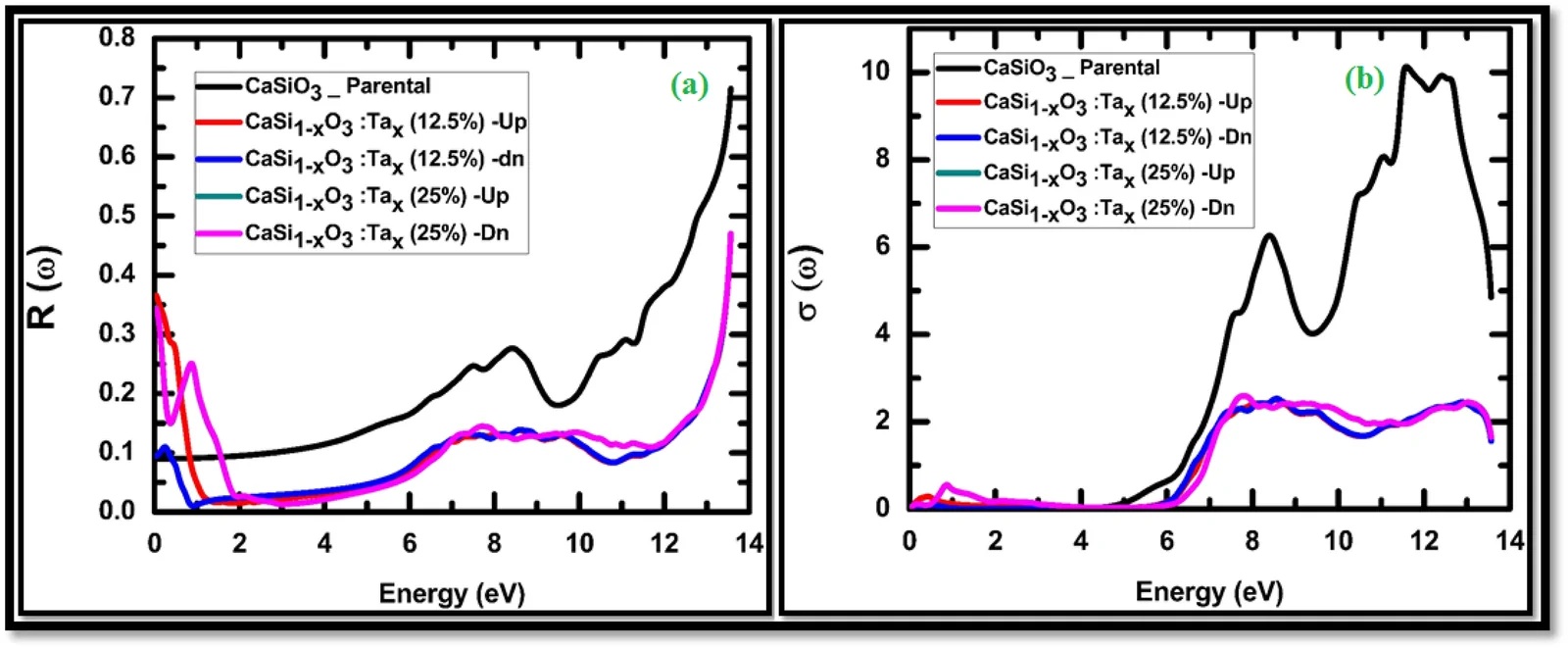
(a) Reflectivity *R*(*ω*) and (b) optical conductivity *σ*(*ω*) of pristine CaSiO_3_ and Ta-substituted CaSi_1−*x*_Ta_*x*_O_3_ (*x* = 12.5% and 25%) as a function of photon energy. Ta substitution smooths the high-energy interband reflectivity and optical-conductivity features relative to pristine CaSiO_3_, with broader and less intense spectral features in the doped compositions.

Reflectivity, *R*(*ω*), represents the fraction of incident electromagnetic radiation reflected from the material surface and is an important indicator of light–surface interaction, dielectric response, and near-surface electronic behavior.^102,117^ As shown in Fig. 13(a), pristine CaSiO_3_ exhibits low and nearly smooth reflectivity in the infrared-to-visible region (<3 eV), indicating weak low-energy reflectance within the calculated optical response. In contrast, Ta incorporation at 12.5% and 25% introduces additional low-energy reflectivity features, which can be attributed to dopant-induced electronic states and enhanced low-energy polarization.

In the intermediate energy range (3–8 eV), the reflectivity gradually increases for all compositions. However, the pristine system maintains a relatively stronger response, whereas the Ta-doped profiles become smoother and nearly overlapping, suggesting redistribution and damping of interband transition strength. At higher photon energies (>8 eV), pristine CaSiO_3_ shows a sharp increase in reflectivity with clear oscillatory features, indicating strong high-energy interband transition activity. By contrast, the Ta-doped systems exhibit broader and weaker reflectivity features, which may be associated with enhanced electronic screening, Ta-derived states, and increased damping of optical transitions. The high-intensity peaks are linked to stronger transition probability, while the flatter regions indicate a more stable and moderated optical response. This behavior is consistent with wide-band-gap oxide perovskites, where low-energy reflectance is generally weak and ultraviolet-region reflectivity is governed mainly by transitions from O-2p valence states to higher-energy cation-derived conduction states.^111^ Donor doping modifies this response by redistributing electronic states, reducing sharp spectral fluctuations, and lowering transition strength.^112^ The moderated reflectivity of the Ta-substituted systems is therefore treated here as a calculated spectral characteristic, while pristine CaSiO_3_ retains the stronger high-energy reflectance response. Its possible relevance to optical coatings or oxide-electronic interfaces would require application-specific assessment beyond the calculated *R*(*ω*) spectra.

The optical conductivity, *σ*(*ω*), describes the ability of a material to support photon-induced electronic excitations and is directly governed by its electronic band structure and the availability of allowed transition channels.^118,119^ Here, *σ*(*ω*) denotes the frequency-dependent interband optical conductivity and is distinct from the dc-like *σ*/*τ* obtained from the BoltzTraP transport analysis. As shown in Fig. 13(b), pristine CaSiO_3_ exhibits very low optical conductivity at lower photon energies (<4 eV), consistent with its wide-gap electronic structure and the absence of strong low-energy interband transitions. With increasing photon energy, *σ*(*ω*) rises sharply after the absorption threshold and develops strong peaks, indicating enhanced transition activity mainly associated with excitations from O-2p valence states to higher-energy Si/Ca-derived conduction states.

After Ta incorporation at 12.5% and 25%, in both spin channels, the overall spectral shape is retained, but the intensity becomes significantly lower and the features become broader and smoother. In the 6–10 eV range, the Ta-doped systems show broad but less intense conductivity peaks, suggesting that Ta substitution redistributes the electronic states and reduces the probability of strong interband transitions. At higher energies (>10 eV), pristine CaSiO_3_ exhibits strong oscillatory conductivity, whereas the doped systems show a more damped and flattened response. This behavior indicates enhanced electronic screening, reduced oscillator strength, and suppression of high-energy transition channels caused by Ta-derived states. The near overlap of the 12.5% and 25% Ta-doped curves suggests that increasing Ta content within this range does not strongly alter the overall optical-conductivity profile. This trend is consistent with donor-modified oxide perovskites, where additional dopant-induced electronic states can reorganize the band structure and modify high-energy conductivity through screening and spectral-weight redistribution.^111,114^ The lower interband optical-conductivity amplitude of the Ta-substituted systems describes a more moderated photon-driven response and should not be interpreted as lower dc transport conductivity, particularly for the 25% Ta composition.

#### 3.5.4. Refractive index, extinction coefficient, and phase-loss coupling framework

**Fig. 14:**
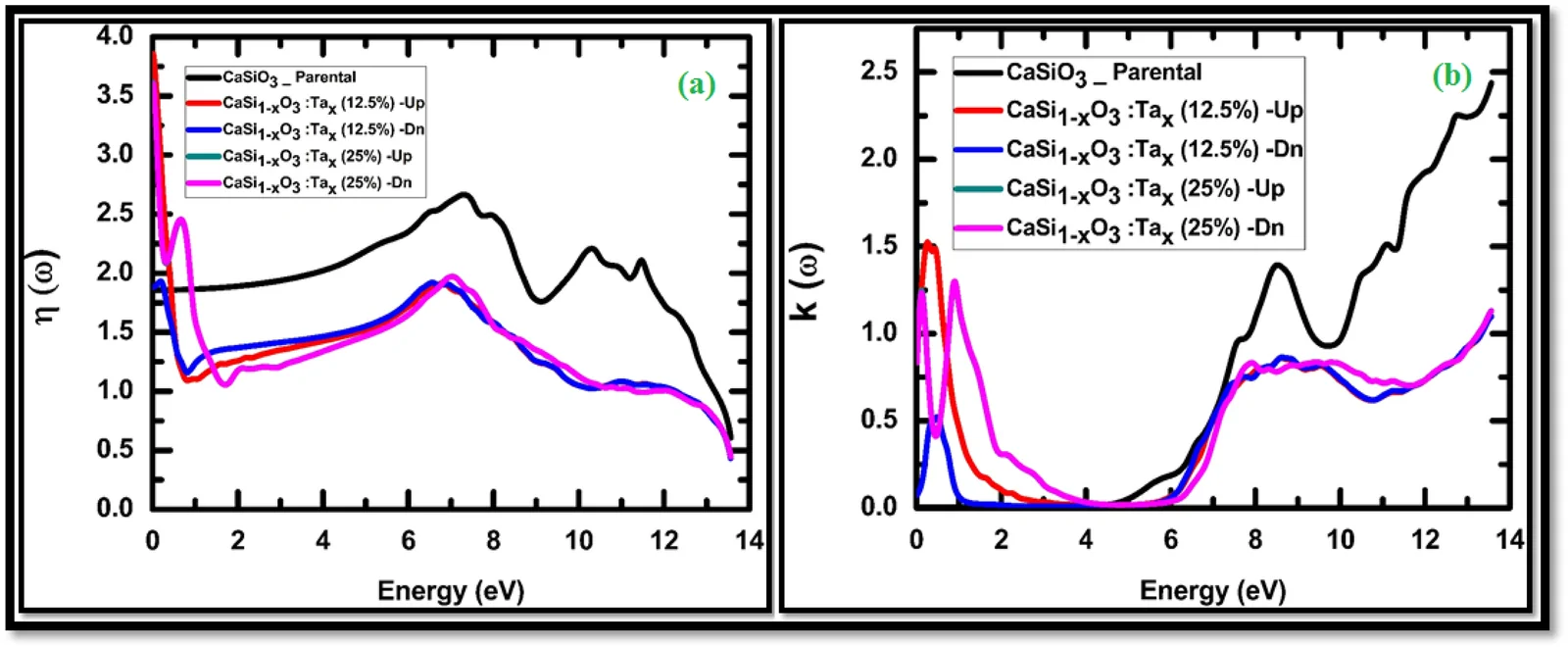
(a) Refractive index *n*(*ω*) and (b) extinction coefficient *k*(*ω*) of pristine CaSiO_3_ and Ta-substituted CaSi_1−*x*_Ta_*x*_O_3_ (*x* = 12.5% and 25%) as a function of photon energy. Ta incorporation leads to smoother dispersion and reduced spectral variability, with broader high-energy features and low extinction in the visible region for the Ta-substituted compositions.

The refractive index, *n*(*ω*), describes how electromagnetic waves propagate through a material and is strongly governed by the material's electronic polarization response.^120^ As shown in Fig. 14(a), pristine CaSiO_3_ exhibits a relatively higher *n*(*ω*) at low photon energies, followed by a gradual variation and broad features at higher energies. This behavior indicates stronger bound-electron polarization and active interband-transition contributions in the parent compound. After Ta incorporation at 12.5% and 25%, the refractive-index response changes noticeably. At very low photon energies, *n*(*ω*) decreases more rapidly, while the doped systems show a more uniform dispersion profile over the infrared-visible range (1–5 eV). A moderate peak appears around 6–7 eV in the Ta-doped systems, which can be associated with interband transition activity involving Ta-5d/O-2p hybridized states. At higher photon energies, *n*(*ω*) gradually decreases as the contribution of electronic polarization becomes weaker. The close similarity between the 12.5% and 25% Ta-doped curves suggests that moderate Ta substitution does not strongly alter the overall refractive-index dispersion profile. In general, a higher *n*(*ω*) is associated with stronger electronic polarization and optical transition strength, whereas a smoother and less fluctuating profile indicates reduced spectral variation in the calculated refractive response. The contrast in *n*(*ω*) therefore establishes a composition-dependent change in dispersion and electronic polarization. Possible use of these characteristics in optical coatings, dielectric layers, or related optical components requires separate application-specific evaluation and is not inferred from the refractive-index spectra alone. These findings are consistent with donor-modified perovskite systems, where dopant-controlled electronic states influence refractive-index behavior and polarization properties.^111,121^

The extinction coefficient, *k*(*ω*), measures the attenuation of electromagnetic waves inside a material due to absorption and energy-dissipation processes.^122^ As shown in Fig. 14(b), *k*(*ω*) is strongly dependent on photon energy and the availability of electronic transition channels. For pristine CaSiO_3_, *k*(*ω*) remains negligible at lower photon energies (<4 eV), indicating minimal optical loss and very weak absorption in the low-energy region. With increasing photon energy, *k*(*ω*) rises sharply, and intense peaks appear in the ultraviolet region. These peaks are associated with strong interband transitions from O-2p valence states to higher-energy Si/Ca-derived conduction states. In the Ta-substituted systems at 12.5% and 25%, in both spin channels, distinct low-energy features appear in *k*(*ω*) due to dopant-induced states that create additional absorption channels.

In the visible region, however, the spectra remain close to zero and nearly flat, indicating low extinction and weak calculated interband attenuation in this spectral range. At higher energies (∼6–10 eV), the peaks become broader and less intense than those of the parent compound, suggesting redistribution of transition strength and enhanced electronic screening. Even above 10 eV, pristine CaSiO_3_ shows a steeper and more structured increase in *k*(*ω*), whereas the doped systems exhibit a more restrained and strongly overlapping response, reflecting similar electronic behavior at both Ta concentrations. Physically, strong *k*(*ω*) peaks correspond to intense absorption processes and high transition probability, while flatter curves indicate lower calculated attenuation over the corresponding energy interval. Comparable substituted oxide perovskites likewise exhibit composition-dependent changes in electronic structure, optical attenuation, absorption, and charge-transport response.^123–126^ The low visible-region interband extinction of the Ta-substituted compositions and their modified high-energy response are therefore reported as calculated spectral characteristics. Suitability for coatings, window layers, ultraviolet filtering, or photonic spectral-control functions cannot be established from *k*(*ω*) alone and requires application-specific modelling and experimental assessment.

Table 7 condenses the spectral trends discussed in Sections 3.5.1–3.5.4. Pristine CaSiO_3_ retains sharper high-energy interband features, whereas Ta substitution broadens or suppresses several calculated optical structures and modifies dielectric screening. Because these quantities were obtained within the OPTIC interband framework, the summary is restricted to the frequency-dependent optical response and is not used to redefine the electronic classification established from the band structure, DOS, and Fermi-surface results.

**Table 7:** Summary of the frequency-dependent optical response of pristine and Ta-doped CaSiO_3_, comparing the calculated interband optical descriptors, spectral evolution, electronic origin, and spectral significance^a^

Optical descriptor	CaSiO_3__(parent)	CaSi_1−*x*_Ta_*x*_O_3_ (*x* = 12.5%)	CaSi_1−*x*_Ta_*x*_O_3_ (*x* = 25%)	Spectral evolution	Electronic origin	Spectral significance
*ε*_1_(*ω*)	High intrinsic polarization; delayed plasmon crossover	Moderate suppression; earlier crossover	Comparable low-energy response; earlier high-energy crossover	Smoother dispersion; reduced amplitude	Donor-induced screening; conduction-band reshaping	High-energy dielectric and plasmon-related response modulation
*ε*_2_(*ω*)	Strong, sharp interband transitions	Reduced intensity; broadened peaks	Nearly identical spectral overlap	Flattened high-energy structure	Oscillator strength redistribution	Controlled interband absorption response
*α*(*ω*)	Intense UV absorption; sharp onset	Reduced magnitude; gradual rise	Consistent suppression	Controlled transition probability	Band-structure modulation	UV-response and absorption-profile modulation
*L*(*ω*)	Weak collective excitation features	Enhanced and sharper peaks	Broader and damped maxima	Strengthened plasmon resonance	Ta-induced screening and collective electronic excitations	Plasmon-related and electron-energy-loss response
*R*(*ω*)	Low IR-visible reflectance; strong UV rise	Smoothed response; reduced oscillations	Similar damped profile	Reduced reflectivity fluctuations	Screening and transition damping	Reduced high-energy reflectance response
*σ*(*ω*)	Sharp high-energy conductivity peaks	Lower intensity; broadened features	Consistent reduction	Diffuse spectral profile	Reduced interband transition strength	Controlled photon-induced interband response
*n*(*ω*)	High refractive response; strong dispersion	Lower magnitude; uniform profile	Nearly invariant	Stabilized dispersion	Reduced polarization strength	Composition-dependent optical dispersion
*k*(*ω*)	High UV extinction; sharp peaks	Lower intensity; smoother curves	Overlapping spectral response	Suppressed optical attenuation	Enhanced screening and transition control	Low visible-region interband attenuation

a The optical descriptors in this table summarize the calculated frequency-dependent interband response. They are not used as independent criteria for metallicity and do not represent an explicit intraband/Drude contribution; the electronic classification is established from Sections 3.4.1–3.4.3.

### 3.6. Charge-transport dynamics and thermoelectric energy conversion response

The transport properties of pristine CaSiO_3_ and Ta-substituted CaSi_1−*x*_Ta_*x*_O_3_ at 12.5% and 25% are mainly governed by their underlying electronic band structures. These properties were calculated using the BoltzTraP formalism within the rigid-band approximation and semiclassical transport theory.^124^ The key thermoelectric parameters, including electrical conductivity, electronic thermal conductivity, Seebeck coefficient, and power factor (PF), are strongly interrelated and must be analyzed together to evaluate thermoelectric behavior. In general, an efficient thermoelectric material requires a large Seebeck coefficient, high electrical conductivity, and low total thermal conductivity, which represent a balance between carrier transport and heat dissipation. The variation of these parameters across the studied compositions provides direct insight into how Ta incorporation modifies the electronic structure, carrier concentration, transport pathways, and composition-dependent thermoelectric performance.

#### 3.6.1. Synergistic charge–heat transport dynamics and conductivity scaling

Under the constant relaxation time approximation, the electrical conductivity is represented by the equation below, eqn (20),20
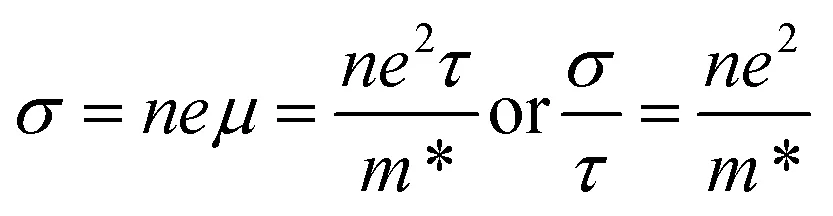


**Fig. 15:**
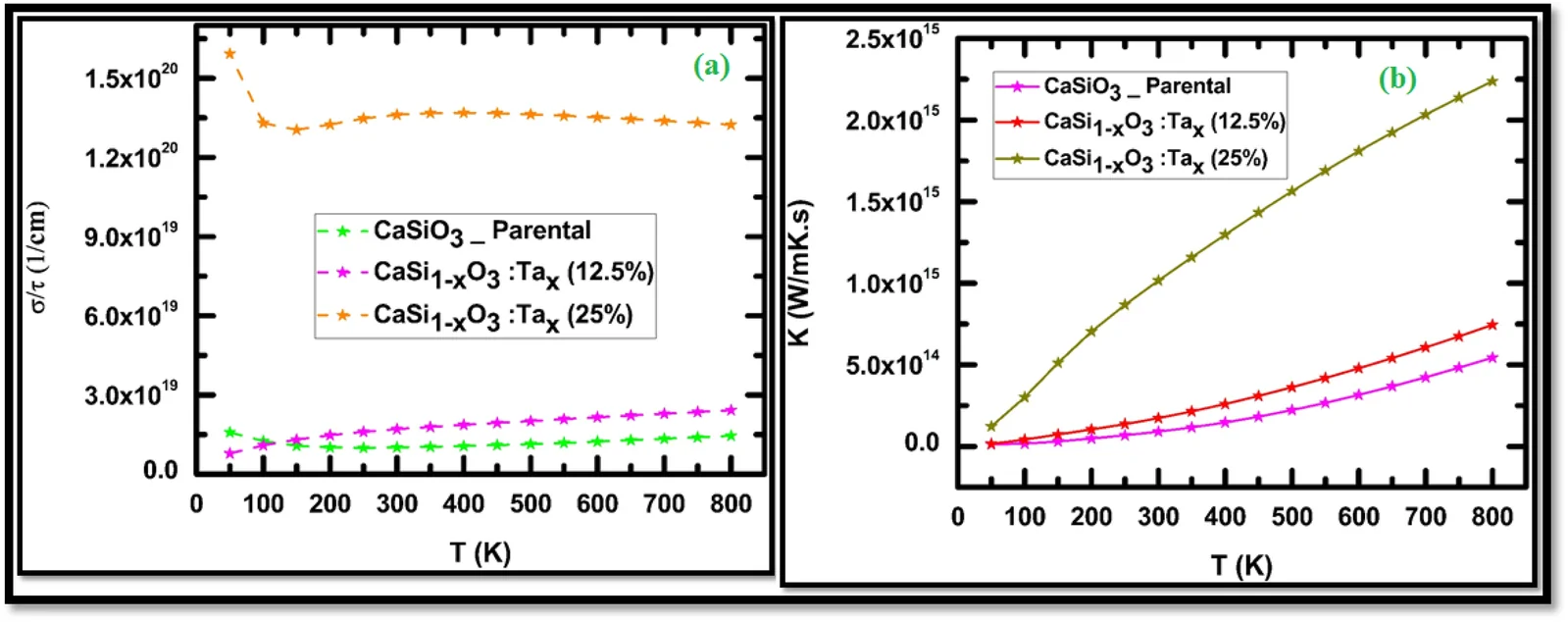
(a and b). Temperature-dependent *σ*/*τ* and electronic thermal-transport response of pristine and Ta-doped CaSi_1−*x*_Ta_*x*_O_3_ within the constant–relaxation–time BoltzTraP framework. Ta substitution increases the band-derived charge and electronic heat transport most strongly at 25% substitution; the thermal curve does not include an independently calculated lattice contribution.

Within the constant-relaxation-time approximation, *σ*/*τ* is governed by the band velocities and energy-dependent transport distribution; it should not be treated as a direct, independent measurement of carrier concentration, mobility, or effective mass. Fig. 15(a) shows a very small and almost temperature-independent *σ*/*τ* of crystalline CaSiO_3_ that is consistent with weak intrinsic transport in the wide-gap parent state. The electrical conductivity divided by relaxation time, *σ*/*τ*, reflects the ability of charge carriers to move through the material and is strongly controlled by carrier concentration, band dispersion, and dopant-induced states. As shown in Fig. 15(a), pristine CaSiO_3_ exhibits a low and weakly temperature-dependent *σ*/*τ*, confirming its insulating transport behavior and limited carrier availability. A similar trend is observed for the 12.5% Ta-substituted system, where *σ*/*τ* increases only slightly with temperature. The comparatively small *σ*/*τ* at 12.5% Ta should not be interpreted as evidence of an insulating state or insufficient carrier activation. Instead, it shows that the occupied near-*E*_F_ states identified by the electronic-structure analysis produce only a modest band-derived transport weight within the present constant-*τ* treatment.

In contrast, the 25% Ta-doped composition shows a much higher *σ*/*τ* value, with only weak temperature dependence at higher temperatures. This larger response is consistent with carrier-dominated transport in the metallic 25% composition, where Ta-derived bands crossing *E*_F_ provide additional conducting channels. The nearly flat profile at high temperature suggests that conduction is mainly controlled by the availability of Ta-induced carriers rather than by strong thermally activated processes. Physically, the enhancement is better associated with the increased Ta-derived band weight at and across *E*_F_ than with thermal activation across a semiconductor gap. The increase from pristine CaSiO_3_ to 12.5% Ta and then to 25% Ta reflects concentration-dependent changes in near-*E*_F_ band dispersion and transport weight; *σ*/*τ* alone does not independently determine the free-carrier concentration or mobility. Similar behavior has been reported in donor-doped oxide systems, where aliovalent substitution enhances conductivity through increased carrier density and improved carrier mobility.^127,128^ The calculated *σ*/*τ* trends therefore establish a composition-dependent increase in band-derived charge transport, with the 25% Ta composition representing the most conductivity-dominated regime among the modeled systems. These results characterize the intrinsic transport response within the constant-τ framework and do not, by themselves, establish suitability for a specific oxide-electronic or thermoelectric application; such assessment requires the combined transport descriptors, an experimentally relevant relaxation time, total thermal conductivity, and experimental validation. The BoltzTraP thermal-transport curve is interpreted as the electronic contribution obtained within the same semiclassical constant-relaxation-time framework. The electronic contribution to thermal conductivity is commonly described by the Wiedemann–Franz relation, *κ*_e_ = *LσT*, where *L* is the Lorenz number, *σ* is the electrical conductivity, and *T* is the absolute temperature. When conductivity is reported as *σ*/*τ*, the corresponding electronic thermal-transport quantity follows *κ*_e_/*τ* = *L*(*σ*/*τ*) *T* within this approximation. As shown in Fig. 15(b), pristine CaSiO_3_ exhibits very low thermal conductivity with a weak and nearly linear temperature dependence, which is characteristic of an insulating system with negligible free-carrier contribution to heat transport. A similar but slightly enhanced trend is observed for the 12.5% Ta-doped system, where *κ* increases slowly with temperature, indicating only a modest electronic heat-transport contribution in this calculation; this magnitude does not imply that the 12.5% composition remains electronically insulating. In contrast, the 25% Ta-substituted composition shows a sharp and continuous increase in thermal conductivity over the full temperature range, with values significantly higher than those of the pristine and 12.5% Ta-doped systems. The smooth temperature dependence should be interpreted within the constant-*τ* model and cannot be used to infer the absence of microscopic scattering anomalies or the behavior of the lattice/phonon thermal conductivity.

Because no independent lattice thermal-conductivity calculation is included in this BoltzTraP analysis, the low electronic contribution for pristine and 12.5% Ta cannot by itself establish phonon-dominated total heat transport. However, the steeper slope and larger *κ* value of the 25% Ta-doped system indicate a substantial enhancement of the electronic component of thermal conductivity, consistent with the corresponding increase in *σ*/*τ* for the same composition. This increase tracks the stronger participation of Ta-derived states near and across *E*_F_, which enhances electronic heat transport; it should not be treated as an independent carrier-density measurement. No conclusion about changes in microscopic scattering rates is drawn from the absence of sharp features because the relaxation time is held constant in the present transport model. Compared with donor-doped oxide systems reported in the literature ^127^ where dopant incorporation partially modifies thermal conductivity, the present results show a clear trend: pristine CaSiO_3_ maintains low thermal conductivity, 12.5% Ta substitution causes only a minor change, and 25% Ta substitution produces a marked increase in carrier-assisted thermal transport. The concentration dependence agrees qualitatively with donor-doped oxides in which the electronic heat-transport contribution grows with enhanced electrical conduction;^128^ establishing a crossover between lattice- and carrier-dominated total thermal conductivity would require a separate phonon-transport calculation. The present thermal-transport result is therefore most directly relevant to the electronic heat-conduction penalty in thermoelectric optimization. Claims concerning total thermal-barrier performance require the lattice contribution and are not inferred from the present BoltzTraP data alone.

#### 3.6.2. Carrier-asymmetry-driven seebeck response and power factor optimization

**Fig. 16:**
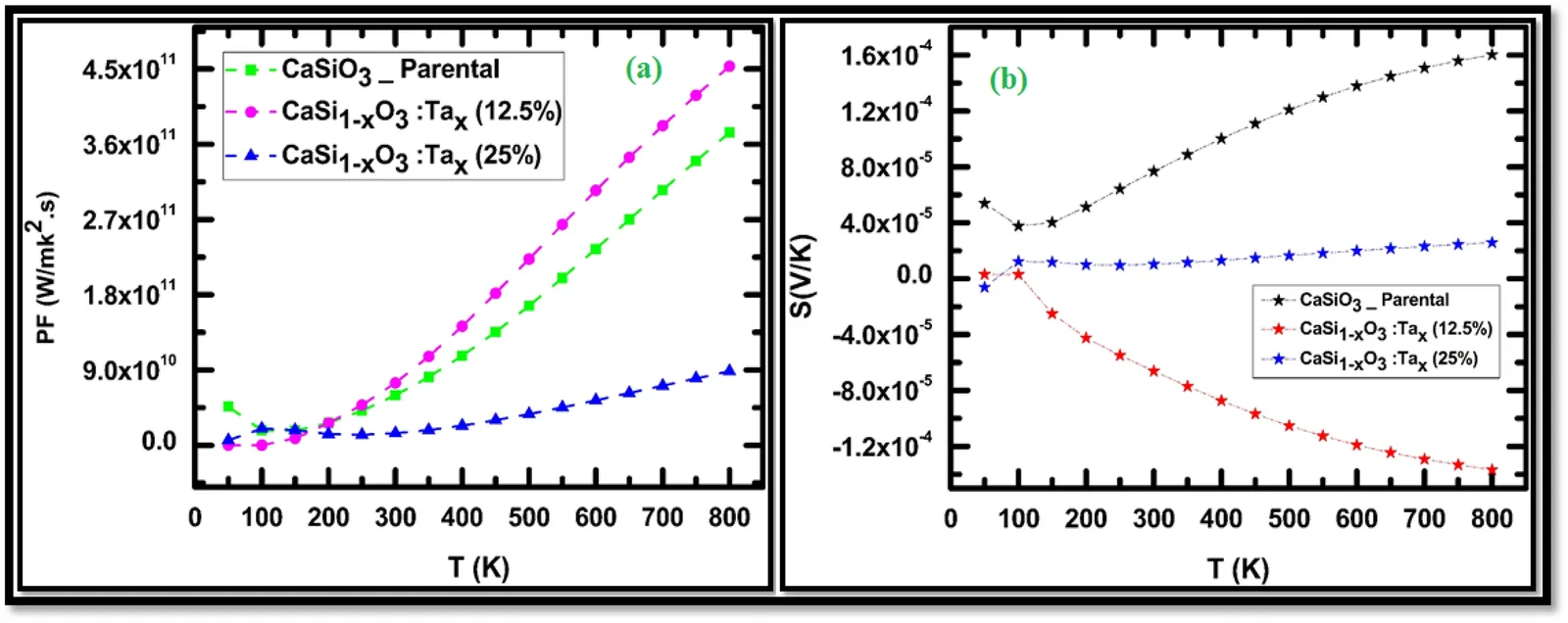
(a and b) Temperature dependence of the power-factor quantity (PF/*τ* within the constant-relaxation-time treatment) and Seebeck coefficient (*S*) for pristine and Ta-doped CaSi_1−*x*_Ta_*x*_O_3_. Ta substitution changes the sign and magnitude of *S* and produces a non-monotonic power-factor response: the 12.5% composition gives the strongest enhancement, whereas the 25% metallic composition remains limited by its small Seebeck magnitude.

The Seebeck coefficient, *S*, represents the thermally induced voltage generated across a material under an applied temperature gradient and is expressed as *S* = Δ*V*/Δ*T*. Its magnitude and sign are strongly governed by the energy-dependent distribution of electronic states near the Fermi level, where the sign of *S* reflects the transport-weighted asymmetry of states around *E*_F_: positive values indicate a hole-like contribution and negative values an electron-like contribution. The sign of *S* should not, by itself, be used to classify a composition as semiconducting or metallic. As shown in Fig. 16(b), pristine CaSiO_3_ exhibits a positive Seebeck coefficient over the entire temperature range, indicating a hole-like transport asymmetry in the calculated thermoelectric response. The gradual increase in *S* with temperature reflects a systematic change in the energy dependence of the transport distribution rather than serving as an independent measure of carrier activation.

For the 12.5% Ta-substituted composition, the Seebeck coefficient becomes negative and decreases further with increasing temperature, which is consistent with electron-dominated n-type thermopower in this composition. The sign change is compatible with the Ta-derived donor states increasing the electron-like contribution to transport near *E*_F_. The smooth decrease indicates a continuous temperature evolution of the calculated transport asymmetry without an abrupt change in sign. In contrast, the 25% Ta-doped system shows a small positive Seebeck coefficient with a nearly linear temperature dependence, but this does not imply a return to semiconducting behavior or require a compensated carrier picture. For a metallic system, a small positive *S* can arise from the energy dependence of the transport distribution and from multiband contributions near *E*_F_. Because the calculations employ the constant–relaxation–time approximation, the temperature dependence of *S* is not used here to infer a temperature-dependent scattering mechanism. The observed signs and magnitudes are governed primarily by the asymmetry of the transport distribution around *E*_F_, together with band velocities and the relative contributions of states close to the Fermi level. The transition from a strong positive Seebeck response in pristine CaSiO_3_ to negative or weakened Seebeck behavior in the Ta-doped systems is consistent with dopant-modified oxide materials, where donor states can alter carrier polarity and reduce the Seebeck magnitude through increased electron contribution.^129,130^ The sign of *S* alone is insufficient to establish practical p-type performance in pristine CaSiO_3_ because its low electrical transport response must also be considered; the Ta-substituted compositions are therefore compared through the combined Seebeck and conductivity trends.

The power factor, expressed as PF = *S*^2^*σ*, is a key parameter for evaluating thermoelectric efficiency because it combines the effects of electrical conductivity and Seebeck coefficient. Within the constant–relaxation–time treatment used here, the directly comparable calculated quantity is PF/*τ* = *S*^2^(*σ*/*τ*) unless a specific relaxation time *τ* is supplied. As shown in Fig. 16(a), the temperature dependence of the power factor for pristine CaSiO_3_ and Ta-substituted CaSi_1−*x*_Ta_*x*_O_3_ is strongly composition-dependent, reflecting the combined influence of band dispersion, transport asymmetry, and thermopower. Pristine CaSiO_3_ exhibits a relatively low PF at low temperature, followed by a gradual increase with increasing temperature. This trend is consistent with the weak transport response of the wide-gap parent phase at low temperature and a larger thermally weighted contribution at higher temperature.

A similar nonmonotonic low-temperature trend is observed for the 12.5% Ta-doped composition, where PF remains small at low temperature but increases rapidly above the intermediate-temperature region. At higher temperatures, this composition surpasses the parent phase, indicating that moderate Ta substitution provides the most favorable balance between enhanced electrical conductivity and retained Seebeck response. This indicates that, among the three modeled compositions, the 12.5% Ta system shows the most favorable calculated balance between carrier transport and Seebeck response. In contrast, the 25% Ta-doped system shows the lowest PF over most of the investigated temperature range, with only a gradual increase at higher temperatures. Although this composition exhibits improved charge transport due to higher dopant concentration, the reduced Seebeck coefficient limits the overall PF. This indicates that excessive Ta incorporation shifts the system toward a more conductivity-dominated transport regime, where the gain in electrical conductivity is counterbalanced by the suppression of thermopower. The smooth PF curves show no abrupt change in the calculated transport response over the sampled temperature range; under the constant-*τ* approximation they do not provide an independent measure of scattering anomalies or phase-like instabilities. The PF trend is therefore interpreted through the competition between the Seebeck magnitude, *σ*/*τ*, and the energy-dependent transport asymmetry near *E*_F_. Moderate Ta substitution appears to optimize this balance, whereas heavier substitution strengthens the conductive response but weakens the Seebeck contribution. This behavior agrees with previous studies on donor-modified oxides, where the power factor is strongly controlled by the competition between carrier activation and scattering effects.^131^ It is also consistent with reports showing that dopant-enhanced electrical transport improves PF only when the Seebeck coefficient is not strongly suppressed.^132^ Within the present constant-*τ* comparison, the 12.5% Ta composition shows the most favorable calculated power-factor response over much of the investigated temperature range; a quantitative device ranking would require a specified relaxation time and experimentally relevant total thermal conductivity. Accordingly, the calculated PF/*τ* trends are used here only for comparative assessment of the three modeled compositions. They do not establish that pristine CaSiO_3_ or either Ta-substituted composition is preferable for a specific thermoelectric or conductivity-based application without an experimentally relevant relaxation time, total thermal conductivity, and application-specific validation.

#### 3.6.3. Thermodynamic-transport synergy index (*ZT*)

The dimensionless thermoelectric figure of merit, *ZT*, quantifies the balance between electrical transport, thermopower, and heat conduction and is defined by eqn (21).21
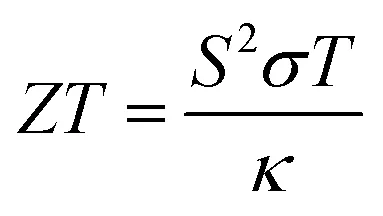


In this relation, the Seebeck coefficient (*S*) represents the voltage generated per unit temperature gradient, while electrical conductivity (*σ*) governs the ease of charge carrier transport. The temperature *T* weights the coupled transport response in the *ZT* expression and is not treated here as direct evidence of thermally activated carriers. A favorable thermoelectric response therefore requires a sufficiently large Seebeck magnitude and electrical conductivity together with a comparatively low thermal-transport contribution. The *ZT* values discussed below are interpreted within the same constant–relaxation–time BoltzTraP framework used for the transport curves in Sections 3.6.1 and 3.6.2, with the calculated thermal-transport quantity used consistently throughout.

**Fig. 17:**
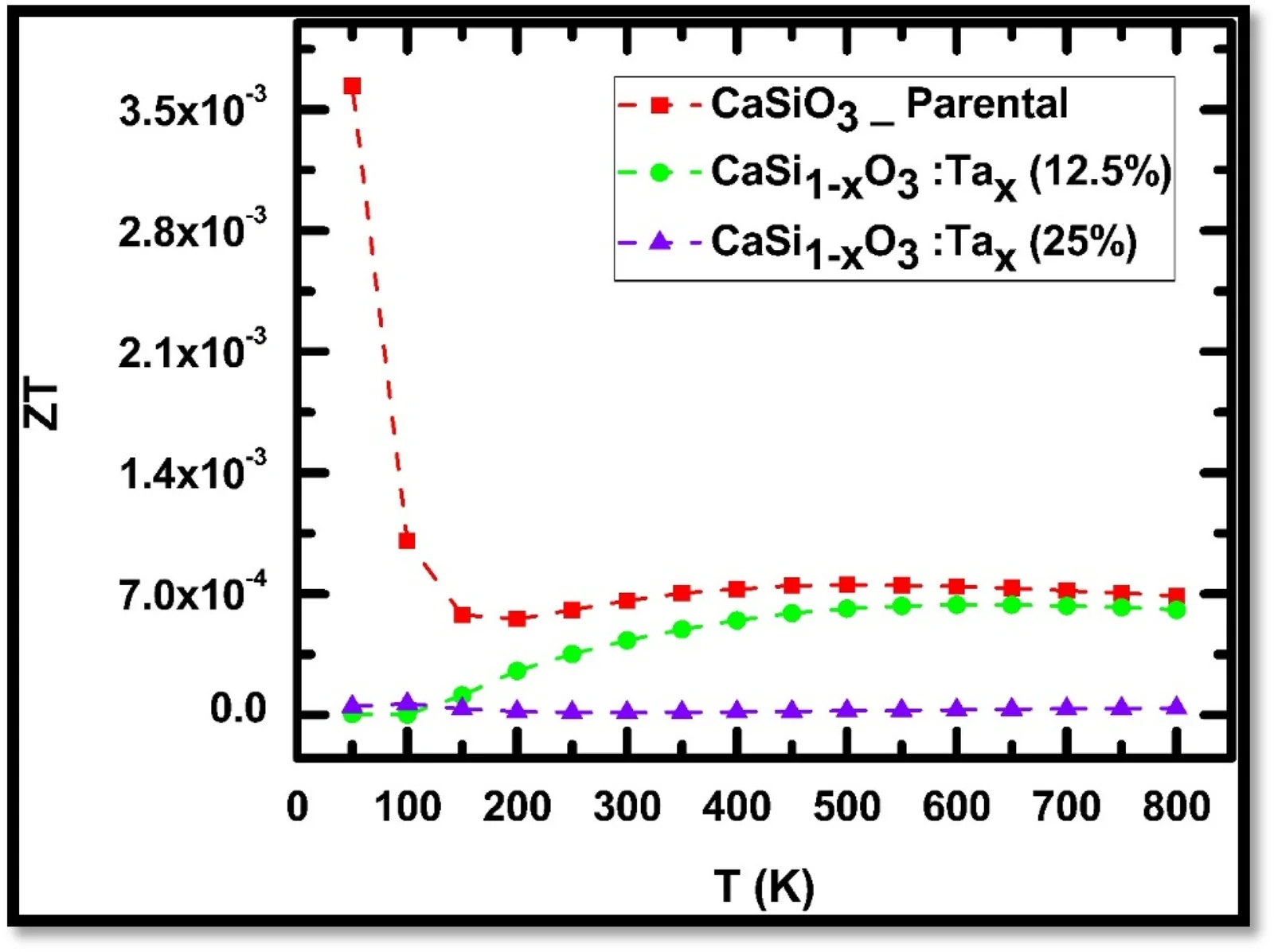
Temperature-dependent figure of merit (*ZT*) of pristine and Ta-doped CaSi_1−*x*_Ta_*x*_O_3_. The relative low-temperature maximum of the parent phase and the suppressed response at higher Ta content show that stronger electrical transport does not translate monotonically into higher *ZT*; the 25% metallic composition remains limited by its small Seebeck magnitude and enhanced electronic heat transport.

As shown in Fig. 17, the thermoelectric figure of merit, *ZT*, of pristine CaSiO_3_ and Ta-substituted CaSi_1−*x*_Ta_*x*_O_3_ exhibits a clear temperature-dependent behavior governed by the combined effects of electrical conductivity, Seebeck coefficient, and thermal conductivity. Pristine CaSiO_3_ displays a relative low-temperature maximum around 50–100 K, followed by a rapid decrease and a much flatter response at higher temperature. Within the calculated transport model, this low-temperature maximum arises from the favorable ratio between the Seebeck contribution and the comparatively small thermal-transport response. Its subsequent reduction is attributed to the evolving balance among *S*, *σ*, and *κ* with temperature; because a constant relaxation time is used, the *ZT* curve is not employed to infer a separate temperature-dependent scattering mechanism.

For the 12.5% Ta-doped composition, the *ZT* value remains lower than that of the parent compound but follows a smoother temperature-dependent trend. This result should not be interpreted as evidence of an insulating or carrier-poor state. The same composition shows electron-like thermopower and an enhanced power-factor response at higher temperature, but the accompanying thermal-transport contribution prevents that electrical improvement from producing a comparable increase in *ZT*. The 12.5% composition therefore represents a transport trade-off: Ta-derived states improve charge transport, while the combined *S*–*σ*–*κ* balance limits the net figure of merit.

In contrast, the 25% Ta-doped system shows very small, nearly zero *ZT* values throughout the full temperature range. This low *ZT* is fully compatible with its metallic electronic character: the composition has the strongest *σ*/*τ* response, but its small Seebeck magnitude and substantially larger electronic heat-transport response suppress the ratio *S*^2^*σT*/*κ*. Thus, the progression with Ta concentration is not a monotonic improvement in thermoelectric efficiency. Increasing Ta strengthens the conductive response, whereas the figure of merit is controlled by how that gain is balanced against thermopower and heat transport.

Donor-modified oxides illustrate the central thermoelectric trade-off: increases in electrical conductivity can be offset by reduced thermopower and greater heat transport, as observed in Nb-doped SrTiO_3_, where increasing donor concentration does not necessarily maximize the power factor.^133,134^ More broadly, thermoelectric performance is governed by the coupled balance among carrier concentration, Fermi-level position, Seebeck response, electrical transport, and thermal conductivity rather than by optimization of any single parameter.^135–137^ For the present compositions, the calculated trends are therefore most appropriately used for comparative transport screening: the 12.5% Ta system exhibits the more balanced combination of thermopower, band-derived conductivity, and thermal transport, whereas the 25% Ta system combines strong conductivity with a strongly suppressed *ZT*. These calculated differences characterize the modeled transport regimes but do not, by themselves, establish application-level thermoelectric performance.

As summarized in Table 8, the thermoelectric transport analysis correlates electrical conductivity, thermal conductivity, Seebeck coefficient, power factor, and *ZT* across pristine and Ta-doped CaSiO_3_ systems. The calculated response is best described as a composition-dependent trade-off rather than a simple conductivity-driven enhancement of thermoelectric performance. Ta substitution strengthens band-derived charge transport, the 12.5% composition provides the most favorable power-factor balance, and the 25% metallic composition remains strongly ZT-limited by its small Seebeck magnitude and larger heat-transport response. This interpretation keeps the transport discussion consistent with the electronic classification established earlier in the manuscript while avoiding an unsupported attribution of the *ZT* trend to temperature-dependent scattering processes that are not resolved under the constant-τ treatment.

**Table 8:** Integrated multi-parameter thermoelectric response framework of Ta-modified CaSi_1−*x*_Ta_*x*_O_3_ comparative evolution of band-derived charge transport, Seebeck response, thermal transport, and calculated *ZT*

Descriptor	CaSiO_3_ _(parent)	CaSi_1−*x*_Ta_*x*_O_3_ (*x* = 12.5%)	CaSi_1−*x*_Ta_*x*_O_3_ (*x* = 25%)	Trend morphology	Governing physics	Transport interpretation
Electrical conductivity (*σ*/*τ*)	Low; weak *T* dependence	Moderate; slightly increasing	High; weakly *T* dependent	Low → moderate → high	Band dispersion and Ta-derived states near EF	Charge transport strengthened with Ta
Electronic thermal-transport response (*κ*)	Low; gradual rise	Moderate increase	Strong increase	Increasing response with Ta	Band-derived electronic heat transport	Higher heat transport offsets part of the conductivity gain
Seebeck coefficient (*S*)	Positive; increasing	Negative; decreasing	Small; weak positive	Composition-dependent sign and magnitude	Transport asymmetry around EF	Seebeck-sign modulation; not an electronic-classification criterion
Power factor (PF/*τ*)	Moderate growth	Strongest response at higher *T*	Suppressed response	Best balance at 12.5% Ta	*S* ^2^–*σ*/*τ* trade-off	Most favorable comparative PF at moderate Ta
figure of merit (*ZT*)	Relative low-*T* maximum	Reduced; smooth	Near-zero	Non-monotonic with Ta content	Balance among *S*, *σ*, *T*, and *κ* in the transport model	High conductivity alone does not maximize *ZT*

### 3.7. Elastic stiffening, anisotropic reinforcement, and mechanical stability

The technological applicability of functional oxides is strongly governed by their mechanical reliability, because structural failure under external loading can limit their use in practical devices. For pristine CaSiO_3_ and Ta-substituted CaSi_1−*x*_Ta_*x*_O_3_, the evolution of elastic constants, mechanical moduli, hardness, Debye temperature, Poisson's ratio, and anisotropy factor provides a comprehensive description of the lattice response under mechanical perturbation.^138^ For cubic CaSiO_3_ specifically, first-principles calculations have shown that the independent elastic constants *C*_11_, *C*_12_, and *C*_44_, together with the derived elastic moduli, provide a quantitative basis for characterizing its elastic response.^139^

**Fig. 18:**
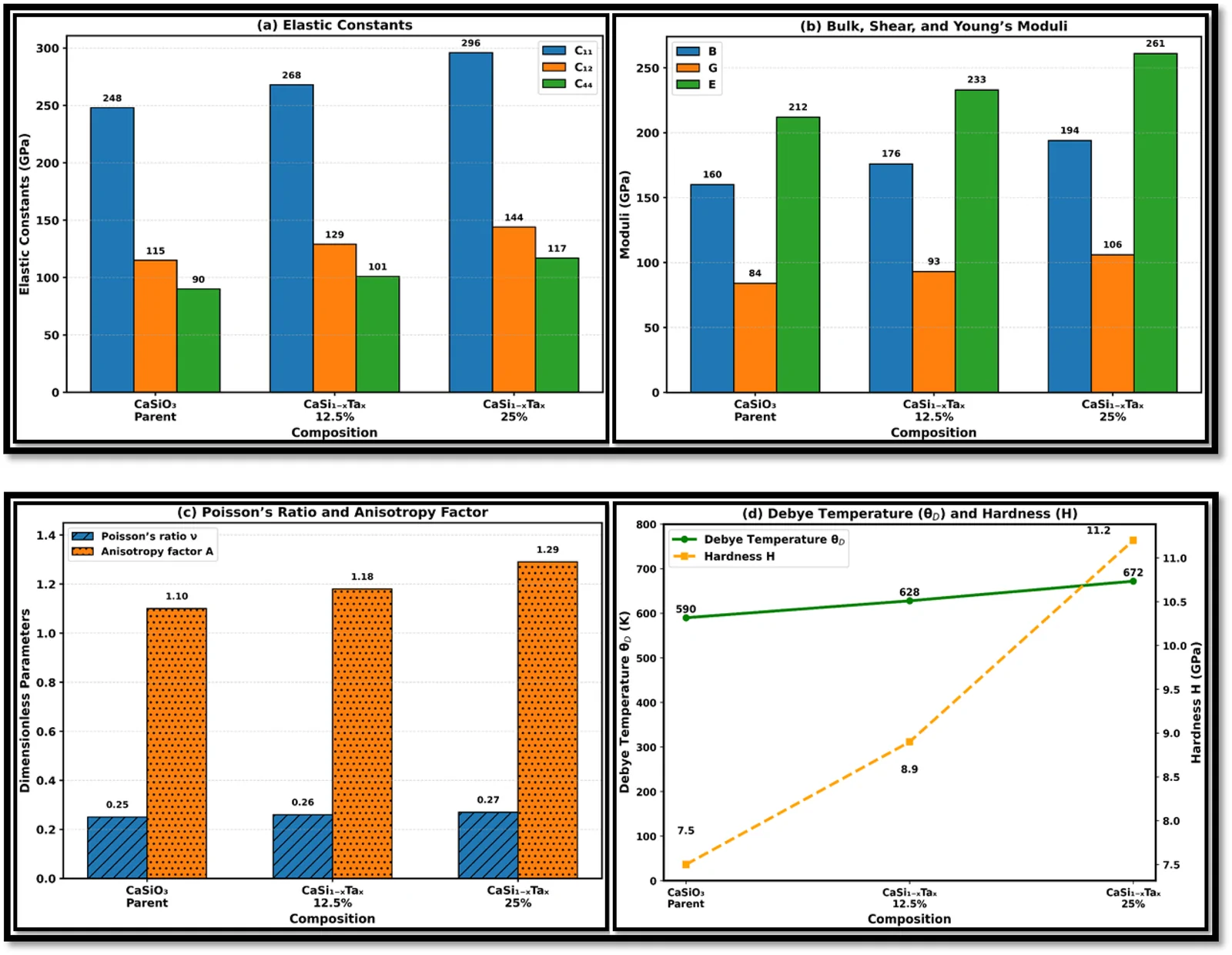
(a) Elastic constants, (b) bulk, shear, and Young's moduli, (c) Poisson's ratio and anisotropy factor, and (d) Debye temperature (*θ*_D_) and hardness (*H*) of CaSi_1−*x*_Ta_*x*_O_3_. The elastic-derived quantities describe the property-level response to Ta substitution and are interpreted together with the structural changes in Section 3.1 and the independent COHP/ELF bonding descriptors in Section 3.4.5. These small-strain trends are treated separately from the EOS-derived hydrostatic bulk modulus and are not used independently to infer monotonic Ta–O bond strengthening.

As shown in Fig. 18(a–d) and summarized in Table 9, the elastic and mechanical descriptors evolve systematically with Ta concentration; however, these trends are treated as mechanical-response quantities and not as direct measures of Ta–O bond strength or covalency. Pristine CaSiO_3_ exhibits moderate rigidity within the structurally intact corner-sharing SiO_6_ framework. As established in Section 3.1, Ta-for-Si substitution produces lattice expansion, chemically inequivalent B–O coordination environments, and concentration-dependent local relaxation while preserving the underlying perovskite topology. After Ta substitution, the elastic constants *C*_11_, *C*_12_, and *C*_44_ increase progressively, indicating enhanced resistance against longitudinal deformation, elastic coupling of normal strains, and shear deformation, respectively. Accordingly, the elastic stiffening is considered as the macroscopic mechanical response to a structurally and electronically reorganized B–O network, rather than as evidence obtained from the mechanical data alone for monotonic strengthening of individual Ta–O bonds.

**Table 9:** Mechanical properties of pristine and Ta-substituted CaSi_1−*x*_Ta_*x*_O_3_, showing composition-dependent changes in elastic stiffness, hardness, anisotropy, and vibrational response; the reported mechanical quantities are interpreted as property descriptors rather than direct measures of Ta–O bond strength

Property	CaSiO_3_ (parent)	CaSi_1−*x*_Ta_*x*_ (12.5%)	CaSi_1−*x*_Ta_*x*_ (25%)	Elastic/mechanical interpretation
*C*_11_ (GPa)	248	268	296	Higher *C*_11_ indicates increased longitudinal elastic stiffness
*C*_12_ (GPa)	115	129	144	Higher *C*_12_ reflects increased elastic coupling between normal strain components
*C*_44_ (GPa)	90	101	117	Higher *C*_44_ indicates increased shear rigidity
Bulk modulus *B* (GPa)	160	176	194	Elastic-constant-derived bulk response; interpreted separately from EOS B_0_
Shear modulus *G* (GPa)	84	93	106	Higher resistance to shape deformation in the elastic response
Young's modulus *E* (GPa)	212	233	261	Higher tensile/axial elastic stiffness
Poisson's ratio *ν*	0.25	0.26	0.27	Modest increase in transverse elastic response
Anisotropy factor A	1.10	1.18	1.29	Increasing elastic anisotropy; not a direct measure of bond strength
Debye temperature *θ*_D_ (K)	590	628	672	Higher characteristic vibrational scale within the elastic-Debye model
Hardness *H* (GPa)	7.5	8.9	11.2	Higher predicted indentation hardness

The bonding analyses in Section 3.4.5 provide the microscopic context for this interpretation: COHP establishes a finite Ta–O bond interaction in the relevant energy range, while ELF shows the accompanying real-space redistribution around the Ta/O environment. These results support a composition-dependent reorganization of the local bonding environment, but, in the absence of an integrated bond-strength metric such as ICOHP, they do not justify the stronger claim that every Ta–O bond becomes progressively more covalent or stronger with increasing Ta content. When combined with the structural results of Section 3.1, the COHP and ELF analyses provide the intermediate bonding link between Ta-induced local coordination changes and the mechanical response: structural substitution modifies the B–O environment, COHP/ELF resolve the corresponding change in the Ta–O electronic interaction and spatial charge distribution, and the elastic tensor quantifies the resulting small-strain mechanical response. Thus, the proposed structure–bonding–property relationship is based on mutually consistent but physically distinct descriptors rather than on a direct conversion of elastic constants into a bond-strength measure.

The increase in bulk modulus *B*, shear modulus *G*, and Young's modulus *E* further confirms that the elastic-constant-derived small-strain response becomes stiffer with increasing Ta content. Importantly, the elastic-derived bulk modulus reported here (160, 176, and 194 GPa) is not treated as interchangeable with the EOS bulk modulus *B*_0_ discussed in Section 3.1 (215.3005, 167.1973, and 169.5482 GPa). The former is obtained from the elastic-constant response, whereas *B*_0_ is obtained from the curvature of the hydrostatic energy–volume relation. The two analyses therefore show different trends and are reported separately; their numerical disagreement is not used as evidence for stronger Ta–O bonding. The gradual rise in Poisson's ratio from 0.25 to 0.27 indicates only a modest change in the transverse elastic response and is not, by itself, taken as a criterion for bond strengthening. The increasing anisotropy factor A indicates a progressively more anisotropic elastic response, rather than directly proving stronger direction-dependent bonding. In addition, the increase in Debye temperature from 590 K to 672 K corresponds to a higher characteristic vibrational scale within the elastic-Debye description, while the rise in hardness from 7.5 GPa to 11.2 GPa indicates a higher predicted resistance to indentation. Taken together, these elastic-derived trends show that Ta substitution modifies and, for several small-strain descriptors, increases the mechanical rigidity of the modeled structures, while the EOS results separately indicate that both Ta-substituted compositions are more compressible than pristine CaSiO_3_ under hydrostatic volume changes. This apparent difference does not constitute a contradiction in bonding behavior because the EOS and elastic-tensor analyses probe distinct deformation responses and neither is used independently to determine chemical bond strength.

The calculated elastic constants (*C*_11_, *C*_12_ and *C*_44_) indicate that the material has strong resistance to compressive deformation, while its comparatively lower shear response reflects the typical brittle nature of oxide ceramics. This behavior is consistent with previous reports describing CaSiO_3_ as a mechanically stiff but inherently brittle material.^127^ At 12.5% Ta substitution, Ta incorporation at the Si site modifies the local B–O coordination environment identified structurally in Section 3.1. The nominal Ta^5+^/Si^4+^ notation, where used elsewhere in the manuscript, is only a formal electron-counting description and is not invoked here as a quantitatively determined atomic charge. The increase in the elastic constants and mechanical moduli is therefore discussed together with the local structural distortion and the Ta–O electronic redistribution supported by the COHP/ELF analysis, rather than being assigned to an unquantified increase in Ta–O covalency. Consequently, the bulk, shear, and Young's moduli increase, indicating enhanced stiffness, improved resistance to deformation, and reduced compressibility within the elastic-constant-derived response. This distinction avoids using the mechanical data themselves as proof of a specific chemical-bond-strengthening mechanism.^140,141^ At this composition, the structure–property connection is therefore expressed as a response of the elastic tensor to the modified local coordination and associated electronic redistribution, without assigning a quantitative increase in individual Ta–O bond strength.

At 25% Ta substitution, the number of Ta-centered coordination environments increases within the same supercell framework, producing a more extensive perturbation of the B–O network. Consistently, Section 3.4.5 shows broader Ta–O COHP participation and greater spatial continuity of intermediate-ELF regions through Ta/O-containing environments. As a result, this composition exhibits the highest elastic moduli, hardness, and Debye temperature among the three compositions in the elastic-derived analysis. The increase in these mechanical descriptors is therefore correlated with the larger extent of structural and electronic reorganization at the higher Ta concentration, rather than being assigned to universally stronger local Ta–O bonds. The slight increase in Poisson's ratio describes the evolution of the transverse elastic response but is not used independently to establish ductile or brittle behavior, while the rising anisotropy factor indicates increasing elastic directionality rather than serving as a direct measure of structural distortion or bond strength. The mechanical data are therefore interpreted as evidence of a concentration-dependent change in elastic rigidity and anisotropy, not as standalone proof that all local Ta–O bonds become progressively stronger.

Across the three compositions, pristine CaSiO_3_ remains moderately stiff, nearly isotropic, and brittle, with comparatively limited shear resistance. At 12.5% Ta, CaSi_1−*x*_Ta_*x*_O_3_ shows increased elastic-derived stiffness and shear resistance associated with Ta-induced local coordination changes and electronic redistribution, whereas at 25% Ta it exhibits the highest elastic-derived moduli, hardness, anisotropy factor, and Debye temperature among the modeled compositions. These concentration-dependent elastic trends remain distinct from the EOS-derived hydrostatic bulk-modulus response. Taken together, the calculations establish the following physically consistent relationship within the scope of the present study: Ta substitution first modifies the local B–O structural environment (Section 3.1), this modification is accompanied by a composition-dependent Ta–O electronic-interaction and localization response revealed independently by COHP and ELF (Section 3.4.5), and the resulting substituted structures exhibit corresponding changes in their calculated elastic stiffness, anisotropy, and hardness (Section 3.7). This sequence provides the structure–bonding–property connection without requiring the unsupported assumption of monotonically increasing Ta–O covalency.

The calculated elastic moduli, hardness, and anisotropy trends suggest that Ta-substituted CaSi_1−*x*_Ta_*x*_O_3_ may merit consideration for mechanically demanding oxide environments. However, application-level claims are kept qualitative because practical suitability for coatings, micromechanical systems, or energy-conversion devices would require experimental validation and additional assessment of fracture, fatigue, thermal-cycling, and finite-temperature mechanical behavior.

The detailed magnetic and spin-density analyses have been transferred to the SI to improve the focus and conciseness of the main manuscript. The complete magnetic-response discussion, including site-resolved and interstitial magnetic moments, is provided in Supplementary Section S1 (Fig. S1 and Table S1), while the corresponding real-space spin-density analysis is presented in SI Section S2 (Fig. S2 and Table S2).

## 4. Conclusion

This work establishes, within a consistent first-principles framework, how aliovalent Ta-for-Si substitution modifies the structural, energetic, electronic, optical, thermoelectric, mechanical, and magnetic responses of CaSiO_3_. The analysis combines Ta-induced electronic reconstruction, phonon stability, Fermi-surface topology, and nominal donor-density evolution, COHP/ELF bonding descriptors, transport behavior, and spin-density response. The calculated structural results confirm that Ta substitution preserves the fundamental CaSiO_3_ framework while inducing controlled lattice expansion, chemically inequivalent local B–O coordination environments and concentration-dependent local relaxation. The equilibrium volume increases from 2533.4554 Bohr^3^ for pristine CaSiO_3_ to 2755.1025 and 3155.7362 Bohr^3^ for the 12.5% and 25% Ta-substituted systems, respectively, whereas the Birch–Murnaghan bulk modulus decreases from 215.3005 GPa to 167.1973 and 169.5482 GPa, indicating greater hydrostatic compressibility of both substituted compositions relative to pristine CaSiO_3_, with a slight recovery at 25% Ta. The negative formation energies of all compositions (−4.11, −3.28, and −2.96 eV per atom), support the energetic feasibility of the modeled compositions relative to the chosen elemental reference states, while the absence of imaginary phonon modes establishes their dynamical stability within the investigated structures. The electronic-structure analysis shows that pristine CaSiO_3_ retains its wide-band-gap semiconducting character, with an indirect band gap of 3.432 eV. In contrast, Ta substitution introduces Ta-5d-dominated donor-derived states with O-2p participation near the Fermi level, producing a degenerate n-type/incipient metallic regime at 12.5% Ta and a metallic n-type regime at 25% Ta. The Fermi-surface analysis independently supports this concentration-dependent electronic evolution, while the nominal donor-density estimates increase from the negligible-carrier parent limit to 2.45 × 10^21^ cm^−3^ and 4.28 × 10^21^ cm^−3^ for the 12.5% and 25% Ta-substituted models, respectively. These values represent stoichiometric donor-density estimates and are not interpreted as independently calculated equilibrium free-carrier concentrations. The same electronic reconstruction is reflected in the calculated interband optical response, where Ta incorporation smooths the dielectric and absorption spectra, redistributes spectral weight, and modifies the high-energy and plasmon-related features. The thermoelectric response is non-monotonic: the 12.5% composition provides the most favorable calculated PF/τ balance over much of the investigated temperature range, whereas 25% Ta produces the strongest *σ*/*τ* and electronic heat-transport response but a strongly suppressed *ZT* because of its small Seebeck magnitude. Thus, enhanced Ta-induced charge transport does not by itself imply improved thermoelectric performance. Mechanically, Ta substitution produces a composition-dependent small-strain response that is interpreted through a structure–bonding–property relationship rather than a simple bond-strengthening picture. The local B–O coordination changes are accompanied by a finite, composition-dependent Ta–O COHP response and ELF redistribution, while the elastic tensor independently shows increasing stiffness, shear resistance, Debye temperature, and predicted hardness. Because integrated COHP values were not evaluated, neither the bonding descriptors nor the mechanical quantities are used to claim a monotonic increase in individual Ta–O bond strength or covalency. The 25% Ta-substituted composition exhibits the largest elastic-derived moduli and hardness among the investigated compositions, whereas the EOS-derived hydrostatic bulk modulus remains lower than that of pristine CaSiO_3_. Magnetically, pristine CaSiO_3_ remains non-magnetic, whereas 12.5% Ta substitution produces a finite total magnetic moment of 0.43417 *µ*_B_ with an interstitial contribution of 0.22036 *µ*_B_. The site-resolved analysis shows that the Ta-centered muffin-tin region carries the largest individual atom-centered moment of 0.08259 *µ*_B_, while the neighboring O sites contribute smaller positive moments. With the spin-resolved PDOS and real-space spin-density distribution, these values show that the finite response is spatially distributed over the Ta/O-centered electronic environment and the interstitial region rather than confined to an isolated Ta atom. At 25% Ta, the total magnetic moment is almost completely quenched to −0.00132 *µ*_B_ and the interstitial contribution falls to 0.00001 *µ*_B_. The two Ta-centered moments decrease to −0.00017 and −0.00041 *µ*_B_, whereas the O-site moments become extremely small and mixed in sign. The reduced spin-channel asymmetry, very small mixed-sign local moments, and weaker spin-density contrast consistently attribute this near-quenching to spatial compensation of the local spin polarization; no specific Ta–Ta exchange interaction or long-range magnetic-ordering mechanism is inferred from the present calculations. The present results show that Ta-for-Si substitution produces a systematic concentration-dependent evolution of the electronic, optical, transport, mechanical, and magnetic responses while retaining the underlying CaSiO_3_ perovskite framework. These findings describe the intrinsic behavior of the modeled stoichiometric, defect-free structures and should not be interpreted as evidence of device-level performance. Future investigations should therefore examine intermediate Ta concentrations, explicit defect-compensation energetics, strain-dependent responses, and application-specific behavior, together with experimental validation of the predicted electronic, transport, mechanical, and magnetic properties.

## Conflicts of interest

There are no conflicts to declare.

## Supplementary Material

RA-OLF-D6RA06904E-s001

## Data Availability

The data supporting the findings of this study, including the optimized structural parameters, formation energies, electronic band structures, density of states, Fermi-surface analysis, carrier-concentration estimates, optical spectra, thermoelectric parameters, mechanical properties, magnetic moments, spin-density analysis, and piezoelectric coefficients, are included within the article. No separate supplementary information (SI) file is associated with this manuscript. Additional supporting details may be made available from the corresponding author upon reasonable request. Supplementary information is available. See DOI: https://doi.org/10.1039/d6ra06904e.
