# Supplementary material for: Ta-induced electronic reconstruction in CaSiO3: wide-band-gap modulation, emergent metallic behavior, and multifunctional response from first-principles
Source: RSC Adv. 2026 Sep 1;16(42):51560–603. doi: 10.1039/d6ra06904e (PMC13531786; doi:10.1039/d6ra06904e)
Supplement: Supplementary file 1 [file RA-016-D6RA06904E-s001.pdf]

Supplementary Information (SI) for RSC Advances.
This journal is © The Royal Society of Chemistry 2026

Supplementary Information

Ta-Induced Electronic Reconstruction in $CaSiO_3$: Wide-Band-Gap Modulation, Emergent Metallic Behavior, and Multifunctional Response from First-Principles

Qaiser Rafiq*[1], Sikander Azam*[1,5], Khalid M. Elhindi[2], Muhammad Tahir Khan[1], Sisi Wang*[3], Umair Rashid[4]

[1] Department of Physics, Riphah International University, Islamabad, Pakistan

[2]Plant Production Department, College of Food & Agriculture Sciences, King Saud University, P.O. Box 2460, Riyadh 11451, Saudi Arabia

[3]Key Laboratory of Urban Rail Transit Intelligent Operation and Maintenance Technology & Equipment of Zhejiang Province, College of Engineering, Zhejiang Normal University, Jinhua 321004, People's Republic of China

[4]School of Physics and Optoelectronic Engineering, Beijing University of Technology, Beijing 100124, China

[5]New Technologies-Research Center, University of West Bohemia, Univerzitni 8, 306 14, Pilsen, Czech Republic

***Corresponding Authors:** Qaiser Rafiq (qrafique1@gmail.com)
Sikander Azam (physicst.sikander@gmail.com)
Sisi Wang (sisi.wang@zjnu.edu.cn)

Contents:

Section S1. Spin Polarization and Magnetic Response Evolution

Section S2. Spin-Density Distribution in Ta-Substituted $CaSiO_3$

References

S1. Spin Polarization and Magnetic Response Evolution

Pristine $CaSiO_3$ is non-magnetic, which can be attributed to the closed-shell electronic configurations of Ca^{2+} and Si^{4+} and the absence of partially filled magnetic orbitals. As a result, spin polarization is suppressed throughout the lattice, and both the total magnetic moment and interstitial contribution remain nearly zero. Within the present spin-polarized DFT treatment, this

behavior is therefore described as a non-magnetic, spin-balanced state rather than as direct evidence of orbital diamagnetism. This is consistent with transition-metal-free oxide perovskites [1]. Upon Ta substitution at the Si site, a distinct magnetic response appears. For the 12.5% Ta-doped composition, finite spin polarization develops, with a total magnetic moment of 0.43417 μ_B and an interstitial magnetic moment of 0.22036 μ_B. This indicates that the magnetization is not confined only to the Ta site but is partially extended into the surrounding oxygen network and interstitial regions. The site-resolved magnetic moments provide direct microscopic evidence for this distribution. The Ta site carries the largest individual atom-centered moment of 0.08259 μ_B, while smaller moments are obtained for the inequivalent Ca, Si, and O environments. In particular, the inequivalent O sites carry moments of 0.00181, 0.00099, and 0.00047 μ_B. Together with the substantial interstitial contribution, these values show that the induced spin polarization is spatially distributed rather than confined to an isolated Ta atom. The origin of this response is associated with partially occupied Ta-derived 5d states and their interaction with O-2p states, which produce a localized but spatially distributed spin-density pattern. This interpretation is consistent with the spin-resolved Ta-5d/O-2p electronic states discussed in Section 3.4.2 and is further examined through the real-space spin-density distribution in Section S2. Similar extended spin-polarized states have been reported in doped oxide systems, where dopant-derived carriers mediate magnetic polarization across the lattice [2]. At the higher Ta concentration of 25%, the magnetic response is strongly suppressed, with the total magnetic moment decreasing to −0.00132 μ_B and the interstitial contribution reduced to 0.00001 μ_B**.** The site-resolved moments also collapse to nearly zero: the two inequivalent Ta sites carry −0.00017 and −0.00041 μ_B, while the inequivalent O sites show small mixed-sign moments of +0.00009, −0.00005, and −0.00009 μ_B. The coexistence of very small positive and negative local contributions demonstrates strong spatial compensation of the spin polarization at the higher Ta concentration. This behavior is consistent with increased overlap among the Ta-centered electronic regions and with the substantially reduced spin-channel asymmetry observed in the spin-resolved DOS; however, the present calculations do not quantify a specific Ta–Ta magnetic exchange interaction. Such concentration-dependent redistribution and cancellation of local spin contributions have also been reported in highly doped oxide systems [3,4]. Accordingly, the suppression at 25% Ta is attributed here to the calculated reduction and compensation of local spin polarization rather than to an assumed long-range magnetic-ordering mechanism. Overall, the magnetic behavior evolves from a non-magnetic parent phase to the

largest calculated net spin polarization among the investigated compositions at 12.5% Ta, followed by strong suppression of magnetism at 25% Ta substitution. The combined spin-resolved PDOS, site-resolved magnetic moments, and real-space spin-density results therefore provide a consistent microscopic picture: Ta incorporation initially generates a distributed Ta/O-associated spin imbalance, whereas increasing the Ta content produces much smaller, mixed-sign local moments and an almost complete cancellation of the cell-integrated spin polarization. This trend is shown in Fig. S1, and summarized quantitatively in Table S1.

- $CaSiO_3$ (Parent): Completely non-magnetic
- $CaSi_{1-x}Ta_xO_3$ (12.5%): Finite, spatially distributed spin polarization with the largest net moment among the investigated compositions
- $CaSi_{1-x}Ta_xO_3$ (25%): Nearly quenched net spin polarization arising from strongly compensated local magnetic moments

Materials with weak but tunable magnetism, such as Ta-doped $CaSiO_3$, may warrant further investigation for spin-dependent oxide functionality. However, the present calculations establish a concentration-dependent spin-polarization response rather than device-level magnetic or spin-transport performance.

Table S1. Spin magnetic characteristics of pristine and Ta-substituted $CaSi_{1-x}Ta_xO_3$, including site-resolved, interstitial, and total magnetic moments used to characterize the microscopic evolution of spin polarization with Ta concentration

Materials	Site-resolved muffin-tin magnetic moments (μ_B)	Interstitial magnetic moment (μ_B)	Total magnetic moment (μ_B)	Magnetic behavior insight
**$CaSiO_3$ (Parent)**	≈0; spin-balanced parent state	0.00000	0.00000	Non-magnetic; absence of resolved spin polarization
**$CaSi_{1-x}Ta_x$ (12.5%)**	Ca: 0.01030; Ta: 0.08259; Si: 0.00239, 0.00403, 0.00402; O: 0.00181, 0.00099, 0.00047	0.22036	0.43417	Finite distributed spin polarization; largest individual atom-centered moment occurs on Ta, with additional lattice and interstitial Contributions
**$CaSi_{1-x}Ta_x$ (25%)**	Ca: 0.00008; Ta: −0.00017,	0.00001	−0.00132	Nearly quenched net spin polarization with very small mixed-sign local moments, indicating

	−0.00041; Si: 0.00002, −0.00002; O: +0.00009, −0.00005, −0.00009			strong compensation

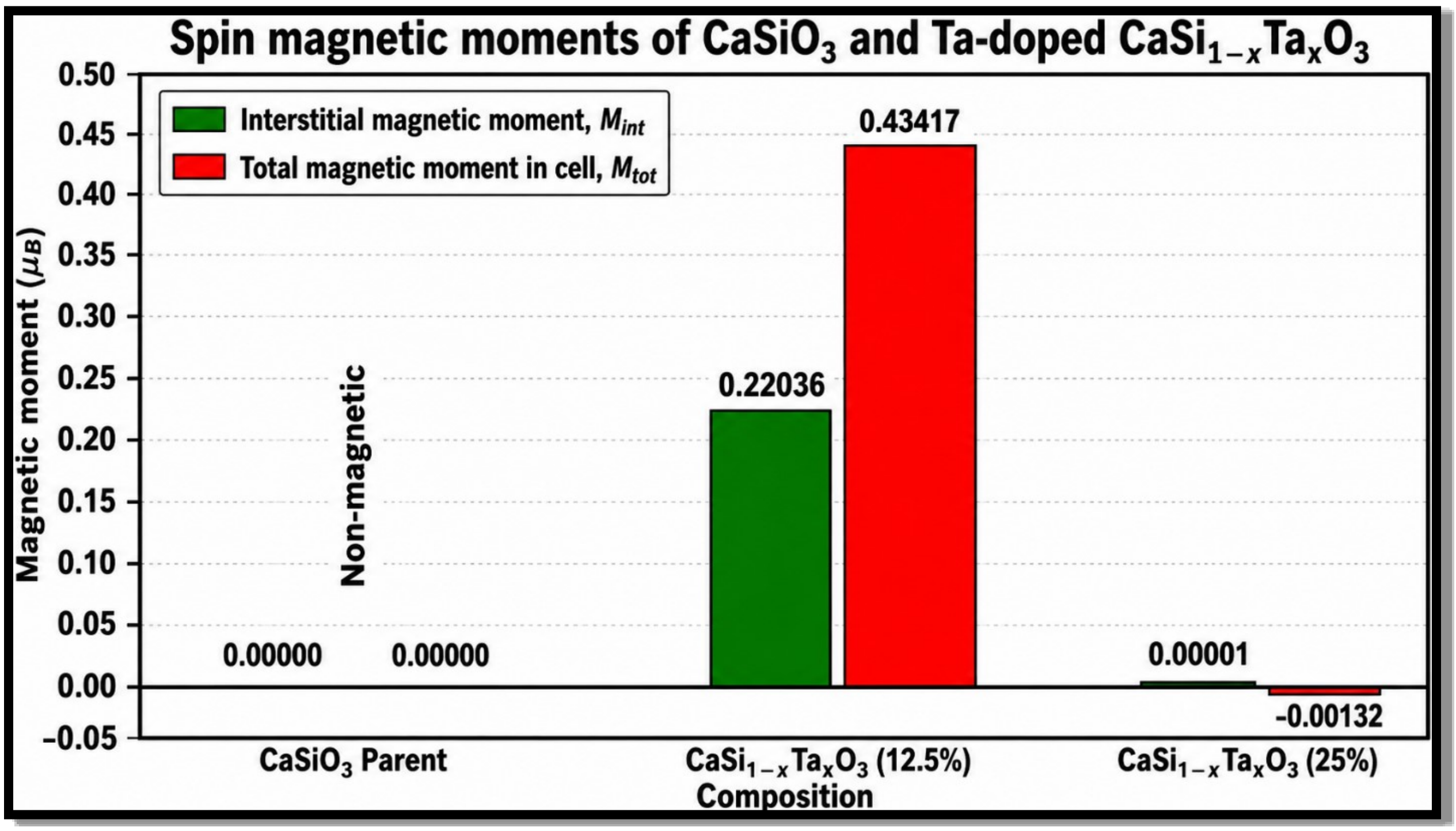

Fig. S1. Spin magnetic moments of $CaSiO_3$ and Ta-substituted $CaSi_{1-x}Ta_xO_3$, showing the interstitial (M_{int}) and total-cell (M_{tot}) contributions. The parent phase remains non-magnetic, whereas 12.5% Ta produces a finite total moment of 0.43417 μ_B with an interstitial contribution of 0.22036 μ_B. At 25% Ta, M_{tot} decreases to −0.00132 μ_B and M_{int} to 0.00001 µB, consistent with the strongly reduced and mutually compensating site-resolved magnetic moments at the higher Ta concentration.

S2. Spin-Density Distribution in Ta-Substituted $CaSiO_3$

To better understand the source of spin polarization in $CaSiO_3$, spin-density maps were calculated for the pristine $CaSiO_3$ structure and for Ta-substituted $CaSi_{1-x}Ta_xO_3$ systems with Ta concentrations of 12.5% and 25%, as shown in Fig. S2 (a–c). The spin-density difference was

taken as $\rho_s = \rho\uparrow - \rho\downarrow$ with $\rho s > 0$ indicating positive spin density and $\rho s < 0$ indicating negative spin density. This real-space representation identifies the spatial regions in which the two spin channels become locally imbalanced and therefore complements the spin-resolved DOS/PDOS and site-resolved magnetic moments discussed in Sections 3.4.2 and S1, respectively. This type of real-space spin-density mapping is useful for identifying the locality of spin imbalance in perovskite-type oxides [5].

The $CaSiO_3$ parent structure exhibits no discernible spin-density build-up, attesting to the fact that the pristine system does not exhibit local spin imbalance. This spin-balanced real-space distribution is consistent with the vanishing total and interstitial magnetic moments of the parent composition. After 12.5% Ta substitution, the spin-density contrast is concentrated mainly in the Ta-centered region and around the neighboring oxygen environment. This localization is consistent with the finite total magnetic moment of 0.43417 μ_B and the substantial interstitial contribution of 0.22036 μ_B. The site-resolved analysis further shows that the Ta-centered muffin-tin region carries a moment of 0.08259 μ_B, whereas the inequivalent O sites exhibit smaller positive contributions of 0.00181, 0.00099, and 0.00047 μ_B. These results demonstrate that the induced spin polarization is distributed over the Ta/O-centered electronic environment and the interstitial region rather than being confined to an isolated Ta atom. Together with the spin-resolved PDOS, the real-space distribution associates the finite 12.5% Ta response primarily with Ta-5d-dominated near-Fermi-level states accompanied by O-2p participation [6].

The spin-density contrast reduces while the spatially compensated pattern becomes more evident at 25% Ta substitution. This evolution is quantitatively consistent with the collapse of the magnetic response: the total moment decreases to −0.00132 μ_B and the interstitial contribution to 0.00001 μB, while the two Ta-centered moments are reduced to −0.00017 and −0.00041 μ_B. The inequivalent O sites likewise exhibit very small mixed-sign moments of +0.00009, −0.00005, and −0.00009 μ_B. The small magnitudes and opposite signs of these local contributions provide direct evidence for strong spatial compensation of the spin polarization at the higher Ta concentration. Accordingly, the weaker contrast in Fig. S2(c) is interpreted as redistribution and cancellation among multiple Ta/O-centered spin-polarized regions rather than as evidence for a specifically quantified Ta–Ta exchange interaction. From this result, a clear concentration-dependent evolution is obtained: no discernible spin-density imbalance in the parent structure, pronounced localized spin-density contrast at 12.5% Ta, and weaker, more compensated spin density at 25% Ta [7]. This

real-space evolution is consistent with the reduced spin-channel asymmetry in the spin-resolved PDOS and explains the near-quenching of the net magnetic moment at 25% Ta without invoking an unsupported long-range magnetic-ordering mechanism.

These spin-density features, dominant regions, and interpretations are summarized in Table S2. Taken together, the spin-resolved PDOS, site-resolved magnetic moments, and real-space spin-density distributions provide mutually consistent microscopic evidence for the origin of the finite magnetic response at 12.5% Ta and its suppression at 25% Ta.

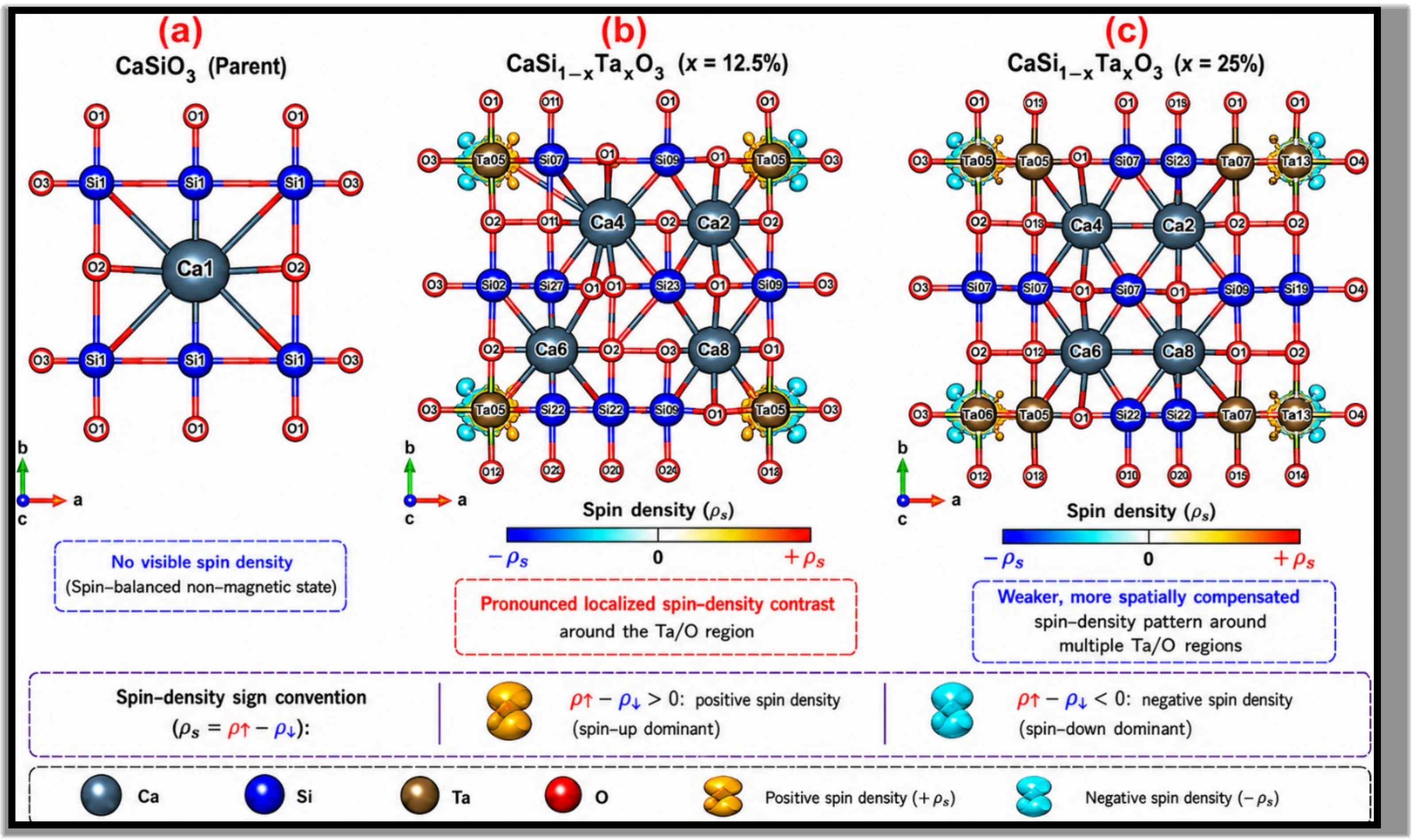

Fig. S2. Spin-density distribution of (a) pristine $CaSiO_3$, (b) $CaSi_{1-x}Ta_xO_3$ (x = 12.5%), and (c) $CaSi_{1-x}Ta_xO_3$ (x = 25%). The parent phase shows no discernible spin-density accumulation. At 12.5% Ta, pronounced positive and negative spin-density features are concentrated mainly around the Ta/O-centered region, consistent with the finite calculated magnetic moment. At 25% Ta, the spin-density contrast becomes substantially weaker and

more spatially compensated, consistent with the nearly quenched total moment and the very small mixed-sign Ta/O site moments. Yellow isosurfaces denote positive spin density ($\rho\uparrow - \rho\downarrow > 0$), whereas cyan isosurfaces denote negative spin density ($\rho\uparrow - \rho\downarrow < 0$)

Table S2. Comparative summary of the real-space spin-density response in pristine and Ta-substituted $CaSi_{1-x}Ta_xO_3$ and its correspondence with the calculated magnetic moments

Materials	Main spin-density feature	Dominant region	Spin-density interpretation
**$CaSiO_3$ (Parent)**	No visible spin-density accumulation	Parent Ca–Si–O framework	Spin-balanced non-magnetic state, consistent with $M_{tot} = 0.00000\ \mu_B$
**$CaSi_{1-x}Ta_x$ (12.5%)**	Strong localized positive/negative spin-density contrast	Ta–O coordination region	Distributed Ta/O-associated spin imbalance, consistent with Mtot = 0.43417 μ_B and the largest atom-centered contribution occurring on Ta
**$CaSi_{1-x}Ta_x$ (25%)**	Weaker and more compensated spin-density pattern	Multiple Ta–O regions	Strong compensation of very small mixed-sign local moments, consistent with $M_{tot} = -0.00132\ \mu_B$

References

[1] H. Jin, K. Lee, S.-H. Baek, J.-S. Kim, B.-K. Cheong, B. H. Park, S. Yoon, B. J. Suh, C. Kim, S. S. A. Seo and S. Lee, Sci. Rep., 2016, 6, 34295, DOI: 10.1038/srep34295.

[2] S. W. Cho, M. Lee, S. Woo, K. Yim, S. Han, W. S. Choi and S. Lee, Sci. Rep., 2018, 8, 5739, DOI: 10.1038/s41598-018-23967-2.

[3] K. Sato, L. Bergqvist, J. Kudrnovský, P. H. Dederichs, O. Eriksson, I. Turek, B. Sanyal, G. Bouzerar, H. Katayama-Yoshida, V. A. Dinh, T. Fukushima, H. Kizaki and R. Zeller, Rev. Mod. Phys., 2010, 82, 1633–1690, DOI: 10.1103/RevModPhys.82.1633.

[4] T. Dietl and H. Ohno, Rev. Mod. Phys., 2014, 86, 187–251, DOI: 10.1103/RevModPhys.86.187.

[5] F. Menescardi and D. Ceresoli, Appl. Sci., 2021, 11, 616, DOI: 10.3390/app11020616.

[6] M. U. Rehman, Q. Wang and Y. Yu, Crystals, 2022, 12, 1597, DOI: 10.3390/cryst12111597.

[7] S. Mehmood and Z. Ali, Appl. Phys. A, 2023, 129, 76, DOI: 10.1007/s00339-022-06352-9.
